# Investigation of Naphthyl–Polyamine Conjugates as Antimicrobials and Antibiotic Enhancers

**DOI:** 10.3390/antibiotics12061014

**Published:** 2023-06-05

**Authors:** Melissa M. Cadelis, Liam R. Edmeades, Dan Chen, Evangelene S. Gill, Kyle Fraser, Florent Rouvier, Marie-Lise Bourguet-Kondracki, Jean Michel Brunel, Brent R. Copp

**Affiliations:** 1 School of Chemical Sciences, The University of Auckland, Private Bag 92019, Auckland 1142, New Zealand; 2 School of Medical Sciences, The University of Auckland, Private Bag 92019, Auckland 1142, New Zealand; 3 Membranes et Cibles Thérapeutiques (MCT), SSA, INSERM, Aix-Marseille Universite, 27 bd Jean Moulin, 13385 Marseille, France; 4 Laboratoire Molécules de Communication et Adaptation des Micro-organismes, UMR 7245 CNRS, Muséum National d’Histoire Naturelle, 57 rue Cuvier (C.P. 54), 75005 Paris, France

**Keywords:** antimicrobial activities, polyamine conjugates, antibiotic enhancement

## Abstract

As part of our search for new antimicrobials and antibiotic enhancers, a series of naphthyl- and biphenyl-substituted polyamine conjugates have been synthesized. The structurally-diverse library of compounds incorporated variation in the capping end groups and in the length of the polyamine (PA) core. Longer chain (PA-3-12-3) variants containing both 1-naphthyl and 2-naphthyl capping groups exhibited more pronounced intrinsic antimicrobial properties against methicillin-resistant *Staphylococcus aureus* (MRSA) (MIC ≤ 0.29 µM) and the fungus *Cryptococcus neoformans* (MIC ≤ 0.29 µM). Closer mechanistic study of one of these analogues, **20f**, identified it as a bactericide. In contrast to previously reported diarylacyl-substituted polyamines, several examples in the current set were able to enhance the antibiotic action of doxycycline and/or erythromycin towards the Gram-negative bacteria *Pseudomonas aeruginosa* and *Escherichia coli*. Two analogues (**19a** and **20c**) were of note, exhibiting greater than 32-fold enhancement in activity. This latter result suggests that α,ω-disubstituted polyamines bearing 1-naphthyl- and 2-naphthyl-capping groups are worthy of further investigation and optimization as non-toxic antibiotic enhancers.

## 1. Introduction

**Figure 1:**
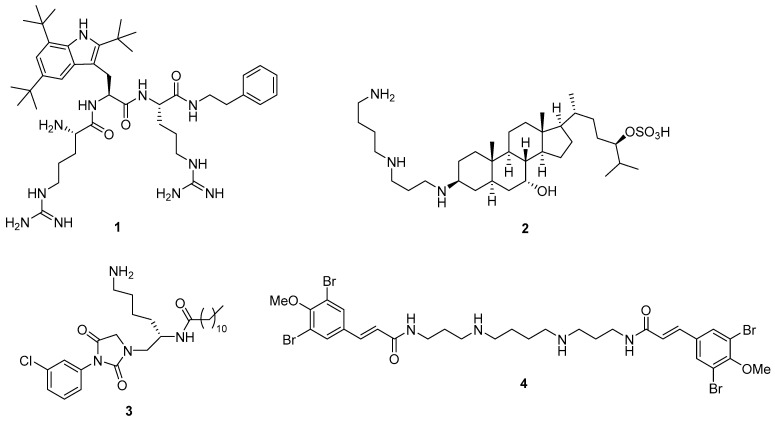
Structures of SMAMPs **1**–**4**.

Host-defense peptides (HDPs), produced by a wide variety of organisms in nature, including microorganisms, plants, invertebrates, and mammals, play essential roles as a first line of protection against viral, fungal, and bacterial infections [1,2,3,4,5,6]. A subset of HDPs are the antimicrobial peptides, small (50 amino acids or less) amphipathic peptides of diverse sequence homology and secondary structures [7]. From a structural perspective, antimicrobial peptides are characterized as containing hydrophobic residues on one side of the molecule and hydrophilic cationic residues on the other. These peptides are thought to act directly on bacterial cell membranes, with the cationic charges aiding electrostatic attraction to the negatively charged cell membrane, followed by hydrophobic residue insertion into the membrane leading to disruption, increased membrane permeability, and, ultimately, cell death [7]. Drug development issues associated with HDPs, including susceptibility to proteolysis, high production costs, and, in some case, low to moderate activity under physiological salt conditions, prevent their direct introduction as clinical agents [8]. In order to overcome these deficiencies, an extensive amount of research has been directed towards the synthesis and biological evaluation of synthetic mimics of antimicrobial peptides, so-called SMAMPs [9,10,11,12,13,14,15]. Numerous different structural classes of SMAMPs have been identified, including shorter peptides (e.g., LTX-109 (**1**)), sterols (e.g., squalamine (**2**)), and hydantoins (e.g., **3**) (Figure 1) [15,16,17]. Although they cover diverse chemo-types, all three of these examples are thought to act via a general bacterial membrane-targeting mechanism, with insertion leading to membrane disruption [16,18,19]. LTX-109 is one example of a number of different antimicrobial peptides that have entered clinical trials for the treatment of microbial infections [20].

The discovery of squalamine (**2**), a polyamine-containing aminosterol isolated from the dogfish shark *Squalus acanthias*, and observation of its broad-spectrum activity towards both Gram-positive and Gram-negative bacteria [21] prompted further investigation of the marine environment in the search for new classes of antimicrobials [22]. The marine sponge natural product ianthelliformisamine C (**4**) exhibits antimicrobial activity and can also enhance the activity of legacy antibiotics towards drug resistant Gram-negative bacteria [23,24,25]. Ianthelliformisamine C has all the structural attributes of an SMAMP, with the secondary amines of the polyamine fragment being protonated at physiological pH and the terminal cinnamate capping group being lipophilic, defining this as a scaffold worthy of attention [22,26].

**Figure 2:**
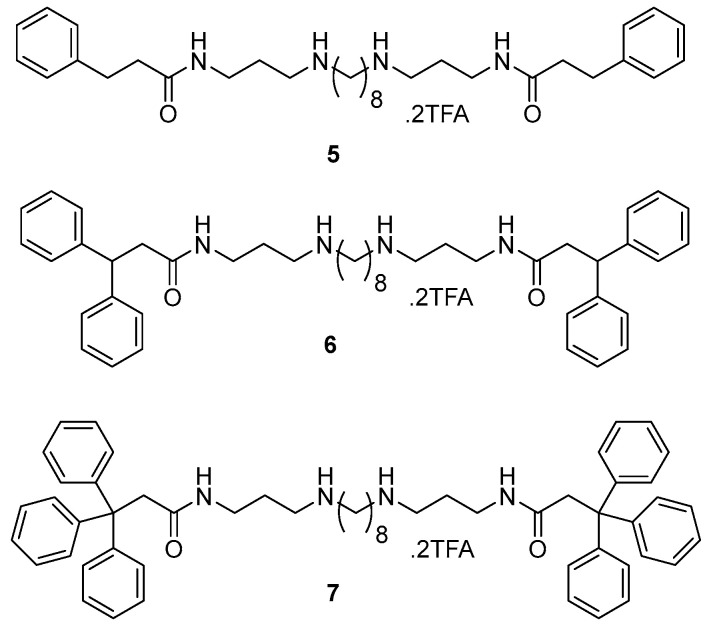
Structures of α,ω-diacylaryl substituted polyamine analogues **5**–**7** [27].

We recently reported the synthesis and antimicrobial evaluation of a set of α,ω-diacylaryl substituted polyamine analogues, of which examples **5–7** (Figure 2) are representative [27].

Analogues bearing a single aryl group at each end of a polyamine (PA) chain, e.g., **5**, were found to be almost uniformly inactive towards a panel of Gram-positive and Gram-negative bacteria and fungal strains, with only the longer polyamine chain variants (PA-3-10-3 and PA-3-12-3) exhibiting weak-to-potent activity towards methicillin-resistant *Staphylococcus aureus* (MRSA). Increasing the lipophilicity by inclusion of one or two additional phenyl rings in the capping acid created sets of analogues, e.g., **6** and **7**, that demonstrated good to excellent growth inhibition properties towards *Staphylococcus aureus* (MIC 3.13 µM for both), MRSA (MIC ≤ 0.28 µM and MIC ≤ 0.24 µM, respectively), and *Escherichia coli* (MIC 2.2 µM and MIC 7.6 µM, respectively). When counter screen cytotoxicity and hemolytic activities were combined with calculated LogP values, it became apparent that optimal selectivity for antimicrobial activity was observed for diaryl-containing capping groups with whole molecule cLogP in the range 7–8.5. Any analogues with cLogP greater than 9–10 appear to breach a ‘second hydrophobicity threshold’ [28], leading to greater disruption of mammalian membranes and inherent toxicity. Mechanism of action studies carried out on the diaryl-analogue **6** identified it as a strong disruptor of bacterial cell membranes and to be bactericidal [27], making it a good starting point for further optimization.

**Figure 3:**
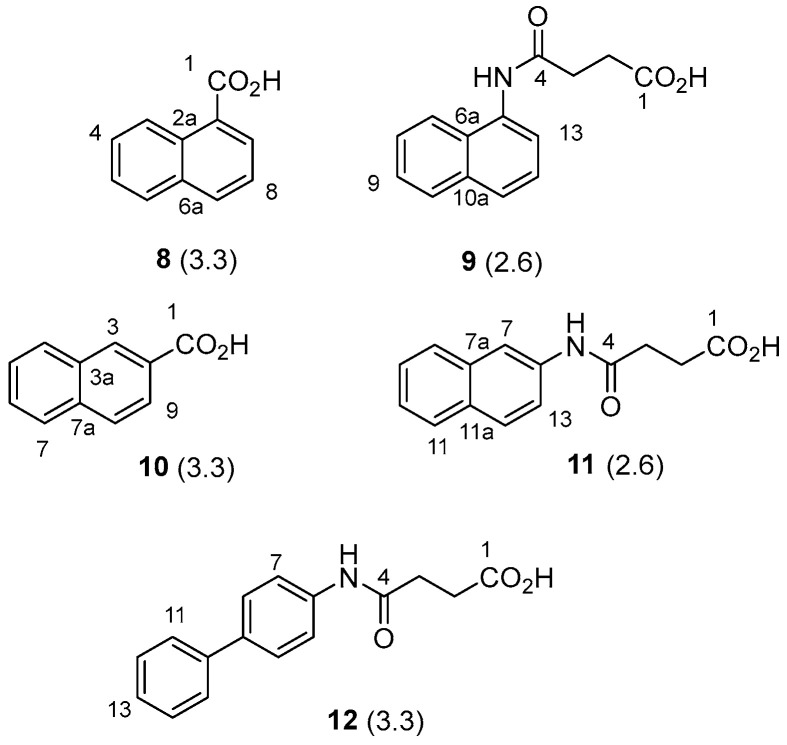
Structures of aromatic head groups **8**–**12** with cLogP values of the corresponding methyl ester in parentheses.

Mindful of not exceeding the second hydrophobicity threshold, we chose to prepare new examples of α,ω-disubstituted polyamines that targeted the cLogP range of approximately 5–9 and incorporated aryl-carboxylic acids **8**–**12** (Figure 3), which contained variations in shape, size, lipophilicity and position of attachment to the polyamine core.

Herein, we report the synthesis of a set of new α,ω-disubstituted polyamines that explore variation in the size and shape of the chain end-groups within a narrow band of lipophilicity as well as variation in polyamine length. All analogues were evaluated for antimicrobial activities against a set of Gram-positive and Gram-negative bacteria and two fungi strains, and for the ability to enhance the antibiotic action of doxycycline and erythromycin towards the Gram-negative bacteria *Pseudomonas aeruginosa* and *E. coli*, respectively.

## 2. Chemistry

**Scheme 1:**
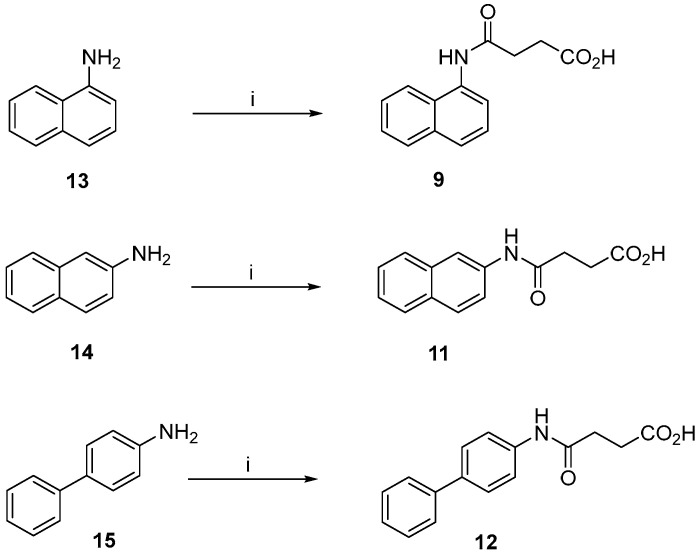
Synthesis of carboxylic acid derivatives **9**, **11**, and **12**. *Reagents and conditions*: (i) Arylamine (**13**–**15**) (1.0 equiv.), succinic anhydride (1.0 equiv.), CH_2_Cl_2_, r.t., N_2_, 9–24 h (yields: **9**, 97%; **11**, 76%; and **12**, 45%).

Of the five carboxylic acids required for this study (**8**–**12**), two (**8** and **10**) were commercially available. Carboxylic acids **9**, **11,** and **12** were prepared by reaction of naphthalen-1-amine (**13**), naphthalen-2-amine (**14**), and 4-aminobiphenyl (**15**) with succinic anhydride, in yields of 45–97% (Scheme 1, Supplementary Materials Figures S1–S3). 

**Figure 4:**
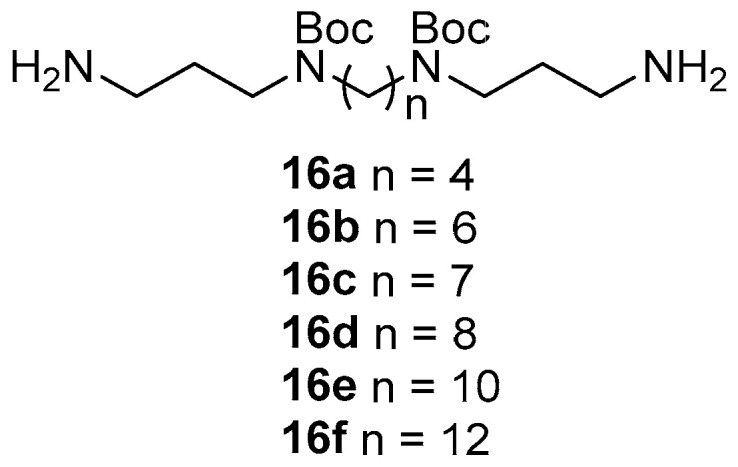
Boc-protected polyamine scaffolds **16a**–**f**.

Syntheses of the core Boc-protected polyamine scaffolds **16a**–**f** (Figure 4) have been previously described [29,30,31,32]. These six polyamines (PA3-4-3, PA3-6-3, PA3-7-3, PA3-8-3, PA3-10-3, PA3-12-3) contain variations in chain length, lipophilicity, and spatial separation of the dialkylammonium ion positive charges, all of which were considered to potentially have some influence on bioactivity.

Reaction of carboxylic acids **8**–**12** with Boc-protected polyamines **16a**–**f** utilized coupling reagents EDC·HCl or EDC·HCl/HOBt in anhydrous CH_2_Cl_2_, and then the products were deprotected using 2,2,2-trifluoroacetic acid (TFA) to create the target compounds as their di-TFA salts (Scheme 2, Figures S4–S32).

**Figure 5:**
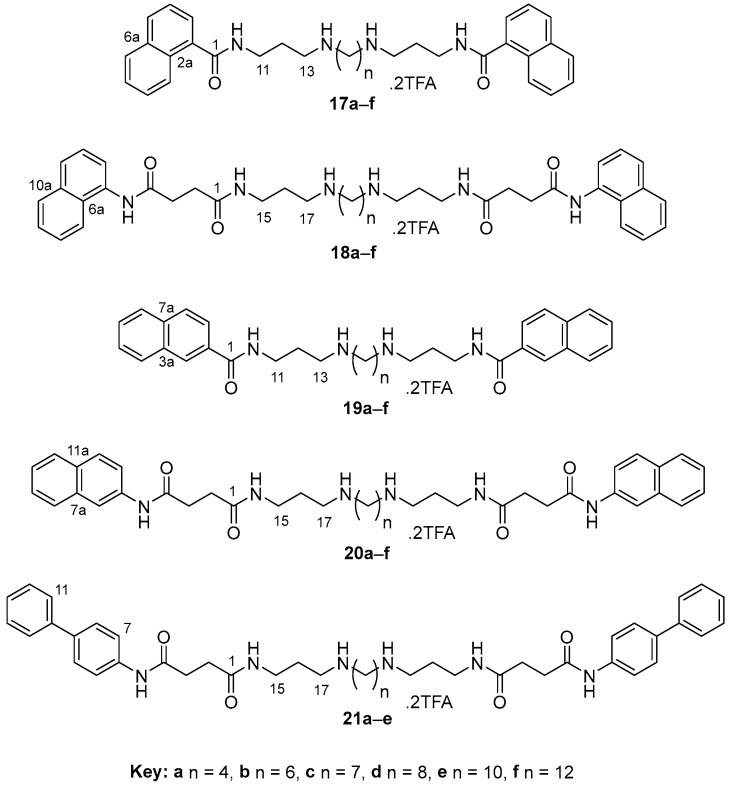
The structures of α,ω-disubstituted polyamines **17**–**21**.

The structures of the synthesized α,ω-disubstituted polyamine library are shown in Figure 5.

## 3. Results and Discussion

The antimicrobial activity of each compound was initially assessed against various bacterial strains, including *S. aureus* and MRSA, *P. aeruginosa, E. coli, Klebsiella pneumoniae*, and *Acinetobacter baumannii*, as well as fungal strains such as *Candida albicans* and *Cryptococcus neoformans* (Table 1). As a set, analogues containing a 1-naphthyl substituted end group (**17a**–**f**, **18a**–**f**) were predominantly inactive towards all microbes, with the exceptions being the longer polyamine chain variants **17e** (MRSA MIC 9.7 µM), **17f** (MRSA MIC 0.29 µM, *A. baumannii* MIC 0.29 µM, *C. neoformans* MIC 0.29 µM), and **18f** (*S. aureus* MIC 3.15 µM, MRSA MIC ≤ 0.25 µM, *C. neoformans* MIC ≤ 0.25 µM). In contrast, the 2-substituted naphthyl analogue sets **19a**–**f** and **20a**–**f** contained a greater number of active compounds, with five analogues (**19c**, **19e**, **19f**, **20c**, and **20f**) exhibiting activity towards primarily the Gram-positive bacteria *S. aureus* and/or MRSA and the fungus *C. neoformans*. Direct comparison of antimicrobial activities exhibited by corresponding 1-naphthyl vs. 2-naphthyl substituted analogues suggested, however, that there was little to no difference between the series of compounds as far as intrinsic antimicrobial activity was concerned. For example, analogues **17f** vs. **19f** were equipotent towards the same three microbial strains (MRSA MIC 0.29 µM, *A. baumannii* MIC 0.29 µM, *C. neoformans* MIC 0.29 µM), and **18f** vs. **20f** were essentially identical growth inhibitors towards *S. aureus* (MIC 3.125 µM), MRSA (MIC ≤ 0.25 µM), *E. coli* (MIC 6.3–12.5 µM), and *C. neoformans* (MIC ≤ 0.25 µM). The biphenyl substituted series of analogues **21a**–**e** were noticeably different in their spectrum of antimicrobial activities, with all analogues exhibiting activity towards just MRSA with MIC 0.25–2.1 µM.

The majority of the compounds, **17**–**21,** were evaluated for cytotoxicity towards human kidney epithelial cell line (HEK293) and for hemolytic activity towards human red blood cells (Table 2). Of the set of compounds tested, only biphenyl **21e** was considered to be hemolytic (HC_10_ 6.3 µM), and the observation of cytotoxicity was limited to the four analogues **17e** (IC_50_ 23.7 µM), 1**9a** (IC_50_ 26 µM), **19c** (IC_50_ 20.5 µM), and **19e** (IC_50_ 4.75 µM). The latter results, in particular, emphasize that cytotoxicity in the current series is not determined by lipophilicity alone, as other analogues with similar cLogP values (e.g., **17a**–**d, 18a**–**e, 20a**–**e**) were deemed non-toxic.

**Figure 6:**
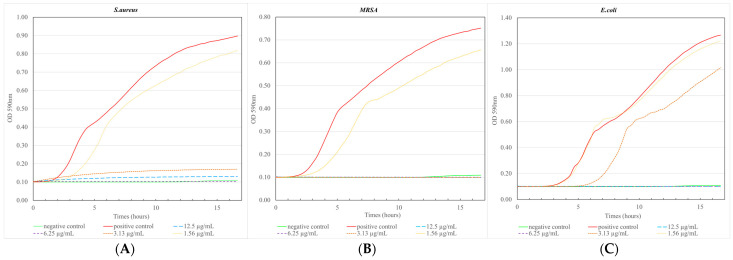
Bacterial growth inhibition exhibited by **20f** against (**A**) *S. aureus* (ATCC 25923); (**B**) MRSA (CF-Marseille); and (**C**) *E. coli* (ATCC 25922) with different concentrations. Positive control was bacteria only and negative control was media only.

To conduct a detailed analysis of the antibacterial activity and initial assessment of the mechanism of action, analogue **20f** was selected due to its strong antibacterial properties, and because it was devoid of any observed cytotoxic or hemolytic effects. To evaluate the kinetics of antibacterial activity, real-time growth inhibition curves were measured for two Gram-positive bacteria (*S. aureus* (ATCC 25923), MRSA (CF-Marseille) [34]) and the Gram-negative bacterium *E. coli* (ATCC 25922). The test compound completely inhibited the growth of the Gram-positive bacteria strains at 3.15 μM (3.13 µg/mL) or higher concentrations, with growth observed at the lowest test concentration of 1.57 µM (1.56 µg/mL) (Figure 6A,B). In the case of the Gram-negative bacterium *E. coli* (Figure 6C), bacterial growth inhibition was observed at test concentrations of 6.29 µM (6.25 µg/mL) or higher. MIC values of 3.15 μM (3.13 μg/mL), 3.15 µM (3.13 µg/mL), and 6.29 µM (6.25 µg/mL) were determined for analogue **20f** against *S. aureus* (ATCC 25923), MRSA (CF-Marseille), and *E. coli* (ATCC 25922), respectively. These values corresponded to the inhibitory concentrations observed at the 18-hour mark in the real-time growth inhibition curve plots. Additionally, the same values were observed for the minimum bactericidal concentration (MBC) of **20f** against all three organisms, indicating its bactericidal activity.

The ability of compounds **17**–**21** to enhance the antibiotic activity of doxycycline against *P. aeruginosa* (ATCC 27853) and of erythromycin against *E. coli* (ATCC 25922) were determined (Table 3). In the case of doxycycline, a low-dose fixed concentration of 2 µg/mL (4.5 µM) was used, being 20-fold lower than the intrinsic MIC (40 µg/mL (90 µM)) against *P. aeruginosa*. Each of the compounds were then evaluated at a range of concentrations, with the upper limit being dependent upon the intrinsic MIC towards *P. aeruginosa* (Table 1). Two examples of 1-naphthyl-substituted analogues (**17b** and **18c**) were identified as modest enhancers of the action of doxycycline, with MICs of 12.5 µM, representing 16-fold enhancements over their intrinsic MIC values. A further five examples of 2-naphthyl-substituted variants, **19a**, **19c**, **19d**, **20a**, **20c**, and **20f**, exhibited notable levels of enhancement, with MICs of 16.9, 4.0, 15.7, 28, 6.25, and 12.6 µM, respectively. When compared to their intrinsic growth inhibition activities towards *P. aeruginosa* (Table 1), these represented 40-fold, 8-fold, 8-fold, 20-fold, >32-fold, and >8-fold enhancements, respectively. The ability of the two structurally related 2-naphthyl-substituted PA-3-4-3 (spermine) analogues **19a** and **20a** to enhance the action of doxycycline towards *P. aeruginosa* was investigated more closely, revealing a dose-dependent response when the doxycycline concentration was varied from 2 to 8 µg/mL (Table 4).

Evaluation of compounds **17**–**21** to enhance the antibiotic activity of erythromycin, using a fixed low dose of 8 µg/mL (10.9 µM) against *E. coli* (ATCC 25922) identified three analogues in particular, **19a** (MIC 4.2 µM, 64-fold enhancement), **19d** (MIC 15.7 µM, 8-fold), and **20e** (MIC 13 µM, 8-fold enhancement), with strong levels of enhancement (MIC ≤ 20 µM). In addition, they were 8–10-fold more active in combination than when tested alone (Table 3).

**Figure 7:**
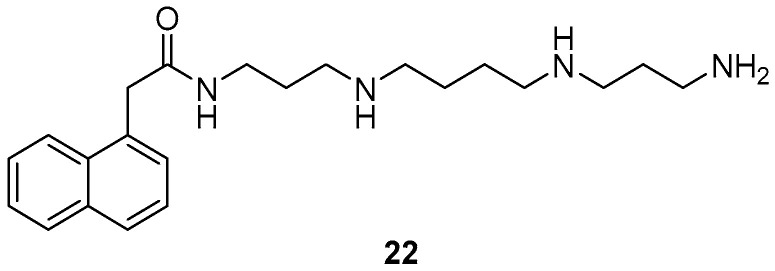
Structure of antibiotic enhancer naphthylacetylspermine (**22**).

Taken together, these studies have identified a number of predominantly 2-naphthyl-substituted polyamines as strong enhancers of the antibiotic action of doxycycline and/or erythromycin towards the Gram-negative bacteria *P. aeruginosa*, and *E. coli*. It is interesting to compare these results with our previous investigation of α,ω-diacylarylpolyamines, e.g., **6**, which revealed them to be active antimicrobials but with weak antibiotic enhancement properties (e.g., doxycycline vs. *P. aeruginosa*, 4-fold increase to an MIC of 12.5 µM) [27]. The current results lead us to conclude that substituted naphthyl-polyamines may be worthy of further optimization as antibiotic enhancers. It is pertinent to note that Yasuda et al. have previously reported that naphthylacetylspermine (**22**) (Figure 7), a synthetic analogue of joro spider toxin, renders *E. coli* sensitive to hydrophobic antibiotics, including novobiocin and erythromycin, albeit weakly, at doses of 64–128 µg/mL [35].

## 4. Materials and Methods

### 4.1. Chemistry General Methods

Infrared spectra were run as dry films on an ATR crystal and acquired with a Perkin-Elmer 100 Fourier Transform infrared spectrometer equipped with a Universal ATR Sampling Accessory. Mass spectra were acquired on a Bruker micrOTOF Q II mass spectrometer. Melting points were obtained on an Electrothermal melting point apparatus and are uncorrected. The ^1^H, ^13^C NMR, and 2D NMR spectra were recorded at 298 °K on a Bruker AVANCE AVIII 400 MHz spectrometer at 400.13 and 100.62 MHz, using standard pulse sequences. Proto-deutero solvent signals were used as internal references (DMSO-*d*_6_: δ_H_ 2.50, δ_C_ 39.52; CD_3_OD: δ_H_ 3.31, δ_C_ 49.00). For ^1^H NMR, the data are quoted as position (δ), relative integral, multiplicity (s = singlet, d = doublet, t = triplet, q = quartet, m = multiplet, br = broad), coupling constant (*J*, Hz), and assignment to the atom. Atom positional assignments were made using 2D-NMR data acquired using standard pulse sequences. The ^13^C NMR data are quoted as position (δ) and assignment to the atom. Flash column chromatography was carried out using Davisil silica gel (40–60 μm) or LiChroprep RP-8 (40–63 µm) solid support. Silica gel thin layer chromatography (TLC) was conducted on 0.2 mm thick plates of DC-plastikfolien Kieselgel 60 F_254_ (Merck). Reversed-phase TLC was carried out on 0.2 mm thick plates of DC-Kieselgel 60 RP-18 F_254_S (Merck). All solvents used were of analytical grade or better and/or purified according to standard procedures. Chemical reagents used were purchased from standard chemical suppliers and used as purchased. All samples were determined to be >95% purity. Protected polyamines di-*tert*-butyl butane-1,4-diylbis((3-aminopropyl)carbamate) (**16a**), di-*tert*-butyl hexane-1,6-diylbis((3-aminopropyl)carbamate) (**16b**), di-*tert*-butyl heptane-1,7-diylbis((3-aminopropyl)carbamate) (**16c**), di-*tert*-butyl octane-1,8-diylbis((3-aminopropyl)carbamate) (**16d**), di-*tert*-butyl decane-1,10-diylbis((3-aminopropyl)carbamate) (**16e**), and di-*tert*-butyl dodecane-1,12-diylbis((3-aminopropyl)carbamate) (**16f**) were synthesized using literature procedures [29,30,31,32].

#### 4.1.1. General Procedure A—Diamide Bond Formation

The appropriate Boc-protected polyamine **16a**–**f** (1 equiv.) was added to a solution of carboxylic acid (2.2 equiv.), EDC⋅HCl (2.6 equiv.), HOBt (2.6 equiv.) and DIPEA (4–6 equiv.), that was stirred in anhydrous CH_2_Cl_2_ (1.5 mL) at 0 °C for 30 min under N_2_. The mixture was allowed to come to room temperature and was stirred for a further 20 h under N_2_. The reaction mixture was poured into CH_2_Cl_2_ (20 mL) and washed with saturated NaHCO_3_ (2 × 30 mL) followed by H_2_O (2 × 30 mL), and it was then dried under reduced pressure and purified by silica gel flash column chromatography (0–20% MeOH/CH_2_Cl_2_) to create the desired products.

#### 4.1.2. General Procedure B—Diamide Bond Formation

The appropriate Boc-protected polyamine **16a**–**f** (1 equiv.) was added to a solution of carboxylic acid (2.5 equiv.) and EDC·HCl (2.8 equiv.) with DMAP (5 equiv.) stirred in anhydrous CH_2_Cl_2_ (1.5 mL) at 0 °C for 10 min under N_2_. The mixture was allowed to come to room temperature and stirred for a further 12 h under N_2_. The reaction mixture was poured into CH_2_Cl_2_ (20 mL) and washed with saturated NaHCO_3_ (2 × 30 mL) followed by H_2_O (2 × 30 mL), and it was then dried under reduced pressure and purified by silica gel flash column chromatography (0–20% MeOH/CH_2_Cl_2_) to create the desired products.

#### 4.1.3. General Procedure C—Boc Deprotection

A solution of *tert-*butyl-carbamate derivative in CH_2_Cl_2_ (2 mL) and TFA (0.2 mL) was stirred at room temperature under N_2_ for 2 h, and it was followed by solvent removal under reduced pressure. The crude product was purified using C_8_ reversed-phase flash column chromatography eluting with 0–100% MeOH/H_2_O (0.05% TFA) to create the corresponding polyamine conjugate as the TFA salt.

### 4.2. Synthesis of Compounds

#### 4.2.1. 4-(Naphthalen-1-ylamino)-4-oxobutanoic Acid (**9**)

Naphthalen-1-amine (200 mg, 1.40 mmol) and succinic anhydride (140 mg, 1.40 mmol) were stirred in anhydrous CH_2_Cl_2_ (10 mL) for 9 h under an N_2_ atmosphere. The solvent was then removed under reduced pressure, and the crude product was purified by C_8_ reversed-phase column chromatography (100% H_2_O to 100% MeOH) to create **9** as a pale pink solid (330 mg, 97%). R*_f_* = 0.43 (SiO_2_, CH_2_Cl_2_:10% MeOH); m.p. 165–167 °C; IR (ATR) *v*_max_ 3302, 2919, 1711, 1531, 1401, 1176, 915, 773 cm^−1^; ^1^H NMR (DMSO-*d_6_*, 400 MHz) δ 12.16 (1H, br s, OH), 9.94 (1H, br s, NH-5), 8.12–8.10 (1H, m, H-7), 7.94–7.91 (1H, m, H-10), 7.75 (1H, d, *J* = 7.8 Hz, H-11), 7.66 (1H, d, *J* = 7.8 Hz, H-13), 7.55–7.51 (2H, m, H-8, H-9), 7.47 (1H, dd, *J* = 7.8, 7.8 Hz, H-12), 2.75 (2H, t, *J* = 6.8 Hz, H_2_-2), 2.60 (2H, t, *J* = 6.8 Hz, H_2_-3); ^13^C NMR (DMSO-*d_6_*, 100 MHz) δ 173.9 (C-1), 170.8 (C-4), 133.7 (C-6, C-10a), 128.0 (C-10), 127.8 (C-6a), 125.9 (C-8/C-9), 125.7 (C-12), 125.5 (C-8/C-9), 125.1 (C-11), 122.9 (C-7), 121.6 (C-13), 30.7 (C-2), 29.1 (C-3); (+)-HRESIMS *m/z* 266.0783 [M+Na]^+^ (calcd for C_14_H_13_NNaO_3_, 266.0788).

#### 4.2.2. 4-(Naphthalen-2-ylamino)-4-oxobutanoic Acid (**11**)

Naphthalen-2-amine (200 mg, 1.40 mmol) and succinic anhydride (140 mg, 1.40 mmol) were stirred in anhydrous CH_2_Cl_2_ (10 mL) for 9 h under an N_2_ atmosphere. The solvent was then removed under reduced pressure, and the crude product was purified by C_8_ reversed-phase column chromatography (100% H_2_O to 100% MeOH) to create **11** as a pink solid (260 mg, 76%). R*_f_* = 0.19 (SiO_2_, CH_2_Cl_2_:10% MeOH); m.p. 170–172 °C; IR (ATR) *v*_max_ 3059, 1706, 1653, 1558, 1397, 1257, 824, 742 cm^−1^; ^1^H NMR (DMSO-*d_6_*, 400 MHz) δ 10.18 (1H, br s, NH-5), 8.30 (1H, s, H-7), 7.85–7.77 (3H, m, H-8, H-11, H-12), 7.57 (1H, dd, *J* = 8.9, 2.0 Hz, H-13), 7.47–7.43 (1H, m, H-9), 7.40–7.36 (1H, m, H-10), 2.62 (2H, t, *J* = 6.0 Hz, H_2_-2), 2.56 (2H, t, *J* = 6.0, H_2_-3); ^13^C NMR (DMSO-*d_6_*, 100 MHz) δ 173.9 (C-1), 170.4 (C-4), 136.9 (C-6), 133.5 (C-7a), 129.6 (C-11a), 128.3 (C-12), 127.4 (C-11), 127.2 (C-8), 126.3 (C-9), 124.4 (C-10), 119.8 (C-13), 114.8 (C-7), 31.2 (C-2), 29.0 (C-3); (+)-HRESIMS [M+Na]^+^ *m/z* 266.0784 (calcd for C_14_H_13_NNaO_3_, 266.0788).

#### 4.2.3. 4-([1,1’-Biphenyl]-4-ylamino)-4-oxobutanoic Acid (**12**)

4-Aminobiphenyl (500 mg, 2.95 mmol) and succinic anhydride (295 mg, 2.95 mmol) were stirred in anhydrous CH_2_Cl_2_ (11 mL) for 24 h under an N_2_ atmosphere. The solvent was removed under reduced pressure, and the was product purified by recrystallisation from EtOH to create **12** as light orange crystals (356 mg, 45%). R*_f_* = 0.17 (SiO_2_, CH_2_Cl_2_:10% MeOH); m.p. >230 °C; IR (ATR) *v*_max_ 3273, 3031, 2934, 2635, 1689, 1651, 1529, 1403, 1186, 832, 750, 685 cm^−1^; ^1^H NMR (DMSO-*d_6_*, 400 MHz) δ 12.13 (1H, br s, OH), 10.05 (1H, br s, NH-5), 7.70–7.65 (2H, m, H-7), 7.65–7.59 (4H, m, H-8, H-11), 7.46–7.41 (2H, m, H-12), 7.34–7.29 (1H, m, H-13), 2.61–2.57 (2H, m, H_2_-2/H_2_-3), 2.55–2.52 (2H, m, H_2_-2/H_2_-3); ^13^C NMR (DMSO-*d_6_*, 100 MHz) δ 173.8 (C-1), 170.1 (C-4), 139.7 (C-10), 138.8 (C-6), 134.6 (C-9), 128.9 (C-12), 127.0 (C-13), 126.9 (C-8/C-11), 126.2 (C-8/C-11), 119.3 (C-7), 31.1 (C-2/C-3), 28.8 (C-2/C-3); (+)-HRESIMS *m/z* 292.0950 [M+Na]^+^ (calcd for C_16_H_15_NNaO_3_, 292.0944).

#### 4.2.4. *N*^1^*,N*^4^-Bis(3-(1-naphthamido)propyl)butane-1,4-diaminium 2,2,2-trifluoroacetate (**17a**)

Following general procedure A, the reaction of 1-naphthoic acid (**8**) (94 mg, 0.55 mmol), di-*tert*-butyl butane-1,4-diylbis((3-aminopropyl)carbamate) (**16a**) (100 mg, 0.25 mmol), EDC·HCl (124 mg, 0.65 mmol), HOBt (87 mg, 0.64 mmol), and DIPEA (0.26 mL, 1.49 mmol) in CH_2_Cl_2_ (1.5 mL) created di-*tert*-butyl butane-1,4-diylbis((3-(1-naphthamido)propyl)carbamate) (42 mg, 24%) as a white solid. Following general procedure C, the reaction of a sub-sample of this material (22 mg, 0.031 mmol) in CH_2_Cl_2_ (2 mL) with TFA (0.2 mL) created the di-TFA salt **17a** as a white solid after purification (12 mg, 52%). R*_f_* = 0.48 (RP-18, MeOH:10% HCl, 7:3); m.p. 238–239 °C; IR (ATR) *v*_max_ 3307, 2839, 1646, 1451, 1203, 1116, 1015 cm^−1^; ^1^H NMR (DMSO-*d_6_*, 400 MHz) δ 8.70–8.65 (4H, m, NH_2_-14), 8.21–8.18 (2H, m, H-3), 8.04–7.97 (4H, m, H-6, H-7), 7.62 (2H, dd, *J* = 7.0, 1.0 Hz, H-9), 7.59–7.53 (6H, m, H-4, H-5, H-8), 3.43–3.39 (4H, m, H_2_-11), 3.03–2.97 (8H, m, H_2_-13, H_2_-15), 1.96–1.89 (4H, m, H_2_-12), 1.69–1.64 (4H, m, H_2_-16); ^13^C NMR (DMSO-*d_6_*, 100 MHz) δ 168.9 (C-1), 134.5 (C-6a), 133.1 (C-2), 129.9 (C-7), 129.7 (C-2a), 128.2 (C-6), 126.7 (C-4), 126.2 (C-5), 125.3 (C-3/C-8), 125.2 (C-3/C-8), 124.9 (C-9), 46.1 (C-15), 44.8 (C-13), 36.3 (C-11), 26.0 (C-12), 22.7 (C-16); (+)-HRESIMS *m/z* 511.3068 [M+H]^+^ (calcd for C_32_H_39_N_4_O_2_, 511.3068).

#### 4.2.5. *N*^1^*,N*^6^*-*Bis(3-(1-naphthamido)propyl)hexane-1,6-diaminium 2,2,2-trifluoroacetate (**17b**)

Following general procedure A, the reaction of 1-naphthoic acid (**8**) (44.0 mg, 0.256 mmol), di-*tert*-butyl hexane-1,6-diylbis((3-aminopropyl)carbamate) (**16b**) (50 mg, 0.12 mmol), EDC·HCl (57.9 mg, 0.302 mmol), HOBt (40.8 mg, 0.30 mmol), and DIPEA (0.12 mL, 0.69 mmol) in CH_2_Cl_2_ (1.5 mL) created di-*tert*-butyl hexane-1,6-diylbis((3-(1-naphthamido)propyl)carbamate) as a colorless oil (27 mg, 30%). Following general procedure C, the reaction of a sub-sample of this material (18 mg, 0.024 mmol) in CH_2_Cl_2_ (2 mL) with TFA (0.2 mL) created the di-TFA salt **17b** as a white solid (16 mg, 87%) with no further purification required. R*_f_* = 0.47 (RP-18, MeOH:10% HCl, 7:3); m.p. 225–227 °C; IR (ATR) *v*_max_ 3293, 3057, 2944, 2856, 1675, 1542, 1313, 1201, 1132, 786, 721cm^−1^; ^1^H NMR (CD_3_OD, 400 MHz) δ 8.24–8.20 (2H, m, H-3), 8.03 (2H, d, *J* = 8.2 Hz, H-7), 7.98–7.94 (2H, m, H-6), 7.68 (2H, dd, *J* = 7.1, 1.4 Hz, H-9), 7.62–7.53 (6H, m, H-4, H-5, H-8), 3.59 (4H, t, *J* = 6.6 Hz, H_2_-11), 3.15 (4H, t, *J* = 7.4 Hz, H_2_-13), 3.07 (4H, t, *J* = 7.5 Hz, H_2_-15), 2.11–2.03 (4H, m, H_2_-12), 1.81–1.72 (4H, m, H_2_-16), 1.53–1.48 (4H, m, H_2_-17); ^13^C NMR (CD_3_OD, 100 MHz) δ 173.4 (C-1), 135.2 (C-2/C-6a), 135.0 (C-2/C-6a), 131.9 (C-7), 131.4 (C-2a), 129.6 (C-6), 128.1 (C-4), 127.6 (C-5), 126.6 (C-9), 126.1 (C-3), 126.0 (C-8), 48.9 (C-15), 46.6 (C-13), 37.5 (C-11), 27.8 (C-12), 27.1 (C-17), 27.0 (C-16); (+)-HRESIMS *m/z* 539.3385 [M+H]^+^ (calcd for C_34_H_43_N_4_O_2_, 539.3381).

#### 4.2.6. *N*^1^*,N*^7^-Bis(3-(1-naphthamido)propyl)heptane-1,7-diaminium 2,2,2-trifluoroacetate (**17c**)

Following general procedure A, the reaction of 1-naphthoic acid (**8**) (43.0 mg, 0.250 mmol), di-*tert*-butyl heptane-1,7-diylbis((3-aminopropyl)carbamate) (**16c**) (50 mg, 0.11 mmol), EDC·HCl (56.1 mg, 0.293 mmol), HOBt (39.6 mg, 0.29 mmol), and DIPEA (0.118 mL, 0.677 mmol) in CH_2_Cl_2_ (1.5 mL) created di-*tert*-butyl heptane-1,7-diylbis((3-aminopropyl)carbamate) as a colorless oil (45 mg, 54%). Following general procedure C, the reaction of a sub-sample of this material (6 mg, 0.008 mmol) in CH_2_Cl_2_ (2 mL) with TFA (0.2 mL) created the di-TFA salt **17c** as a colorless gum after purification (5 mg, 81%). R*_f_* = 0.63 (RP-18, MeOH:10% HCl, 7:3); IR (ATR) *v*_max_ 3752, 326, 2927, 2857, 2305, 1678, 1665, 1543, 1199, 1136, 806, 723 cm^−1^; ^1^H NMR (CD_3_OD, 400 MHz) δ 8.12–8.09 (2H, m, H-3), 7.90 (2H, d, *J* = 8.5 Hz, H-7), 7.85–7.82 (2H, m, H-6), 7.55 (2H, dd, *J* = 7.2, 1.4 Hz, H-9), 7.49–7.40 (6H, m, H-4, H-5, H-8), 3.47 (4H, t, *J* = 6.6 Hz, H_2_-11), 3.03 (4H, t, *J* = 7.4 Hz, H_2_-13), 2.94 (4H, t, *J* = 7.7 Hz, H_2_-15), 1.98–1.90 (4H, m, H_2_-12), 1.67–1.59 (4H, m, H_2_-16), 1.38–1.33 (6H, m, H_2_-17, H_2_-18); ^13^C NMR (CD_3_OD, 100 MHz) δ 173.5 (C-1), 135.2 (C-2/C-6a), 134.9 (C-2/C-6a), 131.9 (C-7), 131.4 (C-2a), 129.6 (C-6), 128.1 (C-4), 127.5 (C-5), 126.6 (C-9), 126.1 (C-3), 125.9 (C-8), 49.0 (C-15), 46.5 (C-13), 37.5 (C-11), 29.6 (C-18), 27.8 (C-12), 27.3 (C-16/C-17); (+)-HRESIMS *m/z* 553.3534 [M+H]^+^ (calcd for C_35_H_45_N_4_O_2_, 553.3537).

#### 4.2.7. *N*^1^*,N*^8^-Bis(3-(1-naphthamido)propyl)octane-1,8-diaminium 2,2,2-trifluoroacetate (**17d**)

Following general procedure A, the reaction of 1-naphthoic acid (**8**) (41.3 mg, 0.240 mmol), di-*tert*-butyl octane-1,8-diylbis((3-aminopropyl)carbamate) (**16d**) (50 mg, 0.11 mmol), EDC·HCl (54.3 mg, 0.283 mmol), HOBt (38.3 mg, 0.28 mmol), and DIPEA (0.114 mL, 0.653 mmol) in CH_2_Cl_2_ (1.5 mL) created di-*tert*-butyl octane-1,8-diylbis((3-aminopropyl)carbamate) as a colorless oil (37 mg, 44%). Following general procedure C, the reaction of a sub-sample of this material (13 mg, 0.017 mmol) in CH_2_Cl_2_ (2 mL) with TFA (0.2 mL) created the di-TFA salt **17d** as a colorless gum after purification (4 mg, 30%). R*_f_* = 0.63 (RP-18, MeOH:10% HCl, 7:3); IR (ATR) *v*_max_ 3743, 3274, 3057, 2935, 2861, 1675, 1542, 1468, 1200, 1132, 784, 721 cm^−1^; ^1^H NMR (CD_3_OD, 400 MHz) δ 8.26–8.21 (2H, m, H-3), 8.03 (2H, d, *J* = 8.6 Hz, H-7), 7.98–7.94 (2H, m, H-6), 7.68 (2H, dd, *J* = 6.9, 1.2 Hz, H-9), 7.62–7.52 (6H, m, H-4, H-5, H-8), 3.60 (4H, t, *J* = 6.7 Hz, H_2_-11), 3.17 (4H, t, *J* = 7.5 Hz, H_2_-13), 3.06 (4H, t, *J* = 7.8 Hz, H_2_-15), 2.12–2.03 (4H, m, H_2_-12), 1.78–1.70 (4H, m, H_2_-16), 1.50–1.39 (8H, m, H_2_-17, H_2_-18); ^13^C NMR (CD_3_OD, 100 MHz) δ 173.5 (C-1), 135.2 (C-6a), 135.0 (C-2), 132.0 (C-7), 131.4 (C-2a), 129.6 (C-6), 128.1 (C-4), 127.6 (C-5), 126.6 (C-9), 126.1 (C-3), 126.0 (C-8), 49.0 (C-15), 46.6 (C-13), 37.5 (C-11), 30.0 (C-18), 27.8 (C-12), 27.4 (C-16/C-17), 27.3 (C-16/C-17); (+)-HRESIMS *m/z* 567.3680 [M+H]^+^ (calcd for C_36_H_47_N_4_O_2_, 567.3694).

#### 4.2.8. *N*^1^*,N*^10^-Bis(3-(1-naphthamido)propyl)decane-1,10-diaminium 2,2,2-trifluoroacetate (**17e**)

Following general procedure A, the reaction of 1-napthoic acid (**8**) (78 mg, 0.45 mmol), di-*tert*-butyl decane-1,10-diylbis((3-aminopropyl)carbamate) (**16e**) (100 mg, 0.21 mmol), EDC·HCl (103 mg, 0.54 mmol), HOBt (72 mg, 0.53 mmol), and DIPEA (0.22 mL, 1.26 mmol) in CH_2_Cl_2_ (1.5 mL) created di-*tert*-butyl decane-1,10-diylbis((3-(1-naphthamido)propyl)carbamate) (120 mg, 72%) as a colorless oil. Following general procedure C, the reaction of a sub-sample of this material (99 mg, 0.13 mmol) in CH_2_Cl_2_ (2 mL) with TFA (0.2 mL) created the di-TFA salt **17e** (93 mg, 87%) as a yellow oil with no further purification required. R*_f_* = 0.27 (RP-18, MeOH:10% HCl, 7:3); IR (ATR) *v*_max_ 3306, 2944, 2832, 1685, 1448, 1113, 1022 cm^−1^; ^1^H NMR (CD_3_OD, 400 MHz) δ 8.23–8.20 (2H, m, H-3), 8.00 (2H, d, *J* = 8.3 Hz, H-7), 7.99–7.91 (2H, m, H-6), 7.65 (2H, dd, *J* = 7.0, 0.7 Hz, H-9), 7.58–7.50 (6H, m, H-4, H-5, H-8), 3.58 (4H, t, *J* = 6.6 Hz, H_2_-11), 3.14 (4H, t, *J* = 7.4 Hz, H_2_-13), 3.03 (4H, t, *J* = 7.8 Hz, H_2_-15), 2.09–2.02 (4H, m, H_2_-12), 1.75–1.67 (4H, m, H_2_-16), 1.42–1.33 (12H, m, H_2_-17, H_2_-18, H_2_-19); ^13^C NMR (CD_3_OD, 100 MHz) δ 173.5 (C-1), 135.2 (C-2/C-6a), 135.0 (C-2/C-6a), 131.9 (C-7), 131.4 (C-2a), 129.6 (C-6), 128.1 (C-4), 127.6 (C-5), 126.6 (C-9), 126.1 (C-3), 126.0 (C-8), 49.1 (C-15), 46.6 (C-13), 37.6 (C-11), 30.4 (C-18/C-19), 30.2 (C-18/C-19), 27.8 (C-12), 27.5 (C-17), 27.4 (C-16); (+)-HRESIMS *m/z* 595.3998 [M+H]^+^ (calcd for C_38_H_51_N_4_O_2_, 595.4007).

#### 4.2.9. *N*^1^*,N*^12^-Bis(3-(1-naphthamido)propyl)dodecane-1,12-diaminium 2,2,2-trifluoroacetate (**17f**)

Following general procedure A, the reaction of 1-naphthoic acid (**8**) (52 mg, 0.30 mmol), di-*tert*-butyl dodecane-1,12-diylbis((3-aminopropyl)carbamate) (**16f**) (71 mg, 0.14 mmol), EDC·HCl (69 mg, 0.36 mmol), HOBt (47 mg, 0.35 mmol), and DIPEA (0.14 mL, 0.80 mmol) in CH_2_Cl_2_ (1.5 mL) created di-*tert*-butyl dodecane-1,12-diylbis((3-(1-naphthamido)propyl)carbamate) (43 mg, 37%) as a white wax. Following general procedure C, the reaction of a sub-sample of this material (19 mg, 0.023 mmol) in CH_2_Cl_2_ (2 mL) with TFA (0.2 mL) created the di-TFA salt **17f** (18 mg, 92%) as a white wax with no further purification required. R*_f_* = 0.15 (RP-18, MeOH:10% HCl, 7:3); IR (ATR) *v*_max_ 3326, 2944, 2834, 1654, 1450, 1246, 1116, 1022 cm^−1^; ^1^H NMR (CD_3_OD, 400 MHz) δ 8.23–8.20 (2H, m, H-3), 8.00 (2H, d, *J* = 8.2 Hz, H-7), 7.95–7.92 (2H, m, H-6), 7.66 (2H, dd, *J* = 7.0, 1.0 Hz, H-9), 7.59–7.50 (6H, m, H-4, H-5, H-8), 3.58 (4H, t, *J* = 6.6 Hz, H_2_-11), 3.15 (4H, t, *J* = 7.4 Hz, H_2_-13), 3.04 (4H, t, *J* = 7.8 Hz, H_2_-15), 2.09–2.02 (4H, m, H_2_-12), 1.75–1.68 (4H, m, H_2_-16), 1.43–1.30 (16H, m, H_2_-17, H_2_-18, H_2_-19, H_2_-20); ^13^C NMR (CD_3_OD, 100 MHz) δ 173.4 (C-1), 135.2 (C-2/C-6a), 135.0 (C-2/C-6a), 131.9 (C-7), 131.4 (C-2a), 129.6 (C-6), 128.1 (C-4), 127.5 (C-5), 126.6 (C-9), 126.1 (C-3), 125.9 (C-8), 49.1 (C-15), 46.6 (C-13), 37.6 (C-11), 30.6 (C-18/C-19/C-20), 30.5 (C-18/C-19/C-20), 30.2 (C-18/C-19/C-20), 27.8 (C-12), 27.5 (C-17), 27.4 (C-16); (+)-HRESIMS *m/z* 623.4321 [M+H]^+^ (calcd for C_40_H_55_N_4_O_2_, 623.4320).

#### 4.2.10. *N*^1^,*N*^4^-Bis(3-(4-(naphthalen-1-ylamino)-4-oxobutanamido)propyl)butane-1,4-diaminium 2,2,2-trifluoroacetate (**18a**)

Following general procedure B, the reaction of carboxylic acid **9** (76 mg, 0.31 mmol), di-*tert*-butyl butane-1,4-diylbis((3-aminopropyl)carbamate) (**16a**) (50 mg, 0.12 mmol), EDC·HCl (67 mg, 0.35 mmol), and DMAP (76 mg, 0.62 mmol) in CH_2_Cl_2_ (1.5 mL) created di-*tert*-butyl butane-1,4-diylbis((3-(4-(naphthalen-1-ylamino)-4-oxobutanamido)propyl)carbamate) as a colorless oil (54 mg, 53%). Following general procedure C, the reaction of a sub-sample of this material (26 mg, 0.030 mmol) in CH_2_Cl_2_ (2 mL) with TFA (0.2 mL) created the di-TFA salt **18a** as a colorless oil after purification (22 mg, 83%). R*_f_* = 0.47 (RP-18, MeOH:10% HCl, 7:3); IR (ATR) *v*_max_ 3278, 1676, 1202, 1160, 799, 766 cm^−1^; ^1^H NMR (CD_3_OD, 400 MHz) δ 8.04 (2H, dd, *J* = 9.0, 2.0 Hz, H-10), 7.89 (2H, dd, *J* = 7.0, 2.0 Hz, H-7), 7.76 (2H, d, *J* = 8.5, Hz, H-13), 7.59–7.57 (2H, m, H-11), 7.56–7.50 (4H, m, H-8, H-9), 7.46 (2H, t, *J* = 7.8 Hz, H-12), 3.32–3.28 (4H, m, H_2_-15), 2.92–2.89 (4H, m, H_2_-2), 2.83 (4H, t, *J* = 7.0 Hz, H_2_-17), 2.65–2.62 (4H, m, H_2_-3), 2.57–2.54 (4H, m, H_2_-19), 1.82–1.75 (4H, m, H_2_-16), 1.36–1.32 (4H, m, H_2_-20); ^13^C NMR (CD_3_OD, 100 MHz) δ 176.3 (C-1), 174.3 (C-4), 135.7 (C-6), 134.3 (C-10a), 130.2 (C-6a), 129.4 (C-7), 127.6 (C-13), 127.4 (C-8/C-9), 127.3 (C-8/C-9), 126.6 (C-12), 124.0 (C-11), 123.6 (C-10), 47.8 (C-19), 46.0 (C-17), 36.6 (C-15), 32.0 (C-2), 31.6 (C-3), 27.7 (C-16), 23.9 (C-20); (+)-HRESIMS *m/z* 653.3807 [M+H]^+^ (calcd for C_38_H_49_N_6_O_4_, 653.3810).

#### 4.2.11. *N*^1^*,N*^6^-Bis(3-(4-(naphthalen-1-ylamino)-4-oxobutanamido)propyl)hexane-1,6-diaminium 2,2,2-trifluoroacetate (**18b**)

Following general procedure B, the reaction of carboxylic acid **9** (71 mg, 0.29 mmol), di-*tert*-butyl hexane-1,6-diylbis((3-aminopropyl)carbamate) (**16b**) (50 mg, 0.12 mmol), EDC·HCl (62 mg, 0.32 mmol), and DMAP (71 mg, 0.58 mmol) in CH_2_Cl_2_ (1.5 mL) created di-*tert*-butyl hexane-1,6-diylbis((3-(4-(naphthalen-1-ylamino)-4-oxobutanamido)propyl)carbamate) as a colorless oil (97 mg, 92%). Following general procedure C, the reaction of a sub-sample of this material (44 mg, 0.050 mmol) in CH_2_Cl_2_ (2 mL) with TFA (0.2 mL) created the di-TFA salt **18b** as an orange oil after purification (39 mg, 86%). R*_f_* = 0.47 (RP-18, MeOH:10% HCl, 7:3); IR (ATR) *v*_max_ 3279, 3050, 1671, 1542, 1201, 1132, 800, 777 cm^−1^; ^1^H NMR (CD_3_OD, 400 MHz) δ 8.01 (2H, dd, *J* = 9.0, 2.5 Hz, H-10), 7.85 (2H, dd, *J* = 7.5, 3.5 Hz, H-7), 7.71 (2H, d, *J* = 8.5, Hz, H-13), 7.57 (2H, dd, *J* = 7.5, 1.0 Hz, H-11), 7.51–7.47 (4H, m, H-8, H-9), 7.42 (2H, t, *J* = 7.8 Hz, H-12), 3.31–3.26 (4H, m, H_2_-15), 2.88 (8H, t, *J* = 6.5 Hz, H_2_-2, H_2_-17), 2.61–2.53 (8H, m, H_2_-3, H_2_-19), 1.82–1.75 (4H, m, H_2_-16), 1.33–1.26 (4H, m, H_2_-20), 0.91–0.87 (4H, m, H_2_-21); ^13^C NMR (CD_3_OD, 100 MHz) δ 176.3 (C-1), 174.2 (C-4), 135.7 (C-6), 134.4 (C-10a), 130.0 (C-6a), 129.4 (C-7), 127.5 (C-13), 127.33 (C-8/C-9), 127.27 (C-8/C-9), 126.5 (C-12), 123.7 (C-11), 123.6 (C-10), 48.7 (C-19), 46.0 (C-17), 36.5 (C-15), 32.0 (C-2), 31.5 (C-3), 27.8 (C-16), 26.8 (C-20/C-21), 26.7 (C-20/C-21); (+)-HRESIMS [M+H]^+^ *m/z* 681.4104 (calcd for C_40_H_53_N_6_O_4_, 681.4123).

#### 4.2.12. *N*^1^*,N*^7^-Bis(3-(4-(naphthalen-1-ylamino)-4-oxobutanamido)propyl)heptane-1,7-diaminium 2,2,2-trifluoroacetate (**18c**)

Following general procedure B, the reaction of carboxylic acid **9** (68 mg, 0.29 mmol), di-*tert*-butyl heptane-1,7-diylbis((3-aminopropyl)carbamate) (**16c**) (50 mg, 0.11 mmol), EDC·HCl (60 mg, 0.31 mmol), and DMAP (69 mg, 0.57 mmol) in CH_2_Cl_2_ (1.5 mL) created di-*tert*-butyl heptane-1,7-diylbis((3-(4-(naphthalen-1-ylamino)-4-oxobutanamido)propyl)carbamate) as a colorless oil (94 mg, 95%). Following general procedure C, the reaction of a sub-sample of this material (82 mg, 0.092 mmol) in CH_2_Cl_2_ (2 mL) with TFA (0.2 mL) created the di-TFA salt **18c** as a colorless oil after purification (59 mg, 69%). R*_f_* = 0.42 (RP-18, MeOH:10% HCl, 7:3); IR (ATR) *v*_max_ 3279, 3053, 1671, 1537, 1201, 1131, 800, 777 cm^−1^; ^1^H NMR (CD_3_OD, 400 MHz) δ 8.05 (2H, dd, *J* = 9.5, 1.5 Hz, H-10), 7.87 (2H, dd, *J* = 7.5, 2.5 Hz, H-7), 7.74 (2H, d, 8.0 Hz, H-13), 7.61 (2H, dd, *J* = 7.5, 1.0 Hz, H-11), 7.55–7.48 (4H, m, H-8, H-9), 7.45 (2H, t, *J* = 8.0 Hz, H-12), 3.33–3.30 (4H, m, H_2_-15), 2.91 (8H, t, *J* = 6.5 Hz, H_2_-2, H_2_-17), 2.65–2.61 (8H, m, H_2_-3, H_2_-19), 1.85–1.79 (4H, m, H_2_-16), 1.42–1.35 (4H, m, H_2_-20), 1.01–0.98 (6H, m, H_2_-21, H_2_-22); ^13^C NMR (CD_3_OD, 100 MHz) δ 176.2 (C-1), 174.1 (C-4), 135.7 (C-6), 134.3 (C-10a), 130.0 (C-6a), 129.4 (C-7), 127.5 (C-13), 127.3 (C-8/C-9), 127.2 (C-8/C-9), 126.5 (C-12), 123.7 (C-11), 123.6 (C-10), 48.8 (C-19), 46.0 (C-17), 36.6 (C-15), 32.0 (C-2), 31.6 (C-3), 29.4 (C-22), 27.7 (C-16), 27.0 (C-20/C-21), 26.9 (C-20/C-21); (+)-HRESIMS [M+H]^+^ *m/z* 695.4265 (calcd for C_41_H_55_N_6_O_4_, 695.4279).

#### 4.2.13. *N*^1^*,N*^8^-Bis(3-(4-(naphthalen-1-ylamino)-4-oxobutanamido)propyl)octane-1,8-diaminium 2,2,2-trifluoroacetate (**18d**)

Following general procedure B, the reaction of carboxylic acid **9** (67 mg, 0.28 mmol), di-*tert*-butyl octane-1,8-diylbis((3-aminopropyl)carbamate) (**16d**) (50 mg, 0.11 mmol), EDC·HCl (59 mg, 0.31 mmol), and DMAP (67 mg, 0.55 mmol) in CH_2_Cl_2_ (1.5 mL) created di-*tert*-butyl octane-1,8-diylbis((3-(4-(naphthalen-1-ylamino)-4-oxobutanamido)propyl)carbamate) as a colorless oil (69 mg, 69%). Following general procedure C, the reaction of this material (69 mg, 0.076 mmol) in CH_2_Cl_2_ (2 mL) with TFA (0.2 mL) created the di-TFA salt **18d** as a colorless oil after purification (37 mg, 54%). R*_f_* = 0.40 (RP-18, MeOH:10% HCl, 7:3); IR (ATR) *v*_max_ 3052, 1655, 1542, 1201, 1132, 799, 777 cm^−1^; ^1^H NMR (CD_3_OD, 400 MHz) δ 8.05 (2H, dd, *J* = 8.0, 1.5 Hz, H-10), 7.88 (2H, dd, *J* = 7.5, 2.0 Hz, H-7), 7.75 (2H, d, *J* = 8.0 Hz, H-13), 7.62 (2H, dd, *J* = 7.5, 1.0 Hz, H-11), 7.56–7.49 (4H, m, H-8, H-9), 7.46 (2H, t, *J* = 8.0 Hz, H-12), 3.34–3.31 (4H, m, H_2_-15), 2.95–2.91 (8H, m, H_2_-2, H_2_-17), 2.67–2.62 (8H, m, H_2_-3, H_2_-19), 1.86–1.80 (4H, m, H_2_-16), 1.45–1.39 (4H, m, H_2_-20), 1.07–1.01 (8H, m, H_2_-21, H_2_-22); ^13^C NMR (CD_3_OD, 100 MHz) δ 176.2 (C-1), 174.1 (C-4), 135.7 (C-6), 134.3 (C-10a), 130.0 (C-6a), 129.4 (C-7), 127.4 (C-13), 127.3 (C-8/C-9), 127.2 (C-8/C-9), 126.5 (C-12), 123.6 (C-11), 123.5 (C-10), 48.9 (C-19), 46.0 (C-17), 36.6 (C-15), 32.0 (C-2), 31.6 (C-3), 29.8 (C-22), 27.8 (C-16), 27.2 (C-20/C-21), 27.1 (C-20/C-21); (+)-HRESIMS [M+2H]^2+^ *m/z* 355.2250 (calcd for C_42_H_58_N_6_O_4_, 355.2254).

#### 4.2.14. *N*^1^*,N*^10^-Bis(3-(4-(naphthalen-1-ylamino)-4-oxobutanamido)propyl)decane-1,10-diaminium 2,2,2-trifluoroacetate (**18e**)

Following general procedure B, the reaction of carboxylic acid **9** (63 mg, 0.26 mmol), di-*tert*-butyl decane-1,10-diylbis((3-aminopropyl)carbamate) (**16e**) (50 mg, 0.10 mmol), EDC·HCl (55 mg, 0.29 mmol), and DMAP (63 mg, 0.52 mmol) in CH_2_Cl_2_ (1.5 mL) created di-*tert*-butyl decane-1,10-diylbis((3-(4-(naphthalen-1-ylamino)-4-oxobutanamido)propyl)carbamate) as a colorless oil (67 mg, 72%). Following general procedure C, the reaction of a sub-sample of this material (27 mg, 0.029 mmol) in CH_2_Cl_2_ (2 mL) with TFA (0.2 mL) created the di-TFA salt **18e** as a colorless oil after purification (26 mg, 93%). R*_f_* = 0.23 (RP-18, MeOH: 10% HCl, 7:3); IR (ATR) *v*_max_ 2937, 1673, 1538, 1202, 1133, 800, 776, 721 cm^−1^; ^1^H NMR (CD_3_OD, 400 MHz) δ 8.05 (2H, dd, *J* = 8.0, 1.5 Hz, H-10), 7.88 (2H, dd, *J* = 7.4, 2.4 Hz, H-7), 7.76 (2H, d, *J* = 8.5, Hz, H-13), 7.62 (2H, d, *J* = 7.5 Hz, H-11), 7.56–7.50 (4H, m, H-8, H-9), 7.46 (2H, t, *J* = 8.0 Hz, H-12), 3.34–3.30 (4H, m, H_2_-15), 2.95–2.91 (8H, m, H_2_-2, H_2_-17), 2.68–2.61 (8H, m, H_2_-3, H_2_-19), 1.86–1.79 (4H, m, H_2_-16), 1.47–1.40 (4H, m, H_2_-20), 1.14–1.08 (12H, m, H_2_-21, H_2_-22, H_2_-23); ^13^C NMR (CD_3_OD, 100 MHz) δ 176.3 (C-1), 174.2 (C-4), 135.8 (C-6), 134.4 (C-10a), 130.1 (C-6a), 129.5 (C-7), 127.5 (C-13), 127.34 (C-8/C-9), 127.26 (C-8/C-9), 126.5 (C-12), 123.7 (C-11), 123.6 (C-10), 49.0 (C-19), 46.1(C-17), 36.6 (C-15), 32.0 (C-2), 31.6 (C-3), 30.3 (C-23), 30.1 (C-22), 27.8 (C-16), 27.4 (C-20/C-21), 27.2 (C-20/C-21); (+)-HRESIMS *m/z* 737.4699 [M+H]^+^ (calcd for C_44_H_61_N_6_O_4_, 737.4749).

#### 4.2.15. *N*^1^*,N*^12^-Bis(3-(4-(Naphthalen-1-ylamino)-4-oxobutanamido)propyl)dodecane-1,12-diaminium 2,2,2-trifluoroacetate (**18f**)

Following general procedure B, the reaction of carboxylic acid **9** (58 mg, 0.24 mmol), di-*tert*-butyl dodecane-1,12-diylbis((3-aminopropyl)carbamate) (**16f**) (57 mg, 0.11 mmol), EDC·HCl (52 mg, 0.27 mmol), and DMAP (60 mg, 0.49 mmol) in CH_2_Cl_2_ (1.5 mL) created di-*tert*-butyl dodecane-1,12-diylbis((3-(4-(naphthalen-1-ylamino)-4-oxobutanamido)propyl)carbamate) as a colorless oil (62 mg, 58%). Following general procedure C, the reaction of a sub-sample of this material (26 mg, 0.027 mmol) in CH_2_Cl_2_ (2 mL) with TFA (0.2 mL) created the di-TFA salt **18f** as a pale-yellow oil after purification (20 mg, 75%). R*_f_* = 0.20 (RP-18, MeOH:10% HCl, 7:3); IR (ATR) *v*_max_ 3083, 2927, 1667, 1537, 1201, 1132, 800, 775 cm^−1^; ^1^H NMR (CD_3_OD, 400 MHz) δ 8.05 (2H, dd, *J* = 8.0, 1.5 Hz, H-10), 7.88 (2H, dd, *J* = 7.5, 2.0 Hz, H-7), 7.75 (2H, d, *J* = 8.0 Hz, H-13), 7.61 (2H, dd, *J* = 7.5, 1.0 Hz, H-11), 7.56–7.50 (4H, m, H-8, H-9), 7.46 (2H, t, *J* = 7.8 Hz, H-12), 3.34–3.30 (4H, m, H_2_-15), 2.95–2.91 (8H, m, H_2_-2, H_2_-17), 2.68–2.62 (8H, m, H_2_-3, H_2_-19), 1.86–1.80 (4H, m, H_2_-16), 1.48–1.41 (4H, m, H_2_-20), 1.21–1.08 (16H, m, H_2_-21, H_2_-22, H_2_-23, H_2_-24); ^13^C NMR (CD_3_OD, 100 MHz) δ 176.2 (C-1), 174.2 (C-4), 135.7 (C-6), 134.3 (C-10a), 130.0 (C-6a) 129.4 (C-7), 127.5 (C-13), 127.3 (C-8/C-9), 127.2 (C-8/C-9), 126.5 (C-12), 123.7 (C-11), 123.5 (C-10), 49.0 (C-19), 46.0 (C-17), 36.6 (C-15), 32.0 (C-2), 31.6 (C-3), 30.5 (C-22/C-23/C-24), 30.4 (C-22/C-23/C-24), 30.1 (C-22/C-23/C-24), 27.7 (C-16), 27.3 (C-20/C-21), 27.2 (C-20/C-21); (+)-HRESIMS [M+2H]^2+^ *m/z* 383.2575 (calcd for C_46_H_66_N_6_O_4_, 383.2567).

#### 4.2.16. *N*^1^*,N*^4^*-*Bis(3-(2-naphthamido)propyl)butane-1,4-diaminium 2,2,2-trifluoroacetate (**19a**)

Following general procedure A, the reaction of 2-napthoic acid (**10**) (94 mg, 0.55 mmol), di-*tert*-butyl butane-1,4-diylbis((3-aminopropyl)carbamate) (**16a**) (100 mg, 0.25 mmol), EDC·HCl (124 mg, 0.65 mmol), HOBt (87 mg, 0.64 mmol), and DIPEA (0.26 mL, 1.49 mmol) in CH_2_Cl_2_ (1.5 mL) created di-*tert*-butyl butane-1,4-diylbis((3-(2-naphthamido)propyl)carbamate) (91 mg, 51%) as a white solid. Following general procedure C, the reaction of a sub-sample of this material (72 mg, 0.10 mmol) in CH_2_Cl_2_ (2 mL) with TFA (0.2 mL) created the di-TFA salt **19a** as a beige solid after purification (71 mg, 96%). R*_f_* = 0.33 (RP-18, MeOH:10% HCl, 7:3); m.p. 132–135 °C; IR (ATR) *v*_max_ 3332, 2948, 2835, 1674, 1639, 1544, 1435, 1313, 1201, 1133, 1018, 721 cm^−1^; ^1^H NMR (CD_3_OD, 400 MHz) δ 8.38 (2H, br s, H-3), 7.96–7.87 (8H, m, H-4, H-7, H-8, H-9), 7.61–7.53 (4H, m, H-5, H-6), 3.57 (4H, t, *J* = 6.4 Hz, H_2_-11), 3.14–3.10 (8H, m, H_2_-13, H_2_-15), 2.08–2.01 (4H, m, H_2_-12), 1.90–1.87 (4H, m, H_2_-16); ^13^C NMR (CD_3_OD, 100 MHz) δ 171.2 (C-1), 136.4 (C-7a), 134.0 (C-3a), 132.2 (C-2), 130.0 (C-4), 129.5 (C-8), 129.1 (C-3/C-6), 129.0 (C-3/C-6), 128.8 (C-7), 128.0 (C-5), 124.8 (C-9), 48.2 (C-15), 46.5 (C-13), 37.5 (C-11), 27.9 (C-12), 24.4 (C-16); (+)-HRESIMS *m/z* 511.3071 [M+H]^+^ (calcd for C_32_H_39_N_4_O_2_, 511.3068).

#### 4.2.17. *N*^1^*,N*^6^-Bis(3-(2-naphthamido)propyl)hexane-1,6-diaminium 2,2,2-trifluoroacetate (**19b**)

Following general procedure A, the reaction of 2-naphthoic acid (**10**) (44.0 mg, 0.256 mmol), di-*tert*-butyl hexane-1,6-diylbis((3-aminopropyl)carbamate) (**16b**) (50.0 mg, 0.12 mmol), EDC·HCl (57.9 mg, 0.302 mmol), HOBt (40.8 mg, 0.30 mmol), and DIPEA (0.12 mL, 0.69 mmol) in CH_2_Cl_2_ (1.5 mL) created di-*tert*-butyl hexane-1,6-diylbis((3-(2-naphthamido)propyl)carbamate) as a colorless oil (48.0 mg, 54%). Following general procedure C, the reaction of a sub-sample of this material (6 mg, 0.0081 mmol) in CH_2_Cl_2_ (2 mL) with TFA (0.2 mL) created the di-TFA salt **19b** as a colorless gum (6 mg, 97%) with no further purification required. R*_f_* = 0.43 (RP-18, MeOH:10% HCl, 7:3); IR (ATR) *v*_max_ 3747, 3325, 2922, 2853, 2354, 1635, 1541, 1465, 1199 cm-^1^; ^1^H NMR (CD_3_OD, 400 MHz) δ 8.39 (2H, br s, H-3), 7.98–7.88 (8H, m, H-4, H-7, H-8, H-9), 7.62–7.55 (4H, m, H-5, H-6), 3.57 (4H, t, *J* = 6.5 Hz, H_2_-11), 3.09 (4H, t, *J* = 7.2 Hz, H_2_-13), 3.05 (4H, t, *J* = 8.0 Hz, H_2_-15), 2.06–1.99 (4H, m, H_2_-12), 1.80–1.74 (4H, m, H_2_-16), 1.54–1.50 (4H, m, H_2_-17); ^13^C NMR (CD_3_OD, 100 MHz) δ 171.3 (C-1), 136.5 (C-7a), 134.1 (C-3a), 131.5 (C-2), 130.0 (C-4), 129.5 (C-8), 129.1 (C-3/C-6), 129.0 (C-3/C-6), 128.9 (C-7), 128.0 (C-5), 124.7 (C-9), 49.0 (C-15), 46.4 (C-13), 37.5 (C-11), 28.0 (C-12), 27.2 (C-16/C-17), 27.1 (C-16/C-17); (+)-HRESIMS *m/z* 539.3387[M+H]^+^ (calcd for C_34_H_43_N_4_O_2_, 539.3381).

#### 4.2.18. *N*^1^*,N*^7^-Bis(3-(2-naphthamido)propyl)heptane-1,7-diaminium 2,2,2-trifluoroacetate (**19c**)

Following general procedure A, the reaction of 2-naphthoic acid (**10**) (42.6 mg, 0.247 mmol), di-*tert*-butyl heptane-1,7-diylbis((3-aminopropyl)carbamate) (**16c**) (50 mg, 0.11 mmol), EDC·HCl (56.1 mg, 0.293 mmol), HOBt (39.6 mg, 0.29 mmol), and DIPEA (0.118 mL, 0.677 mmol) in CH_2_Cl_2_ (1.5 mL) created di-*tert*-butyl heptane-1,7-diylbis((3-(2-naphthamido)propyl)carbamate) as a colorless oil (48 mg, 58%). Following general procedure C, the reaction of a sub-sample of this material (12 mg, 0.016 mmol) in CH_2_Cl_2_ (2 mL) with TFA (0.2 mL) created the di-TFA salt **19c** as a colorless gum after purification (6 mg, 48%). R*_f_* = 0.33 (RP-18, MeOH:10% HCl, 7:3); IR (ATR) *v*_max_ 3311, 2927, 2857, 1674, 1639, 1544, 1505, 1434, 1312, 1200, 1179, 1131, 876, 832, 799, 780, 762, 721 cm^−1^; ^1^H NMR (CD_3_OD, 400 MHz) δ 8.43 (2H, s, H-3), 8.03–7.90 (8H, m, H-4, H-7, H-8, H-9), 7.66–7.56 (4H, m, H-5, H-6), 3.59 (4H, t, *J* = 6.5 Hz, H_2_-11), 3.11 (4H, t, *J* = 7.0 Hz, H_2_-13), 3.05 (4H, t, *J* = 7.5 Hz, H_2_-15), 2.09–2.02 (4H, m, H_2_-12), 1.81–1.73 (4H, m, H_2_-16), 1.51–1.47 (6H, m, H_2_-17, H_2_-18); ^13^C NMR (CD_3_OD, 100 MHz) δ 171.2 (C-1), 136.4 (C-7a), 134.1 (C-3a), 132.2 (C-2), 130.0 (C-4), 129.5 (C-8), 129.1 (C-3/C-6), 129.0 (C-3/C-6), 128.8 (C-7), 128.0 (C-5), 124.8 (C-9), 49.0 (C-15), 46.4 (C-13), 37.5 (C-11), 29.7 (C-18), 27.9 (C-12), 27.3 (C-16/C-17), 27.2 (C-16/C-17); (+)-HRESIMS *m/z* 553.3520 [M+H]^+^ (calcd for C_35_H_45_N_4_O_2_, 553.3537).

#### 4.2.19. *N*^1^*,N*^8^-Bis(3-(2-naphthamido)propyl)octane-1,8-diaminium 2,2,2-trifluoroacetate (**19d**)

Following general procedure A, the reaction of 2-naphthoic acid (**10**) (41.3 mg, 0.240 mmol), di-*tert*-butyl octane-1,8-diylbis((3-aminopropyl)carbamate) (**16d**) (50 mg, 0.11 mmol), EDC·HCl (54.3 mg, 0.283 mmol), HOBt (38.3 mg, 0.28 mmol), and DIPEA (0.114 mL, 0.653 mmol) in CH_2_Cl_2_ (1.5 mL) created di-*tert*-butyl octane-1,8-diylbis((3-(2-naphthamido)propyl)carbamate) as a colorless oil (44 mg, 52%). Following general procedure C, the reaction of a sub-sample of this material (18 mg, 0.024 mmol) in CH_2_Cl_2_ (2 mL) with TFA (0.2 mL) created the di-TFA salt **19d** as a colorless gum after purification (16 mg, 84%). R*_f_* = 0.33 (RP-18, MeOH:10% HCl, 7:3); IR (ATR) *v*_max_ 3716, 2927, 2343, 1739, 1463, 1263, 801 cm-^1^; ^1^H NMR (CD_3_OD, 400 MHz) δ 8.40 (2H, s, H-3), 7.99–7.88 (8H, m, H-4, H-7, H-8, H-9), 7.62–7.56 (4H, m, H-5, H-6), 3.57 (4H, t, *J* = 6.5 Hz, H_2_-11), 3.09 (4H, t, *J* = 7.4 Hz, H_2_-13), 3.02 (4H, t, *J* = 7.8 Hz, H_2_-15), 2.10–1.99 (4H, m, H_2_-12), 1.80–1.69 (4H, m, H_2_-16), 1.50–1.43 (8H, m, H_2_-17, H_2_-18); ^13^C NMR (CD_3_OD, 100 MHz) δ 171.2 (C-1), 136.4 (C-7a), 134.1 (C-3a), 132.2 (C-2), 130.0 (C-4), 129.5 (C-8), 129.1 (C-3/C-6), 129.0 (C-3/C-6), 128.9 (C-7), 128.0 (C-5), 124.7 (C-9), 49.0 (C-15), 46.4 (C-13), 37.5 (C-11), 30.0 (C-18), 27.9 (C-12), 27.5 (C-16/C-17), 27.4 (C-16/C-17); (+)-HRESIMS *m/z* 567.3691 [M+H]^+^ (calcd for C_36_H_47_N_4_O_2_, 567.3694).

#### 4.2.20. *N*^1^*,N*^10^-Bis(3-(2-naphthamido)propyl)decane-1,10-diaminium 2,2,2-trifluoroacetate (**19e**) 

Following general procedure A, the reaction of 2-napthoic acid (**10**) (78 mg, 0.45 mmol), di-*tert*-butyl decane-1,10-diylbis((3-aminopropyl)carbamate) (**16e**) (100 mg, 0.21 mmol), EDC·HCl (103 mg, 0.54 mmol), HOBt (72 mg, 0.53 mmol), and DIPEA (0.22 mL, 1.26 mmol) in CH_2_Cl_2_ (1.5 mL) created di-*tert*-butyl decane-1,10-diylbis((3-(2-naphthamido)propyl)carbamate) (99 mg, 59%) as a colorless oil. Following general procedure C, the reaction of a sub-sample of this material (78 mg, 0.098 mmol) in CH_2_Cl_2_ (2 mL) with TFA (0.2 mL) created the di-TFA salt **19e** (76 mg, 94%) as a yellow oil with no further purification required. R*_f_* = 0.14 (RP-18, MeOH:10% HCl, 7:3); IR (ATR) *v*_max_ 3307,2944, 2832, 1685, 1448, 1115, 1022 cm^−1^; ^1^H NMR (CD_3_OD, 400 MHz) δ 8.40 (2H, br s, H-3), 7.98–7.89 (8H, m, H-4, H-7, H-8, H-9), 7.62–7.54 (4H, m, H-5, H-6), 3.57 (4H, t, *J* = 6.5 Hz, H_2_-11), 3.08 (4H, t, *J* = 7.2 Hz, H_2_-13), 3.01 (4H, t, *J* = 7.7 Hz, H_2_-15), 2.07–2.00 (4H, m, H_2_-12), 1.75–1.67 (4H, m, H_2_-16), 1.43–1.34 (12H, m, H_2_-17, H_2_-18, H_2_-19); ^13^C NMR (CD_3_OD, 100 MHz) δ 171.1 (C-1), 136.4 (C-7a), 134.1 (C-3a), 132.2 (C-2), 130.1 (C-4), 129.5 (C-8), 129.1 (C-3/C-6), 129.0 (C-3/C-6), 128.8 (C-7), 128.0 (C-5), 124.8 (C-9), 49.0 (C-15), 46.4 (C-13), 37.6 (C-11), 30.4 (C-18/C-19), 30.2 (C-18/C-19), 27.8 (C-12), 27.5 (C-17), 27.4 (C-16); (+)-HRESIMS *m/z* 595.3993 [M+H]^+^ (calcd for C_38_H_51_N_4_O_2_, 595.4007). 

#### 4.2.21. *N*^1^*,N*^12^-Bis(3-(2-naphthamido)propyl)dodecane-1,12-diaminium 2,2,2-trifluoroacetate (**19f**)

Following general procedure A, the reaction of 2-naphthoic acid (**10**) (55 mg, 0.32 mmol), di-*tert*-butyl dodecane-1,12-diylbis((3-aminopropyl)carbamate) (**16f**) (75 mg, 0.15 mmol), EDC·HCl (73 mg, 0.38 mmol), HOBt (51 mg, 0.38 mmol), and DIPEA (0.15 mL, 0.86 mmol) in CH_2_Cl_2_ (1.5 mL) created di-*tert*-butyl dodecane-1,12-diylbis((3-(2-naphthamido)propyl)carbamate) (35 mg, 28%) as a colorless oil. Following general procedure C, the reaction of a sub-sample of this material (20 mg, 0.024 mmol) in CH_2_Cl_2_ (2 mL) with TFA (0.2 mL) created the di-TFA salt **19f** (16 mg, 78%) as an orange oil with no further purification required. R*_f_* = 0.12 (RP-18, MeOH:10% HCl, 7:3); IR (ATR) *v*_max_ 3308, 2945, 2833, 1655, 1450, 1246, 1115, 1022 cm^−1^; ^1^H NMR (CD_3_OD, 400 MHz) δ 8.43 (2H, s, H-3), 8.01–7.91 (8H, m, H-4, H-7, H-8, H-9), 7.65–7.57 (4H, m, H-5, H-6), 3.59 (4H, t, *J* = 6.5 Hz, H_2_-11), 3.11 (4H, t, *J* = 7.3 Hz, H_2_-13), 3.04 (4H, t, *J* = 7.8 Hz, H_2_-15), 2.08–2.02 (4H, m, H_2_-12), 1.77–1.70 (4H, m, H_2_-16) 1.46–1.32 (16H, m, H_2_-17, H_2_-18, H_2_-19, H_2_-20); ^13^C NMR (CD_3_OD, 100 MHz) δ 171.2 (C-1), 136.4 (C-7a), 134.1 (C-3a), 132.2 (C-2), 130.0 (C-4), 129.5 (C-8), 129.1 (C-3/C-6), 129.0 (C-3/C-6), 128.8 (C-7), 128.0 (C-5), 124.7 (C-9), 49.1 (C-15), 46.4 (C-13), 37.5 (C-11), 30.7 (C-18/C-19/C-20), 30.5 (C-18/C-19/C-20), 30.3 (C-18/C-19/C-20), 27.9 (C-12), 27.5 (C-17), 27.4 (C-16); (+)-HRESIMS *m/z* 623.4306 [M+H]^+^ (calcd for C_40_H_55_N_4_O_2_, 623.4320). 

#### 4.2.22. *N*^1^*,N*^4^-Bis(3-(4-(naphthalen-2-ylamino)-4-oxobutanamido)propyl)butane-1,4-diaminium 2,2,2-trifluoroacetate (**20a**)

Following general procedure B, the reaction of carboxylic acid **11** (62 mg, 0.25 mmol), di-*tert*-butyl butane-1,4-diylbis((3-aminopropyl)carbamate) (**16a**) (48 mg, 0.12 mmol), EDC·HCl (55 mg, 0.29 mmol), and DMAP (62 mg, 0.51 mmol) in CH_2_Cl_2_ (1.5 mL) created di-*tert*-butyl butane-1,4-diylbis((3-(4-(naphthalen-2-ylamino)-4-oxobutanamido)propyl)carbamate) as a pale-pink oil (84 mg, 82%). Following general procedure C, the reaction of a sub-sample of this material (16 mg, 0.019 mmol) in CH_2_Cl_2_ (2.0 mL) with TFA (0.2 mL) created the di-TFA salt **20a** as a colorless oil after purification (15 mg, 90%). R*_f_* = 0.28 (RP-18, MeOH:10% HCl, 7:3); IR (ATR) *v*_max_ 3278, 1676, 1202, 1160, 799, 766 cm^−1^; ^1^H NMR (CD_3_OD, 400 MHz) δ 8.18 (2H, d, *J* = 2.0 Hz, H-7), 7.80–7.73 (6H, m, H-8, H-11, H-12), 7.54 (2H, dd, *J* = 9.0, 2.0 Hz, H-13), 7.46–7.42 (2H, m, H-9), 7.40–7.36 (2H, m, H-10), 3.32–3.28 (4H, m, H_2_-15), 2.87 (4H, t, *J* = 7.0 Hz, H_2_-17), 2.80–2.77 (4H, m, H_2_-2), 2.70–2.66 (4H, m, H_2_-19), 2.61–2.58 (4H, m, H_2_-3), 1.84–1.77 (4H, m, H_2_-16), 1.46–1.42 (4H, m, H_2_-20); ^13^C NMR (CD_3_OD, 100 MHz) δ 176.5 (C-1), 173.2 (C-4), 137.5 (C-6), 135.2 (C-7a), 131.9 (C-11a), 129.7 (C-12), 128.7 (C-11), 128.4 (C-8), 127.6 (C-9), 126.0, (C-10), 121.2 (C-13), 117.4 (C-7), 47.9 (C-19), 45.8 (C-17), 36.4 (C-15), 32.4 (C-2), 31.3 (C-3), 27.8 (C-16), 24.0 (C-20); (+)-HRESIMS *m/z* 653.3823 [M+H]^+^ (calcd for C_38_H_49_N_6_O_4_, 653.3810).

#### 4.2.23. *N*^1^*,N*^6^-Bis(3-(4-(naphthalen-2-ylamino)-4-oxobutanamido)propyl)hexane-1,6-diaminium 2,2,2-trifluoroacetate (**20b**)

Following general procedure B, the reaction of carboxylic acid **11** (71 mg, 0.29 mmol), di-*tert*-butyl hexane-1,6-diylbis((3-aminopropyl)carbamate) (**16b**) (50 mg, 0.12 mmol), EDC·HCl (62 mg, 0.32 mmol), and DMAP (71 mg, 0.58 mmol) in CH_2_Cl_2_ (1.5 mL) created di-*tert*-butylhexane-1,6-diylbis((3-(4-(naphthalen-2-ylamino)-4-oxobutanamido)propyl)carbamate) as a pale brown oil (53 mg, 50%). Following general procedure C, the reaction of a sub-sample of this material (26 mg, 0.030 mmol) in CH_2_Cl_2_ (2 mL) with TFA (0.2 mL) created the di-TFA salt **20b** as a yellow oil after purification (24 mg, 88%). R*_f_* = 0.32 (RP-18, MeOH:10% HCl, 7:3); IR (ATR) *v*_max_ 3279, 1671, 1542, 1201, 1132, 800, 777 cm^−1^; ^1^H NMR (CD_3_OD, 400 MHz) δ 8.20 (2H, d, *J* = 2.0 Hz, H-7), 7.78–7.71 (6H, m, H-8, H-11, H-12), 7.54 (2H, dd, *J* = 8.5, 2.0 Hz, H-13), 7.43 (2H, td, *J* = 7.5, 1.5 Hz, H-9), 7.37 (2H, td, *J* = 7.5, 1.5 Hz, H-10), 3.35–3.30 (4H, m, H_2_-15), 2.96 (4H, t, *J* = 6.8 Hz, H_2_-17), 2.80 (4H, t, *J* = 6.5 Hz, H_2_-2), 2.63–2.59 (8H, m, H_2_-3, H_2_-19), 1.88–1.82 (4H, m, H_2_-16), 1.31–1.27 (4H, m, H_2_-20), 0.83–0.81 (4H, m, H_2_-21); ^13^C NMR (CD_3_OD, 100 MHz) δ 176.5 (C-1), 173.2 (C-4), 137.6 (C-6), 135.3 (C-7a), 131.9 (C-11a), 129.6 (C-12), 128.7 (C-11), 128.5 (C-8), 127.6 (C-9), 126.0, (C-10), 121.1 (C-13), 117.3 (C-7), 48.7 (C-19), 45.8 (C-17), 36.4 (C-15), 32.4 (C-2), 31.2 (C-3), 27.9 (C-16), 26.8 (C-20/C-21), 26.6 (C-20/C-21); (+)-HRESIMS [M+H]^+^ *m/z* 681.4106 (calcd for C_40_H_53_N_6_O_4_, 681.4123).

#### 4.2.24. *N*^1^*,N*^7^-Bis(3-(4-(naphthalen-2-ylamino)-4-oxobutanamido)propyl)heptane-1,7-diaminium 2,2,2-trifluoroacetate (**20c**)

Following general procedure B, the reaction of carboxylic acid **11** (68 mg, 0.29 mmol), di-*tert*-butyl heptane-1,7-diylbis((3-aminopropyl)carbamate) (**16c**) (50 mg, 0.11 mmol), EDC·HCl (60 mg, 0.31 mmol), and DMAP (69 mg, 0.57 mmol) in CH_2_Cl_2_ (1.5 mL) created di-*tert*-butyl heptane-1,7-diylbis((3-(4-(naphthalen-2-ylamino)-4-oxobutanamido)propyl)carbamate) as a colorless oil (64 mg, 65%). Following general procedure C, the reaction of a sub-sample of this material (44 mg, 0.049 mmol) in CH_2_Cl_2_ (2 mL) with TFA (0.2 mL) created the di-TFA salt **20c** as a colorless oil after purification (42 mg, 93%). R*_f_* = 0.29 (RP-18, MeOH:10% HCl, 7:3); IR (ATR) *v*_max_ 3289, 3027 1666, 1537, 1201, 1131, 800, 723 cm^−1^; ^1^H NMR (CD_3_OD, 400 MHz) δ 8.20 (2H, d, *J* = 2.0 Hz, H-7), 7.77–7.71 (6H, m, H-8, H-11, H-12), 7.54 (2H, dd, *J* = 9.0, 2.0 Hz, H-13), 7.42 (2H, td, *J* = 7.5, 1.3 Hz, H-9), 7.36 (2H, td, *J* = 8.3, 1.5 Hz, H-10), 3.35–3.30 (4H, m, H_2_-15), 3.00 (4H, t, *J* = 7.0 Hz, H_2_-17) 2.81–2.78 (4H, m, H_2_-2), 2.67 (4H, t, *J* = 8.0 Hz, H_2_-19), 2.62–2.59 (4H, m, H_2_-3), 1.89–1.83 (4H, m, H_2_-16), 1.37–1.29 (4H, m, H_2_-20), 0.81–0.76 (6H, m, H_2_-21, H_2_-22); ^13^C NMR (CD_3_OD, 100 MHz) δ 176.6 (C-1), 173.2 (C-4), 137.6 (C-6), 135.3 (C-7a), 131.9 (C-11a), 129.6 (C-12), 128.6 (C-8/C-11), 128.5 (C-8/C-11), 127.6 (C-9), 126.0, (C-10), 121.0 (C-13), 117.2 (C-7), 48.9 (C-19), 45.8 (C-17), 36.4 (C-15), 32.4 (C-2), 31.2 (C-3), 29.4 (C-22), 27.9 (C-16), 27.01 (C-20/C-21), 26.96 (C-20/C-21); (+)-HRESIMS [M+H]^+^ *m/z* 695.4283 (calcd for C_41_H_55_N_6_O_4_, 695.4279).

#### 4.2.25. *N*^1^*,N*^8^-Bis(3-(4-(naphthalen-2-ylamino)-4-oxobutanamido)propyl)octane-1,8-diaminium 2,2,2-trifluoroacetate (**20d**)

Following general procedure B, the reaction of carboxylic acid **11** (67 mg, 0.27 mmol), di-*tert*-butyl octane-1,8-diylbis((3-aminopropyl)carbamate) (**16d**) (50 mg, 0.11 mmol), EDC·HCl (59 mg, 0.31 mmol) and DMAP (67 mg, 0.55 mmol) in CH_2_Cl_2_ (1.5 mL) created di-*tert*-butyl octane-1,8-diylbis((3-(4-(naphthalen-2-ylamino)-4-oxobutanamido)propyl)carbamate) as a colorless oil (40 mg, 40%). Following general procedure C, the reaction of this material (40 mg, 0.044 mmol) in CH_2_Cl_2_ (2 mL) with TFA (0.2 mL) created the di-TFA salt **20d** as a pale purple oil after purification (37 mg, 90%). R*_f_* = 0.28 (RP-18, MeOH:10% HCl, 7:3); IR (ATR) *v*_max_ 3288, 3061 1669, 1553, 1200, 1132, 800, 721 cm^−1^; ^1^H NMR (CD_3_OD, 400 MHz) δ 8.20 (2H, d, *J* = 2.0 Hz, H-7), 7.77–7.71 (6H, m, H-8, H-11, H-12), 7.55 (2H, dd, *J* = 9.0, 2.0 Hz, H-13), 7.44–7.40 (2H, m, H-9), 7.39–7.34 (2H, m, H-10), 3.36–3.30 (4H, m, H_2_-15), 3.01 (4H, t, *J* = 7.0 Hz, H_2_-17), 2.82–2.78 (4H, m, H_2_-2), 2.71 (4H, t, *J* = 8.0 Hz, H_2_-19), 2.62–2.59 (4H, m, H_2_-3), 1.90–1.84 (4H, m, H_2_-16), 1.42–1.36 (4H, m, H_2_-20), 0.85–0.80 (8H, m, H_2_-21, H_2_-22); ^13^C NMR (CD_3_OD, 100 MHz) δ 176.5 (C-1), 173.2 (C-4), 137.6 (C-6), 135.3 (C-7a), 131.9 (C-11a), 129.6 (C-12), 128.6 (C-8/C-11), 128.4 (C-8/C-11), 127.5 (C-9), 125.9, (C-10), 121.0 (C-13), 117.2 (C-7), 48.9 (C-19), 45.8 (C-17), 36.3 (C-15), 32.3 (C-2), 31.2 (C-3), 29.7 (C-22), 27.9 (C-16), 27.14 (C-20/C-21), 27.07 (C-20/C-21); (+)-HRESIMS [M+2H]^2+^ *m/z* 355.2252 (calcd for C_42_H_58_N_6_O_4_, 355.2254).

#### 4.2.26. *N*^1^*,N*^10^-Bis(3-(4-(naphthalen-2-ylamino)-4-oxobutanamido)propyl)decane-1,10-diaminium 2,2,2-trifluoroacetate (**20e**)

Following general procedure B, the reaction of carboxylic acid **11** (63 mg, 0.26 mmol), di-*tert*-butyl decane-1,10-diylbis((3-aminopropyl)carbamate) (**16e**) (50 mg, 0.10 mmol), EDC·HCl (55 mg, 0.29 mmol), and DMAP (63 mg, 0.52 mmol) in CH_2_Cl_2_ (1.5 mL) created di-*tert*-butyl decane-1,10-diylbis((3-(4-(naphthalen-2-ylamino)-4-oxobutanamido)propyl)carbamate) as a colorless oil (78 mg, 83%). Following general procedure C, the reaction of a sub-sample of this material (41 mg, 0.044 mmol) in CH_2_Cl_2_ (2.0 mL) with TFA (0.2 mL) created the di-TFA salt **20e** as a colorless oil after purification (38 mg, 89%). R*_f_* = 0.17 (RP-18, MeOH: 10% HCl, 7:3); IR (ATR) *v*_max_ 3293, 1679, 1538, 1202, 1132, 721 cm^−1^; ^1^H NMR (CD_3_OD, 400 MHz) δ 8.21 (2H, d, *J* = 2.0 Hz, H-7), 7.78–7.72 (6H, m, H-8, H-11, H-12), 7.55 (2H, dd, *J* = 8.5, 2.0 Hz, H-13), 7.44–7.39 (2H, m, H-9), 7.37–7.33 (2H, m, H-10), 3.36–3.30 (4H, m, H_2_-15), 3.02 (4H, t, *J* = 7.0 Hz, H_2_-17), 2.81–2.74 (8H, m, H_2_-2, H_2_-19), 2.62–2.59 (4H, m, H_2_-3), 1.90–1.83 (4H, m, H_2_-16), 1.49–1.41 (4H, m, H_2_-20), 0.96–0.88 (12H, m, H_2_-21, H_2_-22, H_2_-23); ^13^C NMR (CD_3_OD, 100 MHz) δ 176.5 (C-1), 173.2 (C-4), 137.7 (C-6), 135.3 (C-7a), 132.0 (C-11a), 129.7 (C-12), 128.7 (C-11), 128.5 (C-8), 127.6 (C-9), 126.0 (C-10), 121.1 (C-13), 117.3 (C-7), 49.1 (C-19), 45.9 (C-17), 36.4 (C-15), 32.4 (C-2), 31.3 (C-3), 30.2 (C-23), 30.0 (C-22), 27.9 (C-16), 27.32 (C-20/C-21), 27.28 (C-20/C-21); (+)-HRESIMS *m/z* 737.4736 [M+H]^+^ (calcd for C_44_H_61_N_6_O_4_, 737.4749).

#### 4.2.27. *N*^1^*,N*^12^-Bis(3-(4-(naphthalen-2-ylamino)-4-oxobutanamido)propyl)dodecane-1,12-diaminium 2,2,2-trifluoroacetate (**20f**)

Following general procedure B, the reaction of carboxylic acid **11** (58 mg, 0.24 mmol), di-*tert*-butyl dodecane-1,12-diylbis((3-aminopropyl)carbamate) (**16f**) (57 mg, 0.11 mmol), EDC·HCl (52 mg, 0.27 mmol), and DMAP (60 mg, 0.49 mmol) in CH_2_Cl_2_ (1.5 mL) created di-*tert*-butyl dodecane-1,12-diylbis((3-(4-(naphthalen-2-ylamino)-4-oxobutanamido)propyl)carbamate) as a colorless oil (81 mg, 76%). Following general procedure C, the reaction of a sub-sample of this material (28 mg, 0.029 mmol) in CH_2_Cl_2_ (2 mL) with TFA (0.2 mL) created the di-TFA salt **20f** as a colorless oil after purification (26 mg, 90%). R*_f_* = 0.13 (RP-18, MeOH:10% HCl, 7:3); IR (ATR) *v*_max_ 2916, 1769, 1671, 1503, 1202, 1037, 802 cm^−1^; ^1^H NMR (CD_3_OD, 400 MHz) δ 8.21 (2H, d, *J* = 1.5 Hz, H-7), 7.79–7.72 (6H, m, H-8, H-11, H-12), 7.56 (2H, dd, *J* = 9.0, 2.0 Hz, H-13), 7.44–7.40 (2H, m, H-9), 7.38–7.34 (2H, m, H-10), 3.36–3.30 (4H, m, H_2_-15), 3.02 (4H, t, *J* = 7.0 Hz, H_2_-17), 2.81–2.75 (4H, m, H_2_-2, H_2_-19), 2.62–2.59 (4H, m, H_2_-3), 1.90–1.84 (4H, m, H_2_-16), 1.51–1.44 (4H, m, H_2_-20), 1.04–0.99 (16H, m, H_2_-21, H_2_-22, H_2_-23, H_2_-24); ^13^C NMR (CD_3_OD, 100 MHz) δ 176.5 (C-1), 173.2 (C-4), 137.6 (C-6), 135.3 (C-7a), 131.9 (C-11a), 129.6 (C-12), 128.6 (C-8/C-11), 128.4 (C-8/C-11), 127.5 (C-9), 125.9 (C-10), 121.0 (C-13), 117.2 (C-7), 49.1 (C-19), 45.8 (C-17), 36.4 (C-15), 32.4 (C-2), 31.2 (C-3), 30.4 (C-22/C-23/C-24), 30.3 (C-22/C-23/C-24), 30.1 (C-22/C-23/C-24), 27.9 (C-16), 27.3 (C-20/C-21), 27.2 (C-20/C-21); (+)-HRESIMS [M+2H]^2+^ *m/z* 383.2578 (calcd for C_46_H_66_N_6_O_4_, 383.2567).

#### 4.2.28. *N*^1^*,N*^4^-Bis(3-(4-([1,1’-biphenyl]-4-ylamino)-4-oxobutanamido)propyl)butane-1,4-diaminium 2,2,2-trifluoroacetate (**21a**)

Following general procedure A, the reaction of carboxylic acid **12** (74 mg, 0.27 mmol) with di-*tert*-butyl butane-1,4-diylbis((3-aminopropyl)carbamate) (**16a**) (50 mg, 0.12 mmol), EDC·HCl (62 mg, 0.32 mmol), HOBt (44 mg, 0.32 mmol), and DIPEA (0.13 mL, 0.75 mmol) in CH_2_Cl_2_ (1.5 mL) created di-*tert*-butyl butane-1,4-diylbis((3-(4-([1,1’-biphenyl]-4-ylamino)-4-oxobutanamido)propyl)carbamate) as a pink solid (65 mg, 60%). Following general procedure C, the reaction of a sub-sample of this material (20 mg, 0.022 mmol) in CH_2_Cl_2_ (2 mL) with TFA (0.2 mL) created the di-TFA salt **21a** as a yellow solid after purification (15 mg, 73%). R*_f_* = 0.37 (RP-18, MeOH:10% HCl, 5:1); m.p. 203–205 °C; IR (ATR) *v*_max_ 3290, 3099, 3033, 2831, 1669, 1599, 1531, 1200, 1178, 1130, 835, 764, 720 cm^−1^; ^1^H NMR (DMSO-*d_6_*, 400 MHz) δ 10.08 (2H, br s, NH-5), 8.61–8.47 (4H, m, NH_2_-18), 8.11 (2H, t, *J* = 6.0 Hz, NH-14), 7.71–7.68 (4H, m, H-7), 7.65–7.58 (8H, m, H-8, H-11), 7.46–7.40 (4H, m, H-12), 7.35–7.29 (2H, m, H-13), 3.13 (4H, dt, *J* = 6.4, 6.2 Hz, H_2_-15), 2.93–2.82 (8H, m, H_2_-17, H_2_-19), 2.61 (4H, t, *J* = 7.0 Hz, H_2_-2/H_2_-3), 2.43 (4H, t, *J* = 7.0 Hz, H_2_-2/H_2_-3), 1.72 (4H, tt, *J* = 7.4, 6.4 Hz, H_2_-16), 1.63–1.54 (4H, m, H_2_-20); ^13^C NMR (DMSO-*d_6_*, 100 MHz) δ 172.0 (C-1), 170.5 (C-4), 139.7 (C-10), 138.8 (C-6), 134.6 (C-9), 128.9 (C-12), 127.0 (C-13), 126.8 (C-8), 126.2 (C-11), 119.3 (C-7), 46.1 (C-19), 44.5 (C-17), 35.5 (C-15), 31.5 (C-2/C-3), 30.1 (C-2/C-3), 26.1 (C-16), 22.6 (C-20); (+)-HRESIMS *m/z* 705.4118 [M+H]^+^ (calcd for C_42_H_53_N_6_O_4_, 705.4123).

#### 4.2.29. *N*^1^*,N*^6^-Bis(3-(4-([1,1’-biphenyl]-4-ylamino)-4-oxobutanamido)propyl)hexane-1,6-diaminium 2,2,2-trifluoroacetate (**21b**)

Following general procedure A, the reaction of carboxylic acid **12** (69 mg, 0.26 mmol) with di-*tert*-butyl hexane-1,6-diylbis((3-aminopropyl)carbamate) (**16b**) (50 mg, 0.12 mmol), EDC·HCl (58 mg, 0.30 mmol), HOBt (41 mg, 0.30 mmol), and DIPEA (0.12 mL, 0.69 mmol) in CH_2_Cl_2_ (1.5 mL) created di-*tert*-butyl hexane-1,6-diylbis((3-(4-([1,1’-biphenyl]-4-ylamino)-4-oxobutanamido)propyl)carbamate) as a pink solid (54 mg, 48%). Following general procedure C, the reaction of a sub-sample of this material (20 mg, 0.021 mmol) in CH_2_Cl_2_ (2 mL) with TFA (0.2 mL) created the di-TFA salt **21b** as a yellow solid after purification (15 mg, 74%). R*_f_* = 0.43 (RP-18, MeOH:10% HCl, 5:1); m.p. 200–202 °C; IR (ATR) *v*_max_ 3296, 3031, 2947, 2832, 2528, 1681, 1657, 1637, 1529, 1198, 1173, 1123, 1000, 759, 718 cm^−1^; ^1^H NMR (DMSO-*d_6_*, 400 MHz) δ 10.08 (2H, br s, NH-5), 8.54–8.29 (4H, m, NH_2_-18), 8.12 (2H, t, *J* = 5.8 Hz, NH-14), 7.71–7.65 (4H, m, H-7), 7.65–7.58 (8H, m, H-8, H-11), 7.46–7.40 (4H, m, H-12), 7.35–7.29 (2H, m, H-13), 3.14 (4H, dt, *J* = 6.4, 6.2 Hz, H_2_-15), 2.86 (4H, t, *J* = 7.2 Hz, H_2_-17), 2.77 (4H, t, *J* = 7.8 Hz, H_2_-19), 2.61 (4H, t, *J* = 6.8 Hz, H_2_-2/H_2_-3), 2.44 (4H, t, *J* = 6.8 Hz, H_2_-2/H_2_-3), 1.71 (4H, tt, *J* = 7.2, 6.7 Hz, H_2_-16), 1.53–1.43 (4H, m, H_2_-20), 1.23–1.15 (4H, m, H_2_-21); ^13^C NMR (DMSO-*d_6_*, 100 MHz) δ 172.1 (C-1), 170.6 (C-4), 139.7 (C-10), 138.8 (C-6), 134.5 (C-9), 128.9 (C-12), 127.0 (C-13), 126.8 (C-8), 126.1 (C-11), 119.2 (C-7), 46.7 (C-19), 44.5 (C-17), 35.4 (C-15), 31.4 (C-2/C-3), 30.1 (C-2/C-3), 26.2 (C-16), 25.4 (C-21), 25.3 (C-20); (+)-HRESIMS *m/z* 733.4415 [M+H]^+^ (calcd for C_44_H_57_N_6_O_4_, 733.4436).

#### 4.2.30. *N*^1^*,N*^7^-Bis(3-(4-([1,1’-biphenyl]-4-ylamino)-4-oxobutanamido)propyl)heptane-1,7-diaminium 2,2,2-trifluoroacetate (**21c**)

Following general procedure A, the reaction of carboxylic acid **12** (66 mg, 0.25 mmol) with di-*tert*-butyl heptane-1,7-diylbis((3-aminopropyl)carbamate) (**16c**) (50 mg, 0.11 mmol), EDC·HCl (56 mg, 0.29 mmol), HOBt (39 mg, 0.29 mmol), and DIPEA (0.12 mL, 0.69 mmol) in CH_2_Cl_2_ (1.5 mL) created di-*tert*-butyl heptane-1,7-diylbis((3-(4-([1,1’-biphenyl]-4-ylamino)-4-oxobutanamido)propyl)carbamate) as a pink solid (52 mg, 50%). Following general procedure C, the reaction of a sub-sample of this material (20 mg, 0.021 mmol) in CH_2_Cl_2_ (2 mL) with TFA (0.2 mL) created the di-TFA salt **21c** as a yellow solid after purification (14 mg, 68%). R*_f_* = 0.51 (RP-18, MeOH:10% HCl, 5:1); m.p. 178–180 °C; IR (ATR) *v*_max_ 3288, 3034, 2939, 2859, 2527, 1658, 1637, 1533, 1198, 1173, 1134, 763, 719 cm^−1^; ^1^H NMR (DMSO-*d_6_*, 400 MHz) δ 10.08 (2H, br s, NH-5), 8.48–8.33 (4H, m, NH_2_-18), 8.12 (2H, t, *J* = 5.8 Hz, NH-14), 7.71–7.65 (4H, m, H-7), 7.65–7.57 (8H, m, H-8, H-11), 7.46–7.40 (4H, m, H-12), 7.35–7.29 (2H, m, H-13), 3.14 (4H, dt, *J* = 6.4, 6.2 Hz, H_2_-15), 2.86 (4H, t, *J* = 7.0 Hz, H_2_-17), 2.75 (4H, t, *J* = 7.0 Hz, H_2_-19), 2.61 (4H, t, *J* = 6.6 Hz, H_2_-2/H_2_-3), 2.44 (4H, t, *J* = 6.6 Hz, H_2_-2/H_2_-3), 1.71 (4H, tt, *J* = 7.0, 6.4 Hz, H_2_-16), 1.53–1.42 (4H, m, H_2_-20), 1.20–1.10 (6H, m, H_2_-21, H_2_-22); ^13^C NMR (DMSO-*d_6_*, 100 MHz) δ 172.3 (C-1), 170.6 (C-4), 139.7 (C-10), 138.8 (C-6), 134.5 (C-9), 128.9 (C-12), 127.0 (C-13), 126.8 (C-8), 126.1 (C-11), 119.2 (C-7), 46.7 (C-19), 44.4 (C-17), 35.4 (C-15), 31.4 (C-2/C-3), 30.1 (C-2/C-3), 28.0 (C-21/C-22), 26.2 (C-16), 25.7 (C-21/C-22), 25.4 (C-20); (+)-HRESIMS *m/z* 747.4566 [M+H]^+^ (calcd for C_45_H_59_N_6_O_4_, 747.4592).

#### 4.2.31. *N*^1^*,N*^8^-Bis(3-(4-([1,1’-biphenyl]-4-ylamino)-4-oxobutanamido)propyl)octane-1,8-diaminium 2,2,2-trifluoroacetate (**21d**)

Following general procedure A, the reaction of carboxylic acid **12** (65 mg, 0.24 mmol) with di-*tert*-butyl octane-1,8-diylbis((3-aminopropyl)carbamate) (**16d**) (50 mg, 0.11 mmol), EDC·HCl (54 mg, 0.28 mmol), HOBt (38 mg, 0.28 mmol), and DIPEA (0.11 mL, 0.63 mmol) in CH_2_Cl_2_ (1.5 mL) created di-*tert*-butyl octane-1,8-diylbis((3-(4-([1,1’-biphenyl]-4-ylamino)-4-oxobutanamido)propyl)carbamate) as a pink solid (56 mg, 53%). Following general procedure C, the reaction of a sub-sample of this material (20 mg, 0.021 mmol) in CH_2_Cl_2_ (2 mL) with TFA (0.2 mL) created the di-TFA salt **21d** as a yellow solid after purification (14 mg, 67%). R*_f_* = 0.51 (RP-18, MeOH:10% HCl, 5:1); m.p. 182–184 °C; IR (ATR) *v*_max_ 3411, 3292, 3049, 2937, 2860, 2255, 1674, 1534, 1200, 1173, 1129, 1024, 1003, 764, 719 cm^−1^; ^1^H NMR (DMSO-*d_6_*, 400 MHz) δ 10.08 (2H, br s, NH-5), 8.48–8.34 (4H, m, NH_2_-18), 8.13 (2H, t, *J* = 6.0 Hz, NH-14), 7.71–7.65 (4H, m, H-7), 7.65–7.57 (8H, m, H-8, H-11), 7.46–7.40 (4H, m, H-12), 7.35–7.29 (2H, m, H-13), 3.15 (4H, dt, *J* = 6.4, 6.0 Hz, H_2_-15), 2.87 (4H, t, *J* = 7.0 Hz, H_2_-17), 2.76 (4H, t, *J* = 7.5 Hz, H_2_-19), 2.61 (4H, t, *J* = 6.8 Hz, H_2_-2/H_2_-3), 2.43 (4H, t, *J* = 6.8 Hz, H_2_-2/H_2_-3), 1.71 (4H, tt, *J* = 7.0, 6.4 Hz, H_2_-16), 1.53–1.41 (4H, m, H_2_-20), 1.19–1.08 (8H, m, H_2_-21, H_2_-22); ^13^C NMR (DMSO-*d_6_*, 100 MHz) δ 172.2 (C-1), 170.6 (C-4), 139.7 (C-10), 138.8 (C-6), 134.5 (C-9), 128.9 (C-12), 127.0 (C-13), 126.8 (C-8), 126.1 (C-11), 119.2 (C-7), 46.8 (C-19), 44.4 (C-17), 35.4 (C-15), 31.4 (C-2/C-3), 30.0 (C-2/C-3), 28.3 (C-21/C-22), 26.2 (C-16), 25.8 (C-21/C-22), 25.4 (C-20); (+)-HRESIMS *m/z* 761.4739 [M+H]^+^ (calcd for C_46_H_61_N_6_O_4_, 761.4749).

#### 4.2.32. *N*^1^*,N*^10^-Bis(3-(4-([1,1’-biphenyl]-4-ylamino)-4-oxobutanamido)propyl)decane-1,10-diaminium 2,2,2-trifluoroacetate (**21e**)

Following general procedure A, the reaction of carboxylic acid **12** (61 mg, 0.23 mmol) with di-*tert*-butyl decane-1,10-diylbis((3-aminopropyl)carbamate) (**16e**) (50 mg, 0.10 mmol), EDC·HCl (51 mg, 0.27 mmol), HOBt (36 mg, 0.27 mmol), and DIPEA (0.11 mL, 0.63 mmol) in CH_2_Cl_2_ (1.5 mL) created di-*tert*-butyl decane-1,10-diylbis((3-(4-([1,1’-biphenyl]-4-ylamino)-4-oxobutanamido)propyl)carbamate) as a pale yellow solid (56 mg, 57%). Following general procedure C, the reaction of a sub-sample of this material (20 mg, 0.020 mmol) in CH_2_Cl_2_ (2 mL) with TFA (0.2 mL) created the di-TFA salt **21e** as a yellow solid after purification (15 mg, 74%). R*_f_* = 0.34 (RP-18, MeOH:10% HCl, 5:1); m.p. 184–186 °C; IR (ATR) *v*_max_ 3287, 3099, 3033, 2930, 2854, 1668, 1599, 1533, 1200, 1175, 1130, 764, 720 cm^−1^; ^1^H NMR (DMSO-*d_6_*, 400 MHz) δ 10.09 (2H, br s, NH-5), 8.49–8.37 (4H, m, NH_2_-18), 8.14 (2H, t, *J* = 6.0 Hz, NH-14), 7.71–7.66 (4H, m, H-7), 7.64–7.57 (8H, m, H-8, H-11), 7.46–7.39 (4H, m, H-12), 7.34–7.28 (2H, m, H-13), 3.15 (4H, dt, *J* = 6.4, 6.2 Hz, H_2_-15), 2.93–2.83 (4H, m, H_2_-17), 2.82–2.72 (4H, m, H_2_-19), 2.62 (4H, t, *J* = 6.8 Hz, H_2_-2/H_2_-3), 2.44 (4H, t, *J* = 6.8 Hz, H_2_-2/H_2_-3), 1.72 (4H, tt, *J* = 7.0, 6.4 Hz, H_2_-16), 1.53–1.42 (4H, m, H_2_-20), 1.20–1.07 (12H, m, H_2_-21, H_2_-22, H_2_-23); ^13^C NMR (DMSO-*d_6_*, 100 MHz) δ 172.3 (C-1), 170.6 (C-4), 139.7 (C-10), 138.8 (C-6), 134.5 (C-9), 128.9 (C-12), 127.0 (C-13), 126.8 (C-8), 126.1 (C-11), 119.2 (C-7), 46.9 (C-19), 44.4 (C-17), 35.4 (C-15), 31.4 (C-2/C-3), 30.1 (C-2/C-3), 28.8 (C-21/C-22/C-23), 28.5 (C-21/C-22/C-23), 26.2 (C-16), 25.9 (C-21/C-22/C-23), 25.5 (C-20); (+)-HRESIMS *m/z* 789.5055 [M+H]^+^ (calcd for C_48_H_65_N_6_O_4_, 789.5062).

### 4.3. Antimicrobial Assays

The susceptibility of bacterial strains *S. aureus* (ATCC 25,923 or 29213), *E. coli* (ATCC 25922), and *P. aeruginosa* (ATCC 27853) to antibiotics and compounds was determined according to previously reported protocols [27]. Additional antimicrobial evaluation against MRSA (ATCC 43300), *Klebsiella pneumoniae* (ATCC 700603), *Acinetobacter baumannii* (ATCC 19606), *Candida albicans* (ATCC 90028), and *Cryptococcus neoformans* (ATCC 208821) was undertaken at the Community for Open Antimicrobial Drug Discovery at The University of Queensland (Australia) according to their standard protocols as reported previously [27,36].

### 4.4. Determination of the MICs of Antibiotics in the Presence of Synergizing Compounds

Restoring enhancer concentrations were determined using previously reported protocols [27].

### 4.5. Cytotoxicity Assays

Cytotoxicity assays were conducted using the protocols previously reported [27,36].

### 4.6. Hemolytic Assays

Hemolysis assays were conducted using the protocols previously reported [27,36].

### 4.7. Real-Time Growth Curves

Solutions of compound **20f** at concentrations of 2, 4, and 16 µg/mL were each tested in triplicate against *S. aureus* (ATCC 25923), MRSA (CF-Marseille), and *E. coli* (ATCC 25922) following previously reported protocols [27].

### 4.8. Minimum Bactericidal Concentration Test

MBC’s were determined following previously reported protocols [27].

## 5. Conclusions

A series of α,ω-disubstituted polyamines bearing naphthyl and biphenyl capping groups were prepared and evaluated for antimicrobial properties, as well as for the ability to enhance the action of doxycycline and erythromycin towards Gram-negative bacteria. Several analogues were identified as exhibiting pronounced antibacterial activity towards MRSA, with some examples also possessing antifungal activity towards *C. neoformans*. One particular analogue, **20f**, was found to act as a bactericide. In contrast to previously reported structurally related disubstituted polyamines, the naphthyl-containing analogues were able to restore the action of legacy antibiotics with two examples, enhancing activity over 32-fold. These current results suggest that naphthyl capped polyamines may represent a particularly useful scaffold to further explore in the search for non-toxic antibiotic enhancers.

## Figures and Tables

**Scheme 2:**
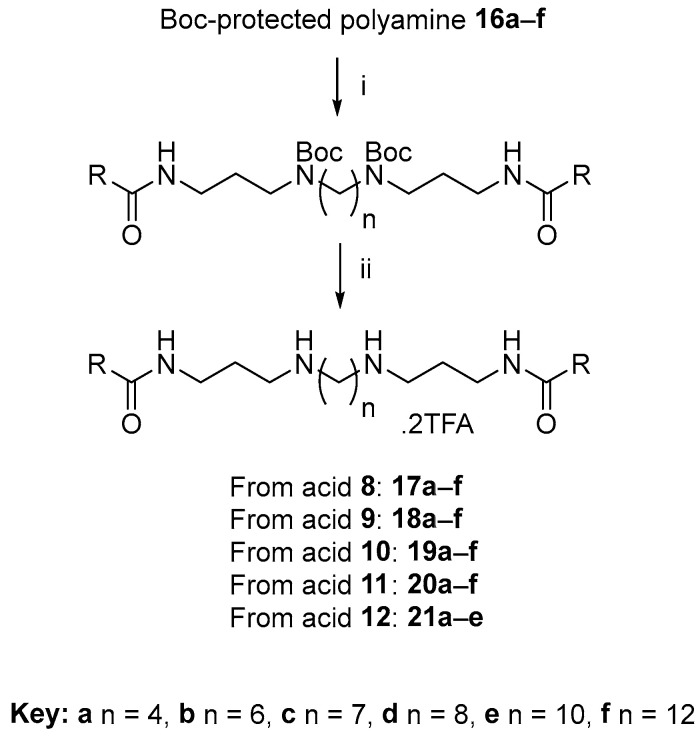
General method for the synthesis of target polyamine analogues **17**–**21**. *Reagents and conditions*: (i) Carboxylic acid RCO_2_H (**8**, **10**, **12**) (2.2 equiv.), Boc-protected polyamine (**16a**–**f**) (1 equiv.), with EDC·HCl/HOBt (2.6 equiv.), DIPEA (6 equiv.), CH_2_Cl_2_, 0 °C, N_2_, 20 h (yields 24–72%) or carboxylic acid RCO_2_H (**9**, **11**) (2.5 equiv.), Boc-protected polyamine (**16a**–**f**) (1 equiv.), with EDC·HCl (2.8 equiv.), DMAP (5 equiv.), CH_2_Cl_2_, 0 °C, N_2_, 12 h (yields 40–95%); (ii) TFA (0.2 mL), CH_2_Cl_2_ (2 mL), r.t., 2 h (yields 30–97%).

**Table 1:** Antimicrobial activities (MIC, µM) and cLogP values of analogues **17**–**21**.

Cmpd	cLogP ^a^	S. a ^b^	MRSA ^c^	P. a ^d^	E. c ^e^	K. p ^f^	A. b ^g^	C. a ^h^	C. n ^i^
**17a**	5.1	677	>43.3	677	677	>43.3	>43.3	>43.3	>43.3
**17b**	6.0	200	>41.7	200	200	>41.7	>41.7	>41.7	>41.7
**17c**	6.5	256	>41.0	>256	256	>41.0	>41.0	>41.0	>41.0
**17d**	6.9	50	>40.3	>100	100	>40.3	>40.3	>40.3	>40.3
**17e**	7.8	244	9.72	608	608	>38.9	>38.9	>38.9	38.9
**17f**	8.7	25	0.29	300	100	>37.6	0.29	>37.6	0.29
**18a**	4.8	568	>36.3	568	568	>36.3	>36.3	>36.3	>36.3
**18b**	5.7	>200	>35.2	>200	>200	>35.2	>35.2	>35.2	>35.2
**18c**	6.2	>200	>34.7	200	200	>34.7	>34.7	>34.7	>34.7
**18d**	6.6	107	>34.2	>107	>107	>34.2	>34.2	>34.2	>34.2
**18e**	7.5	104	33.2	518	518	>33.2	>33.2	>33.2	33.2
**18f**	8.4	3.15	≤0.25	>100	12.5	>32	>32	>32	≤0.25
**19a**	5.1	34	43.3	677	271	>43.3	>43.3	>43.3	21.7
**19b**	6.0	65.2	41.7	>130	130	>41.7	>41.7	>41.7	20.9
**19c**	6.5	16.0	≤0.32	32.0	16.0	>41	>41	>41	≤0.32
**19d**	6.9	7.9	40.3	126	126	>40.3	>40.3	>40.3	40.3
**19e**	7.8	30	1.22	608	122	38.9	38.9	38.9	4.86
**19f**	8.7	100	0.294	300	300	>37.6	0.294	>37.6	0.294
**20a**	4.8	57	18.16	568	227	>36.3	>36.3	>36.3	>36.3
**20b**	5.7	>200	>35.2	>200	50	>35.2	>35.2	>35.2	35.2
**20c**	6.2	25	2.2	>200	25	>34.7	>34.7	>34.7	≤0.27
**20d**	6.6	13.3	>34.2	>107	107	>34.2	>34.2	>34.2	17.1
**20e**	7.5	12.95	16.6	518	104	>33.2	>33.2	>33.2	16.6
**20f**	8.4	3.125	≤0.25	>100	6.3	>32	>32	>32	≤0.25
**21a**	5.7	300	0.268	300	300	>34.3	>34.3	>34.3	>34.3
**21b**	6.6	300	2.08	300	300	>33.3	>33.3	>33.3	>33.3
**21c**	7.1	200	0.256	300	300	>32.8	>32.8	>32.8	>32.8
**21d**	7.6	300	0.506	300	300	>32.4	>32.4	>32.4	>32.4
**21e**	8.5	300	0.246	300	300	>31.5	>31.5	31.5	15.73

^a^ cLogP values calculated in DataWarrior v05.05.00 [33]; ^b^ *S. aureus* (ATCC 25,923 or ATCC 29213) with streptomycin (MIC 21.5 µM) and chloramphenicol (MIC 1.5–3 µM) were used as positive controls, and values are presented as the mean (n = 3); ^c^ MRSA (ATCC 43300) with vancomycin (MIC 0.7 µM) was used as a positive control, and values are presented as the mean (n = 2); ^d^ *P. aeruginosa (*PAO1 or ATCC 27853) with streptomycin (MIC 21.5 µM) and colistin (MIC 1 μM) were used as positive controls, and values are presented as the mean (n = 3); ^e^ *E. coli* (ATCC 25922) with colistin (MIC 2 µM) was used as a positive control, and values are presented as the mean (n = 2); ^f^ *K. pneumoniae* (ATCC 700603) with colistin (MIC 0.2 µM) was used as a positive control, and values are presented as the mean (n = 2); ^g^ *A. baumannii* (ATCC 19606) with colistin (MIC 0.2 µM) was used as a positive control, and values are presented as the mean (n = 2); ^h^ *C. albicans* (ATCC 90028) with fluconazole (MIC 0.4 µM) was used as a positive control, and values are presented as the mean (n = 2); ^i^ *C. neoformans* (ATCC 208821) with fluconazole (MIC 26 µM) was used as a positive control, and values are presented as the mean (n = 2).

**Table 2:** Cytotoxic (IC_50_, µM) and hemolytic (HC_10_, µM) properties of analogues **17**–**21**.

Compound	Cytotoxicity ^a^	Hemolysis ^b^	Compound	Cytotoxicity ^a^	Hemolysis ^b^
**17a**	nt ^c^	nt ^c^	20a	>36.3	>36.3
**17b**	>41.7	>41.7	20b	>35.2	>35.2
**17c**	>41.0	>41.0	20c	>34.7	>34.7
**17d**	>40.3	>40.3	20d	>34.2	>34.2
**17e**	23.7	>38.9	20e	>33.2	>33.2
**17f**	>37.6	>37.6	20f	>32.2	>32.2
**18a**	nt ^c^	nt ^c^	21a	>34.3	31.4
**18b**	>35.2	>35.2	21b	>33.3	>33.3
**18c**	>34.7	>34.7	21c	>32.8	>32.8
**18d**	>34.2	>34.2	21d	>32.4	>32.4
**18e**	>33.2	>33.2	21e	>31.5	6.32
**18f**	>32.2	>32.2			
**19a**	26.4	>43.3			
**19b**	41.7	>41.7			
**19c**	20.5	>41.0			
**19d**	40.3	>40.3			
**19e**	4.75	>38.9			
**19f**	>37.6	>37.6			

All values presented as the mean (n = 2); ^a^ Concentration (IC_50_, µM) of compound at 50% cytotoxicity on HEK293 human embryonic kidney cells with tamoxifen as the positive control (IC_50_ 24 μM); ^b^ Concentration (HC_10_, µM) of compound at 10% hemolytic activity on human red blood cells with melittin as the positive control (HC_10_ 0.95 µM); ^c^ Not tested.

**Table 3:** Antibiotic enhancement activity (MIC μM) of analogues **17**–**21**.

Compound	Dox/P.a ^a^	Eryth/E.c ^b^	Compound	Dox/P.a ^a^	Eryth/E.c ^b^
**17a**	271 (2.5)	67.7 (10)	**20a**	28 (20)	nt ^c^
**17b**	12.5 (16)	100 (2)	**20b**	25 (>8)	50 (1)
**17c**	64 (>2.7)	64 (4)	**20c**	6.25 (>32)	12.5 (2)
**17d**	126 (1)	31.5 (3)	**20d**	53.4 (>2)	26.7 (4)
**17e**	608 (1)	122 (5)	**20e**	104 (5)	13.0 (8)
**17f**	300 (1)	50 (2)	**20f**	12.6 (>8)	3.15 (2)
**18a**	568 (1)	568 (1)	**21a**	300 (1)	300 (1)
**18b**	100 (>2)	200 (1)	**21b**	300 (1)	300 (1)
**18c**	12.5 (16)	200 (1)	**21c**	300 (1)	300 (1)
**18d**	>107 (1)	107 (1)	**21d**	300 (1)	300 (1)
**18e**	207 (2.5)	51.8 (10)	**21e**	300 (1)	300 (1)
**18f**	50.3 (>2)	6.29 (2)			
**19a**	16.9 (40)	4.2 (64)			
**19b**	65.2 (>2)	65.2 (2)			
**19c**	4.00 (8)	4.0 (4)			
**19d**	15.7 (8)	15.7 (8)			
**19e**	608 (1)	60.8 (2)			
**19f**	300 (1)	300 (1)			

^a^ Concentration (µM) required to restore doxycycline activity at 4.5 µM against *P. aeruginosa* (ATCC 27853). Fold change shown in parentheses is the ratio between the intrinsic MIC of the test compound and the combination MIC; ^b^ Concentration (µM) required to restore erythromycin activity at 10.9 µM against *E. coli* (ATCC 25922). Fold change shown in parentheses is the ratio between the intrinsic MIC of the test compound and the combination MIC; ^c^ Not tested.

**Table 4:** Dose response enhancement (MIC, µM) of doxycycline towards *P. aeruginosa* (ATCC 27853) by compounds **19a** and **20a**.

	Compound	
Doxycycline Concentration (μg/mL)	19a ^a^	20a ^a^
2	16.9	28.4
4	8.5	14.2
6	8.5	14.2
8	4.2	3.5

^a^ Concentration of test compound (µM) required to restore doxycycline activity at the dose specified against *P. aeruginosa* (ATCC 27853).

## Data Availability

Data are contained within the article or Supplementary Materials.

## Supplementary Materials

The following supporting information can be downloaded at: https://www.mdpi.com/article/10.3390/antibiotics12061014/s1, Figure S1 ^1^H NMR (DMSO-*d*_6_, 400 MHz) and ^13^C NMR (DMSO-*d*_6_, 100 MHz) spectra for **9**; Figure S2 ^1^H NMR (DMSO-*d*_6_, 400 MHz) and ^13^C NMR (DMSO-*d*_6_, 100 MHz) spectra for **11**; Figure S3 ^1^H NMR (DMSO-*d*_6_, 400 MHz) and ^13^C NMR (DMSO-*d*_6_, 100 MHz) spectra for **12**; Figure S4 ^1^H NMR (DMSO-*d*_6_, 400 MHz) and ^13^C NMR (DMSO-*d*_6_, 100 MHz) spectra for **17a**; Figure S5 ^1^H NMR (CD_3_OD, 400 MHz) and ^13^C NMR (CD_3_OD, 100 MHz) spectra for **17b**; Figure S6 ^1^H NMR (CD_3_OD, 400 MHz) and ^13^C NMR (CD_3_OD, 100 MHz) spectra for **17c**; Figure S7 ^1^H NMR (CD_3_OD, 400 MHz) and ^13^C NMR (CD_3_OD, 100 MHz) spectra for **17d**; Figure S8 ^1^H NMR (CD_3_OD, 400 MHz) and ^13^C NMR (CD_3_OD, 100 MHz) spectra for **17e**; Figure S9 ^1^H NMR (CD_3_OD, 400 MHz) and ^13^C NMR (CD_3_OD, 100 MHz) spectra for **17f**; Figure S10 ^1^H NMR (CD_3_OD, 400 MHz) and ^13^C NMR (CD_3_OD, 100 MHz) spectra for **18a**; Figure S11 ^1^H NMR (CD_3_OD, 400 MHz) and ^13^C NMR (CD_3_OD, 100 MHz) spectra for **18b**; Figure S12 ^1^H NMR (CD_3_OD, 400 MHz) and ^13^C NMR (CD_3_OD, 100 MHz) spectra for **18c**; Figure S13 ^1^H NMR (CD_3_OD, 400 MHz) and ^13^C NMR (CD_3_OD, 100 MHz) spectra for **18d**; Figure S14 ^1^H NMR (CD_3_OD, 400 MHz) and ^13^C NMR (CD_3_OD, 100 MHz) spectra for **18e**; Figure S15 ^1^H NMR (CD_3_OD, 400 MHz) and ^13^C NMR (CD_3_OD, 100 MHz) spectra for **18f**; Figure S16 ^1^H NMR (CD_3_OD, 400 MHz) and ^13^C NMR (CD_3_OD, 100 MHz) spectra for **19a**; Figure S17 ^1^H NMR (CD_3_OD, 400 MHz) and ^13^C NMR (CD_3_OD, 100 MHz) spectra for **19b**; Figure S18 ^1^H NMR (CD_3_OD, 400 MHz) and ^13^C NMR (CD_3_OD, 100 MHz) spectra for **19c**; Figure S19 ^1^H NMR (CD_3_OD, 400 MHz) and ^13^C NMR (CD_3_OD, 100 MHz) spectra for **19d**; Figure S20 ^1^H NMR (CD_3_OD, 400 MHz) and ^13^C NMR (CD_3_OD, 100 MHz) spectra for **19e**; Figure S21 ^1^H NMR (CD_3_OD, 400 MHz) and ^13^C NMR (CD_3_OD, 100 MHz) spectra for **19f**; Figure S22 ^1^H NMR (CD_3_OD, 400 MHz) and ^13^C NMR (CD_3_OD, 100 MHz) spectra for **20a**; Figure S23 ^1^H NMR (CD_3_OD, 400 MHz) and ^13^C NMR (CD_3_OD, 100 MHz) spectra for **20b**; Figure S24 ^1^H NMR (CD_3_OD, 400 MHz) and ^13^C NMR (CD_3_OD, 100 MHz) spectra for **20c**; Figure S25 ^1^H NMR (CD_3_OD, 400 MHz) and ^13^C NMR (CD_3_OD, 100 MHz) spectra for **20d**; Figure S26 ^1^H NMR (CD_3_OD, 400 MHz) and ^13^C NMR (CD_3_OD, 100 MHz) spectra for **20e**; Figure S27 ^1^H NMR (CD_3_OD, 400 MHz) and ^13^C NMR (CD_3_OD, 100 MHz) spectra for **20f**; Figure S28 ^1^H NMR (DMSO-*d*_6_, 400 MHz) and ^13^C NMR (DMSO-*d*_6_, 100 MHz) spectra for **21a**; Figure S29 ^1^H NMR (DMSO-*d*_6_, 400 MHz) and ^13^C NMR (DMSO-*d*_6_, 100 MHz) spectra for **21b**; Figure S30 ^1^H NMR (DMSO-*d*_6_, 400 MHz) and ^13^C NMR (DMSO-*d*_6_, 100 MHz) spectra for **21c**; Figure S31 ^1^H NMR (DMSO-*d*_6_, 400 MHz) and ^13^C NMR (DMSO-*d*_6_, 100 MHz) spectra for **21d**; Figure S32 ^1^H NMR (DMSO-*d*_6_, 400 MHz) and ^13^C NMR (DMSO-*d*_6_, 100 MHz) spectra for **21e**.
