# Supplementary material for: Investigation of Naphthyl–Polyamine Conjugates as Antimicrobials and Antibiotic Enhancers
Source: Antibiotics (Basel). 2023 Jun 5;12(6):1014. doi: 10.3390/antibiotics12061014 (PMC10294832; doi:10.3390/antibiotics12061014)
Supplement: Supplementary file 1 [file antibiotics-12-01014-s001.zip › antibiotics-2426559-supplementary.pdf]

Supporting Information

Investigation of Naphthyl-Polyamine Conjugates as Antimicrobials and Antibiotic Enhancers

Melissa M. Cadelis [1,2], Liam R. Edmeades [1], Dan Chen [1], Evangelene S. Gill [1], Kyle Fraser [1], Florent Rouvier [3], Marie-Lise Bourguet-Kondracki [4], Jean Michel Brunel [3] and Brent R. Copp[1,*]

[1] School of Chemical Sciences, The University of Auckland, Private Bag 92019, Auckland 1142, New Zealand
[2] School of Medical Sciences, The University of Auckland, Private Bag 92019, Auckland 1142, New Zealand
[3] Membranes et Cibles Thérapeutiques (MCT), SSA, INSERM, Aix-Marseille Universite, 27 bd Jean Moulin, 13385 Marseille, France
[4] Laboratoire Molécules de Communication et Adaptation des Micro-organismes, UMR 7245 CNRS, Muséum National d'Histoire Naturelle, 57 rue Cuvier (C.P. 54), 75005 Paris, France
* Correspondence: b.copp@auckland.ac.nz

Contents

Figure S1 ^{1}H NMR (DMSO-d_6, 400 MHz) and ^{13}C NMR (DMSO-d_6, 100 MHz) spectra for **9** **S3**

Figure S2 ^{1}H NMR (DMSO-d_6, 400 MHz) and ^{13}C NMR (DMSO-d_6, 100 MHz) spectra for **11** **S4**

Figure S3 ^{1}H NMR (DMSO-d_6, 400 MHz) and ^{13}C NMR (DMSO-d_6, 100 MHz) spectra for **12** **S5**

Figure S4 ^{1}H NMR (DMSO-d_6, 400 MHz) and ^{13}C NMR (DMSO-d_6, 100 MHz) spectra for **17a** **S6**

Figure S5 ^{1}H NMR (CD_3OD, 400 MHz) and ^{13}C NMR (CD_3OD, 100 MHz) spectra for **17b** **S7**

Figure S6 ^{1}H NMR (CD_3OD, 400 MHz) and ^{13}C NMR (CD_3OD, 100 MHz) spectra for **17c** **S8**

Figure S7 ^{1}H NMR (CD_3OD, 400 MHz) and ^{13}C NMR (CD_3OD, 100 MHz) spectra for **17d** **S9**

Figure S8 ^{1}H NMR (CD_3OD, 400 MHz) and ^{13}C NMR (CD_3OD, 100 MHz) spectra for **17e** **S10**

Figure S9 ^{1}H NMR (CD_3OD, 400 MHz) and ^{13}C NMR (CD_3OD, 100 MHz) spectra for **17f** **S11**

Figure S10 ^{1}H NMR (CD_3OD, 400 MHz) and ^{13}C NMR (CD_3OD, 100 MHz) spectra for **18a** **S12**

Figure S11 ^{1}H NMR (CD_3OD, 400 MHz) and ^{13}C NMR (CD_3OD, 100 MHz) spectra for **18b** **S13**

Figure S12 ^{1}H NMR (CD_3OD, 400 MHz) and ^{13}C NMR (CD_3OD, 100 MHz) spectra for **18c** **S14**

Figure S13 ^{1}H NMR (CD_3OD, 400 MHz) and ^{13}C NMR (CD_3OD, 100 MHz) spectra for **18d** **S15**

Figure S14 ^{1}H NMR (CD_3OD, 400 MHz) and ^{13}C NMR (CD_3OD, 100 MHz) spectra for **18e** **S16**

Figure S15 ^{1}H NMR (CD_3OD, 400 MHz) and ^{13}C NMR (CD_3OD, 100 MHz) spectra for **18f** **S17**

Figure S16 ^{1}H NMR (CD_3OD, 400 MHz) and ^{13}C NMR (CD_3OD, 100 MHz) spectra for **19a** **S18**

Figure S17 ^{1}H NMR (CD_3OD, 400 MHz) and ^{13}C NMR (CD_3OD, 100 MHz) spectra for **19b** **S19**

Figure S18 ^{1}H NMR (CD_3OD, 400 MHz) and ^{13}C NMR (CD_3OD, 100 MHz) spectra for **19c** **S20**

Figure S19 ^{1}H NMR (CD_3OD, 400 MHz) and ^{13}C NMR (CD_3OD, 100 MHz) spectra for **19d** **S21**

Figure S20 ^{1}H NMR (CD_3OD, 400 MHz) and ^{13}C NMR (CD_3OD, 100 MHz) spectra for **19e** **S22**

Figure S21 ^{1}H NMR (CD_3OD, 400 MHz) and ^{13}C NMR (CD_3OD, 100 MHz) spectra for **19f** **S23**

Figure S22 ^{1}H NMR (CD_3OD, 400 MHz) and ^{13}C NMR (CD_3OD, 100 MHz) spectra for **20a** **S24**

Figure S23 ^{1}H NMR (CD_3OD, 400 MHz) and ^{13}C NMR (CD_3OD, 100 MHz) spectra for **20b** **S25**

Figure S24 ^{1}H NMR (CD_3OD, 400 MHz) and ^{13}C NMR (CD_3OD, 100 MHz) spectra for **20c** **S26**

Figure S25 ^{1}H NMR (CD_3OD, 400 MHz) and ^{13}C NMR (CD_3OD, 100 MHz) spectra for **20d** **S27**

Figure S26 ^{1}H NMR (CD_3OD, 400 MHz) and ^{13}C NMR (CD_3OD, 100 MHz) spectra for **20e** **S28**

Figure S27 ^{1}H NMR (CD_3OD, 400 MHz) and ^{13}C NMR (CD_3OD, 100 MHz) spectra for **20f** **S29**

Figure S28 ^{1}H NMR (DMSO-d_6, 400 MHz) and ^{13}C NMR (DMSO-d_6, 100 MHz) spectra for **21a** **S30**

Figure S29 ^{1}H NMR (DMSO-d_6, 400 MHz) and ^{13}C NMR (DMSO-d_6, 100 MHz) spectra for **21b** **S31**

Figure S30 ^{1}H NMR (DMSO-d_6, 400 MHz) and ^{13}C NMR (DMSO-d_6, 100 MHz) spectra for **21c** **S32**

Figure S31 ^{1}H NMR (DMSO-d_6, 400 MHz) and ^{13}C NMR (DMSO-d_6, 100 MHz) spectra for **21d** **S33**

Figure S32 ^{1}H NMR (DMSO-d_6, 400 MHz) and ^{13}C NMR (DMSO-d_6, 100 MHz) spectra for **21e** **S34**

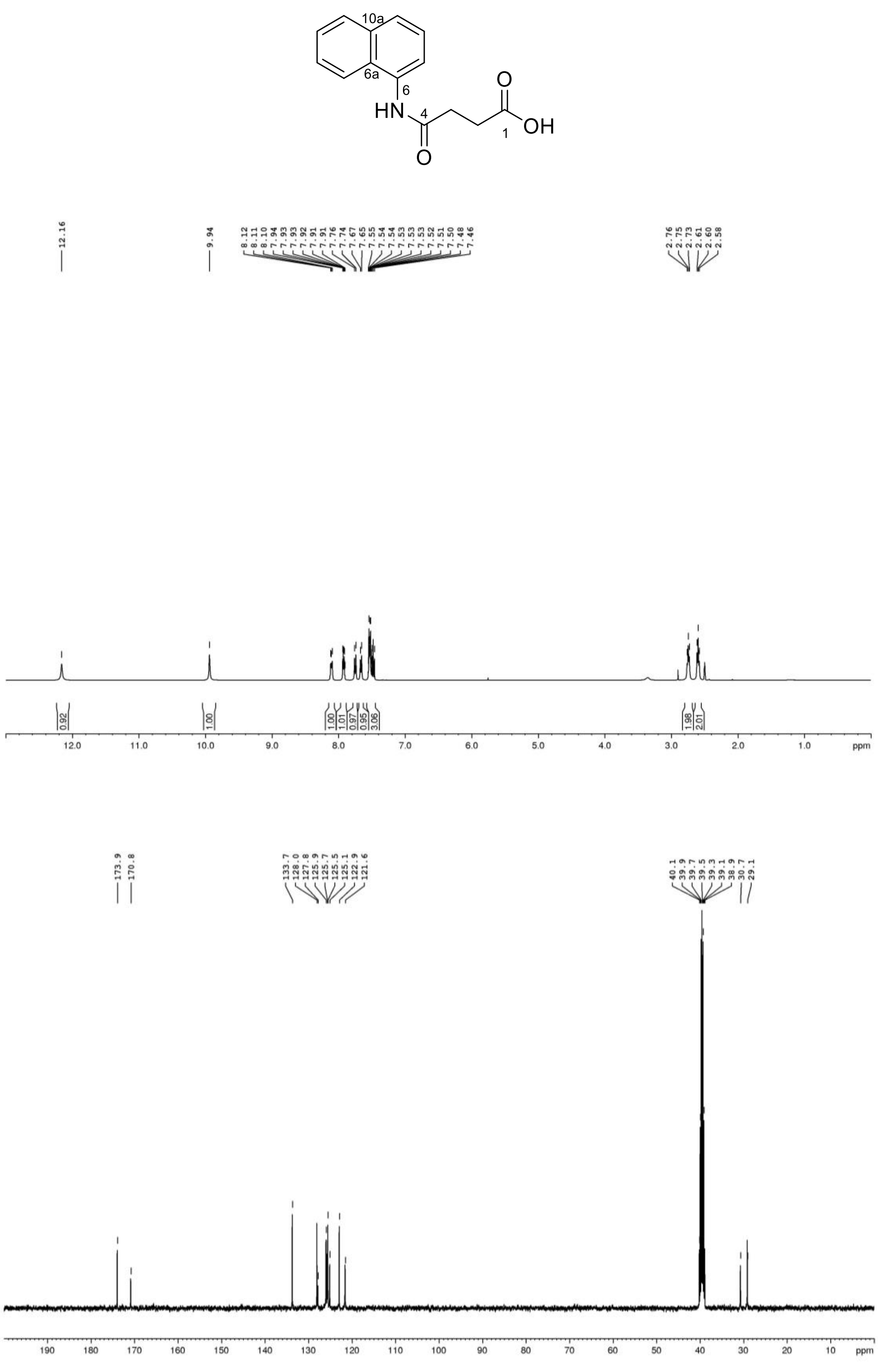

Figure S1 ^{1}H NMR (DMSO-d_6, 400 MHz) and ^{13}C NMR (DMSO-d_6, 100 MHz) spectra for **9**

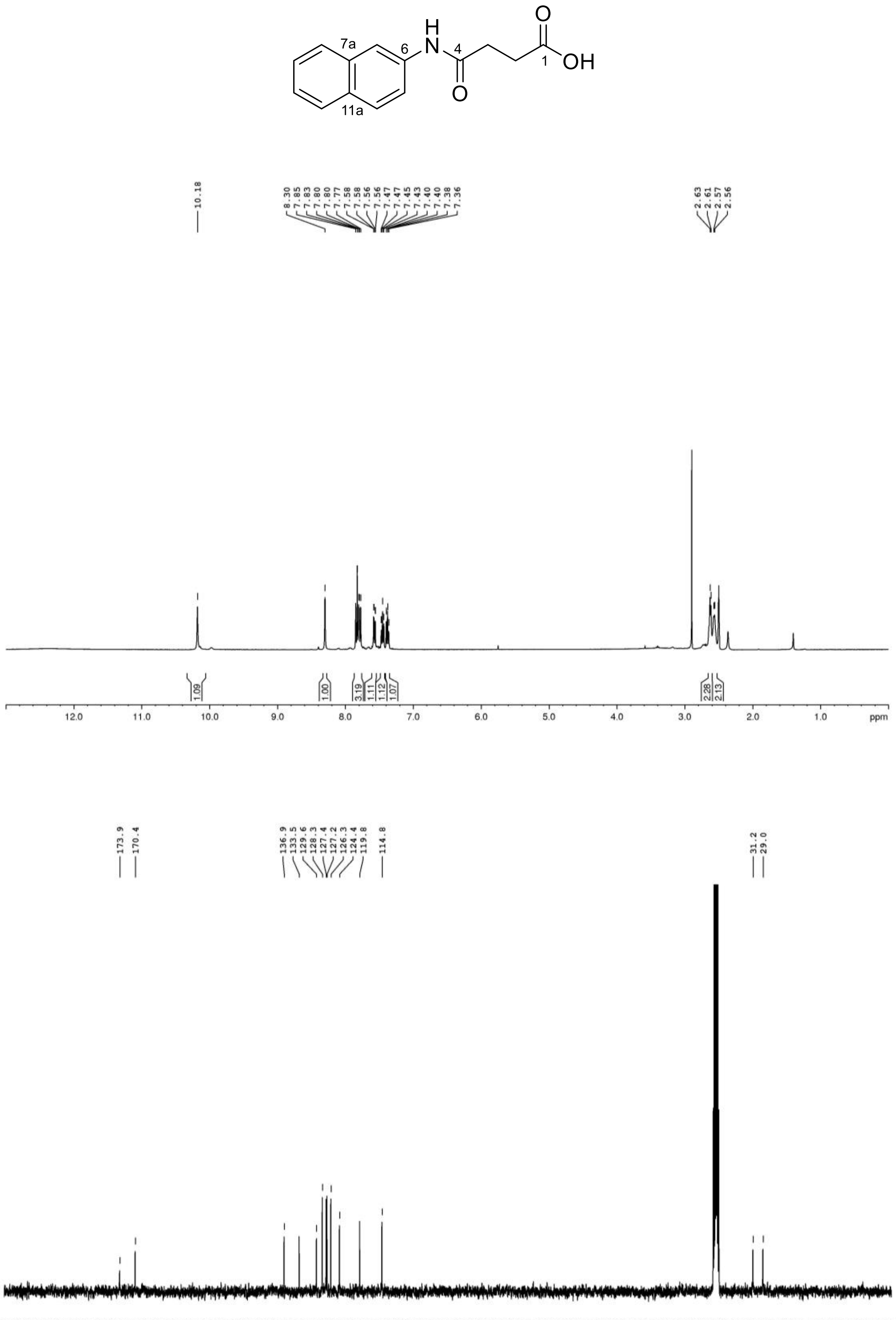

Figure S2 ^{1}H NMR (DMSO-d_6, 400 MHz) and ^{13}C NMR (DMSO-d_6, 100 MHz) spectra for **11**

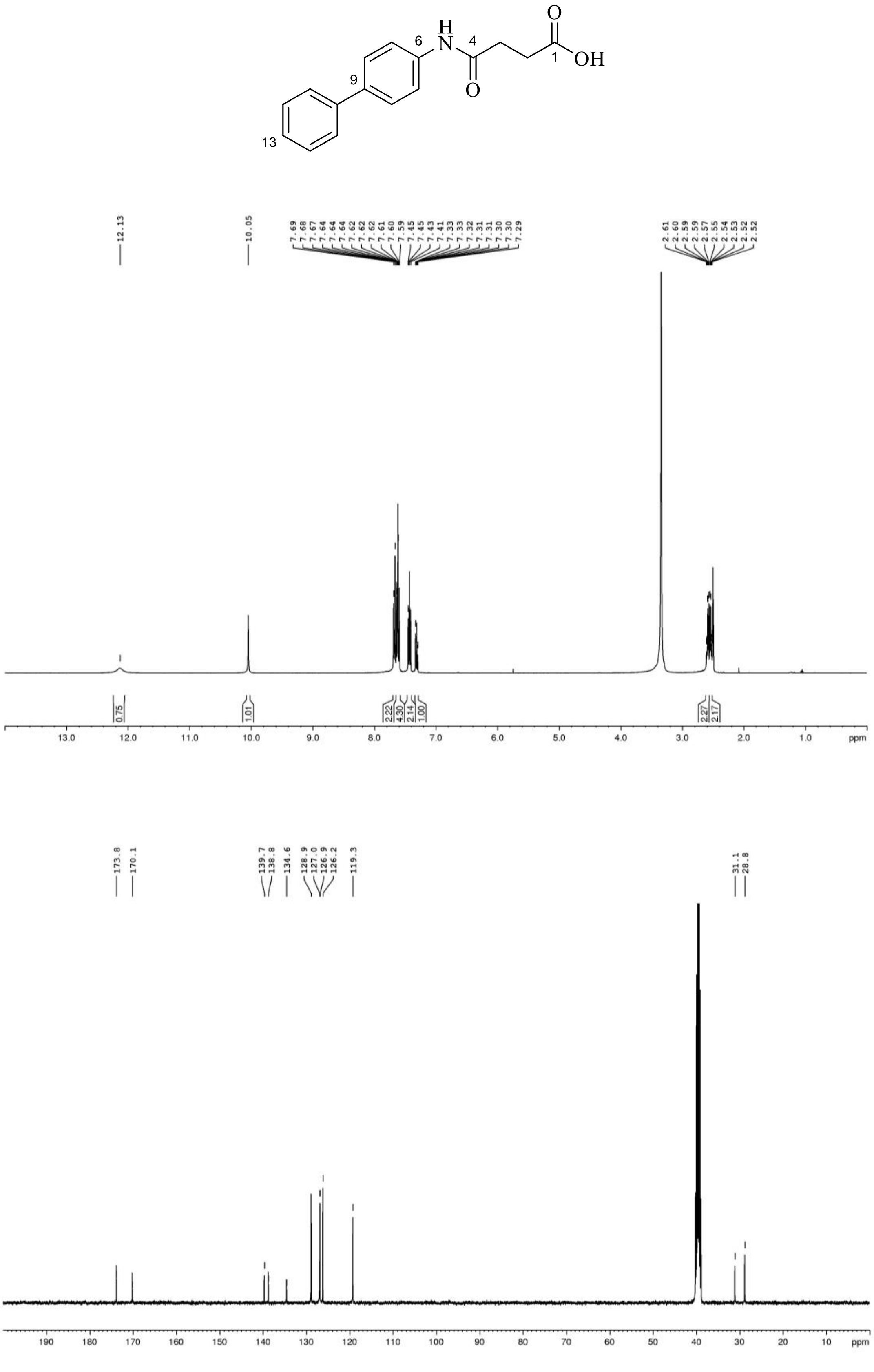

Figure S3 ^{1}H NMR (DMSO-d_6, 400 MHz) and ^{13}C NMR (DMSO-d_6, 100 MHz) spectra for **12**

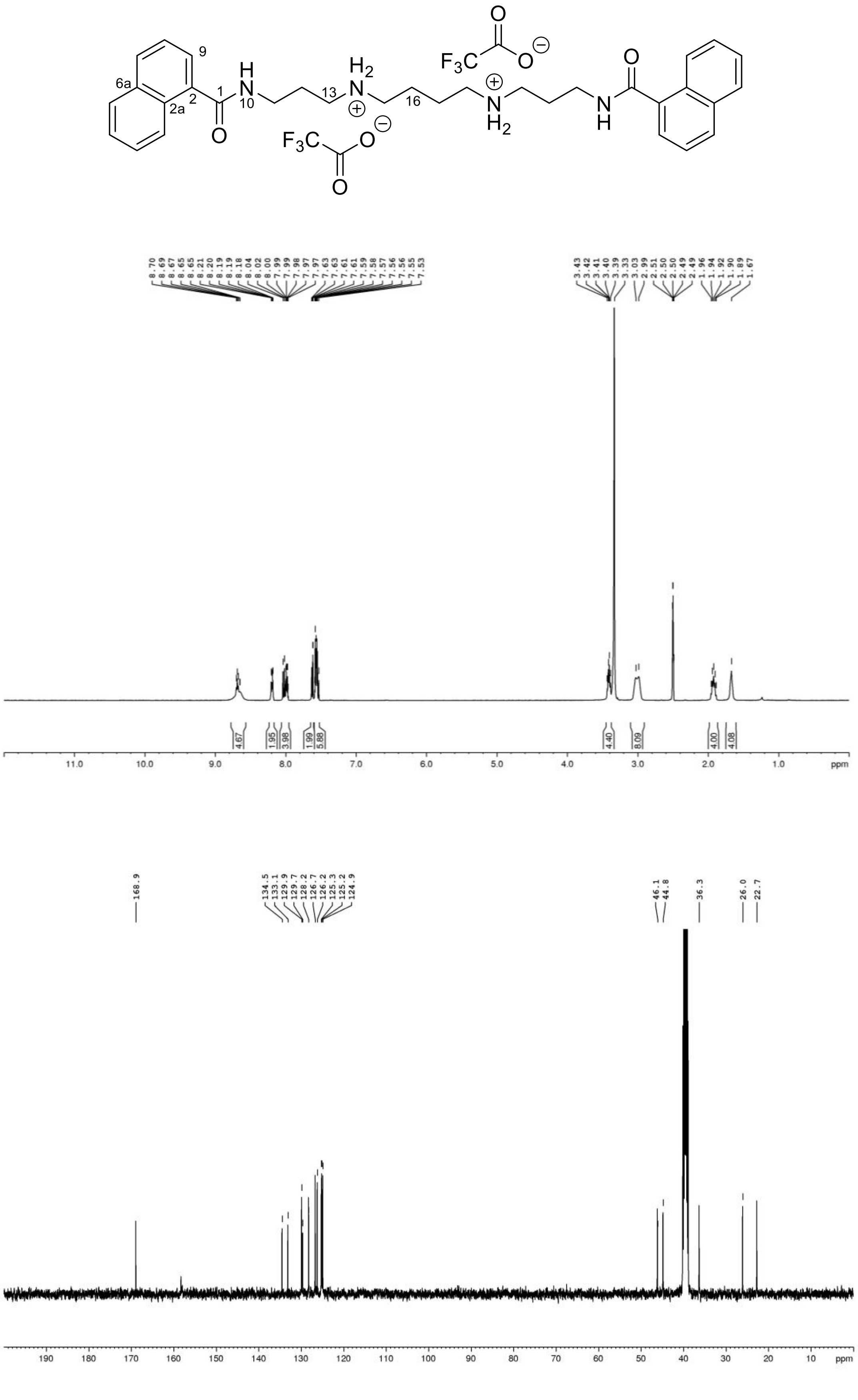

Figure S4 ^{1}H NMR (DMSO-d_6, 400 MHz) and ^{13}C NMR (DMSO-d_6, 100 MHz) spectra for **17a**

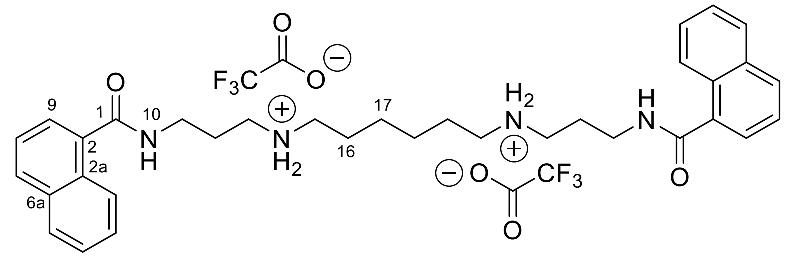

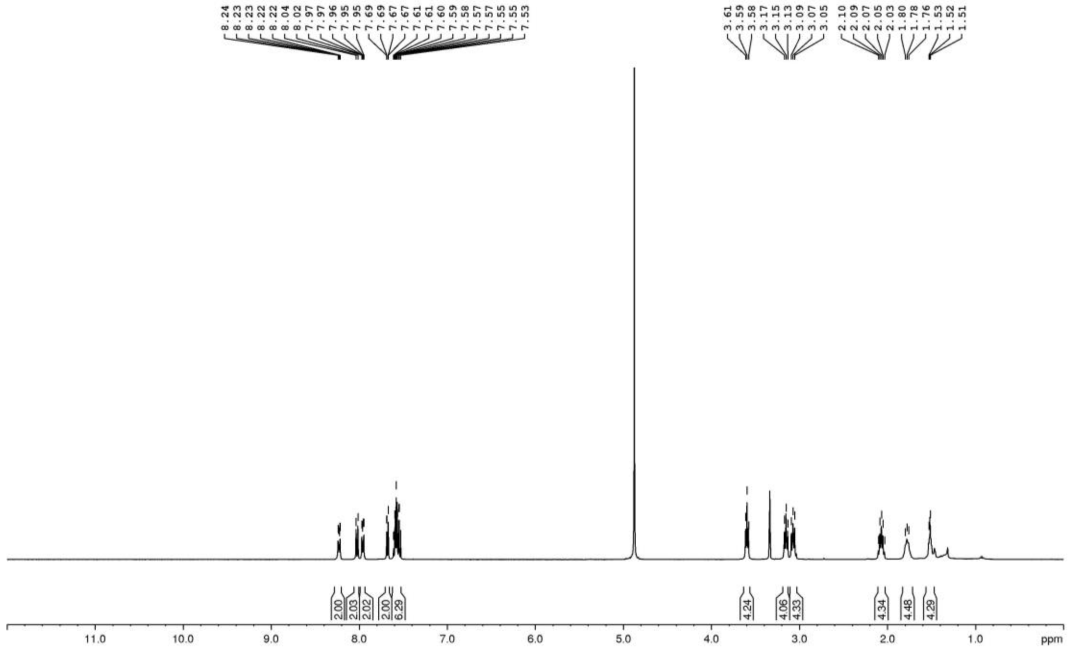

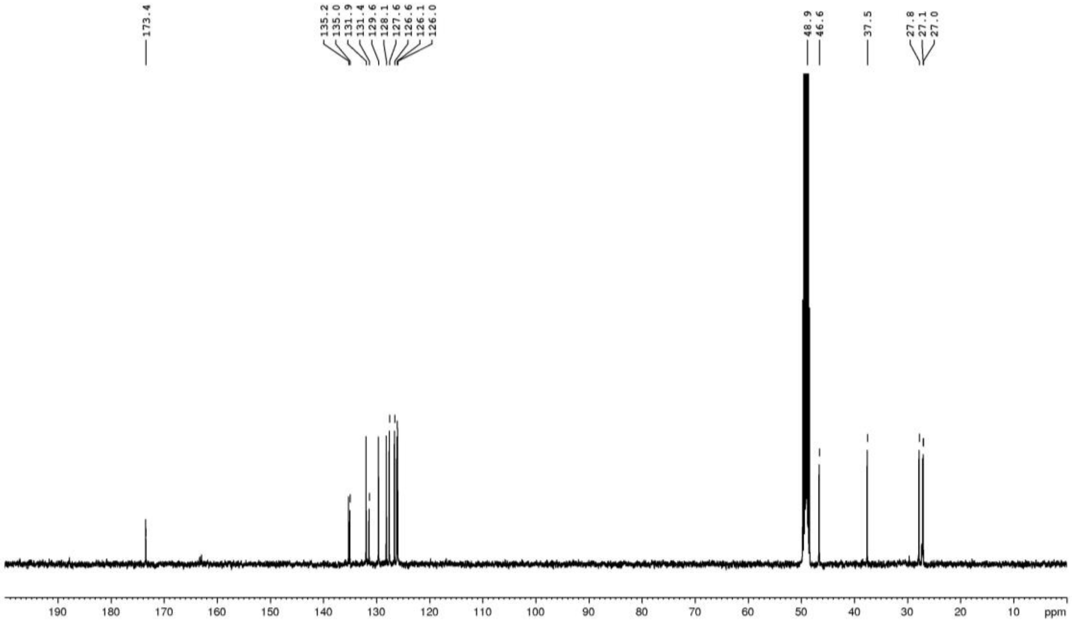

Figure S5 ^{1}H NMR (CD_3OD, 400 MHz) and ^{13}C NMR (CD_3OD, 100 MHz) spectra for **17b**

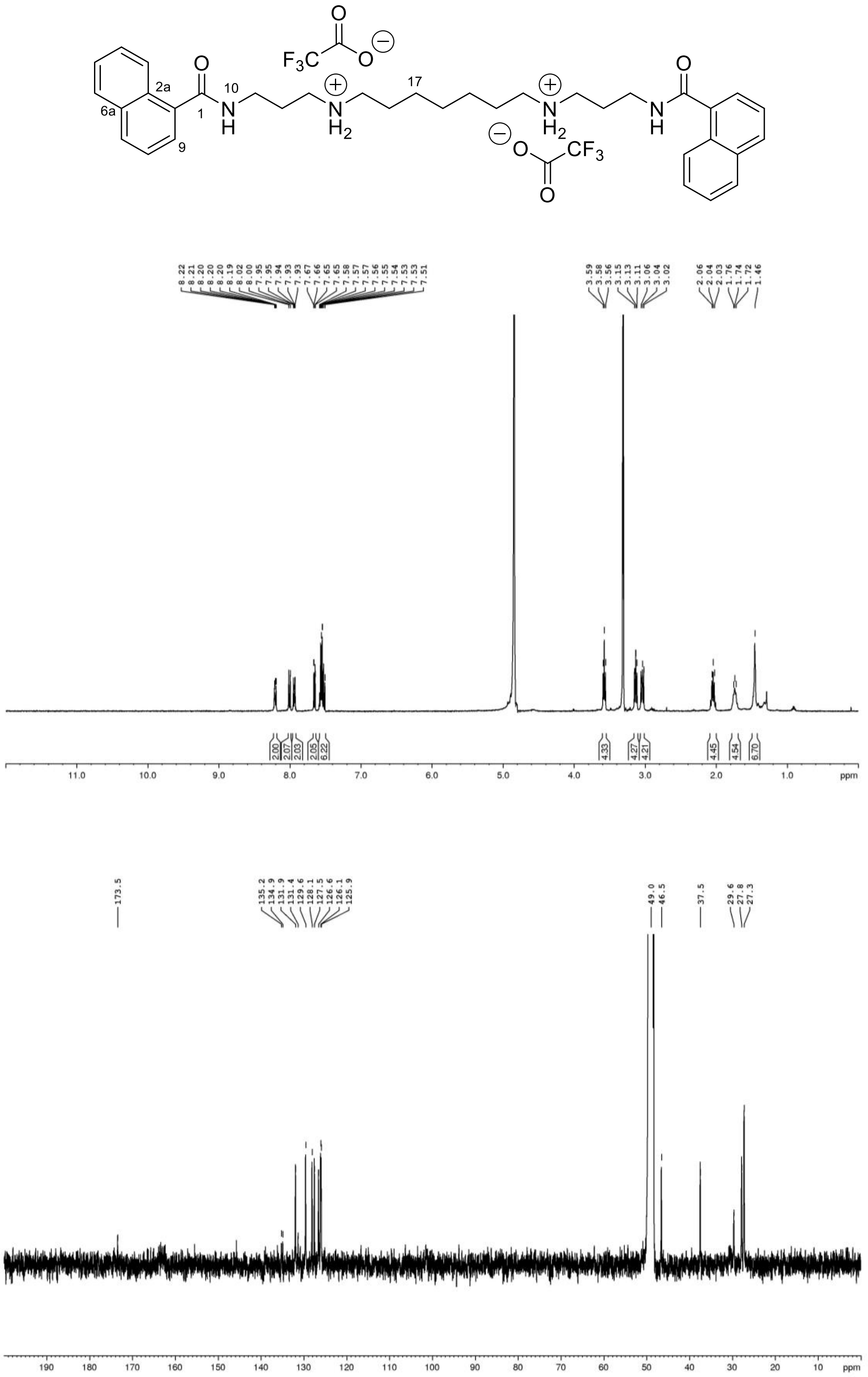

Figure S6 ^{1}H NMR (CD_3OD, 400 MHz) and ^{13}C NMR (CD_3OD, 100 MHz) spectra for **17c**

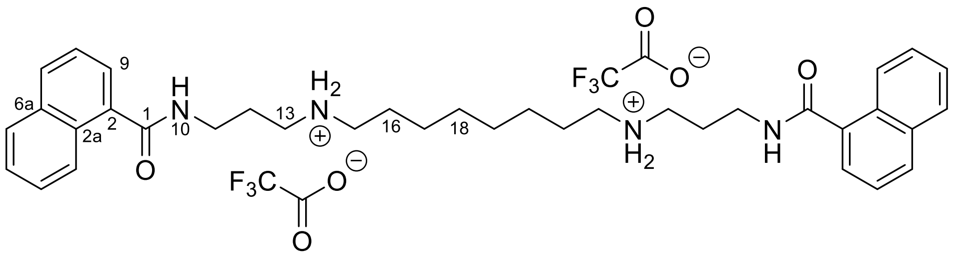

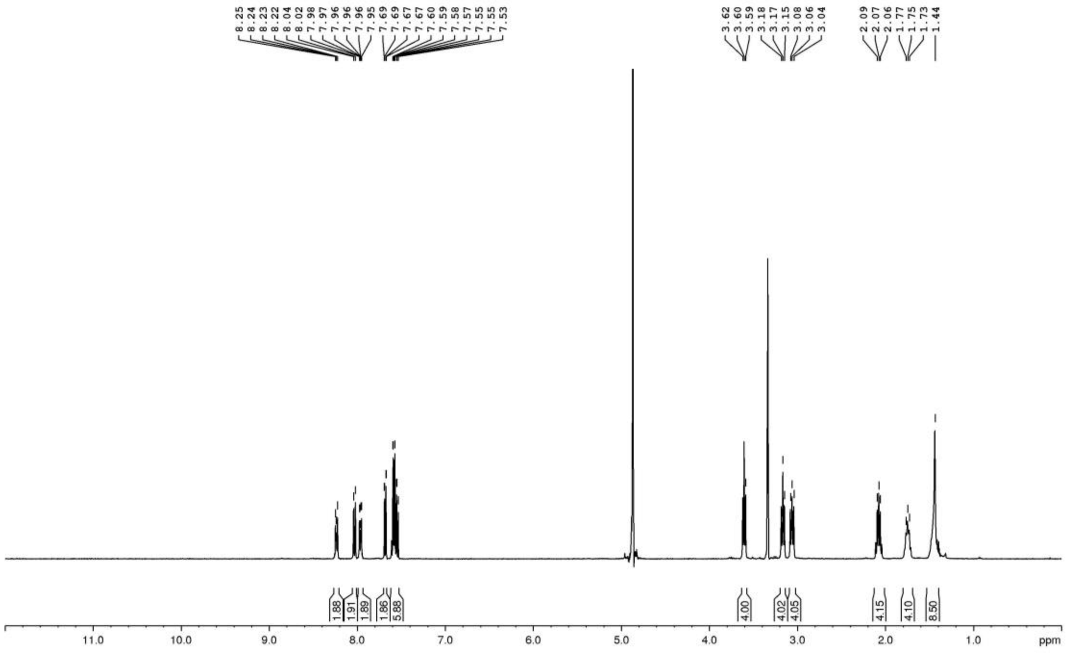

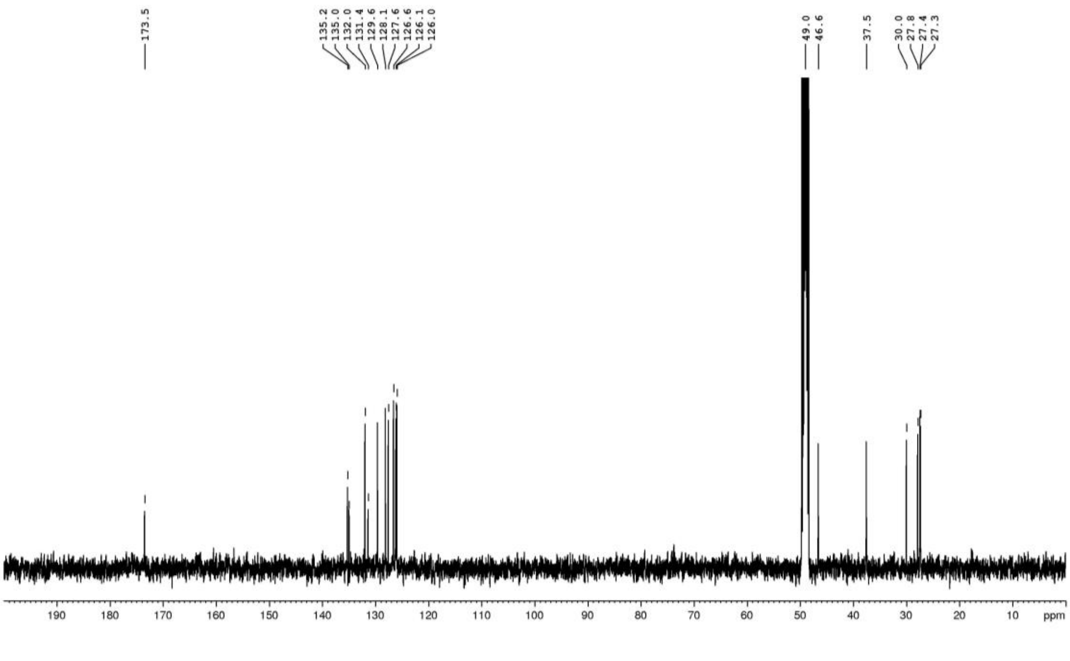

Figure S7 ^{1}H NMR (CD_3OD, 400 MHz) and ^{13}C NMR (CD_3OD, 100 MHz) spectra for **17d**

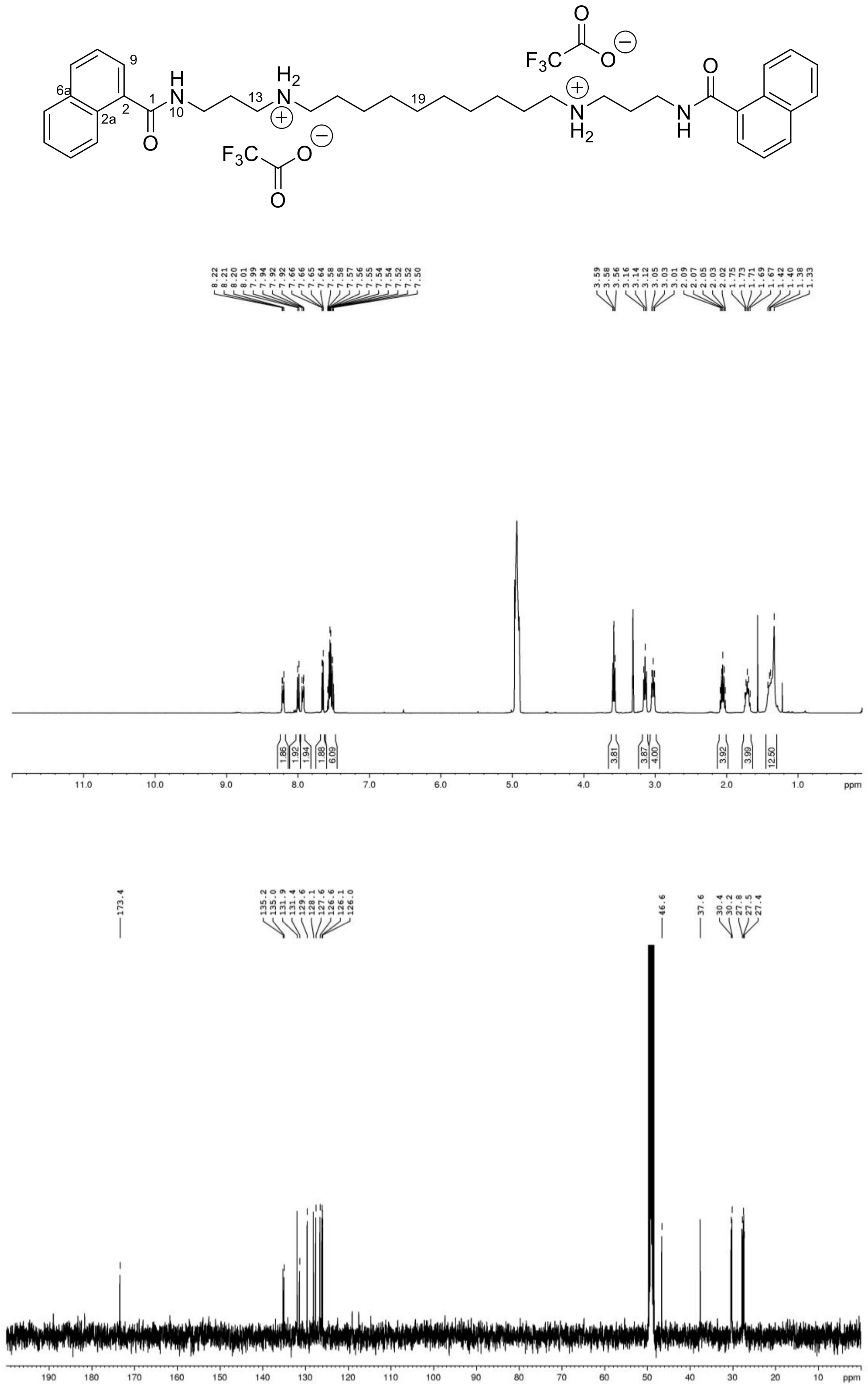

Figure S8 ^{1}H NMR (CD_3OD, 400 MHz) and ^{13}C NMR (CD_3OD, 100 MHz) spectra for **17e**

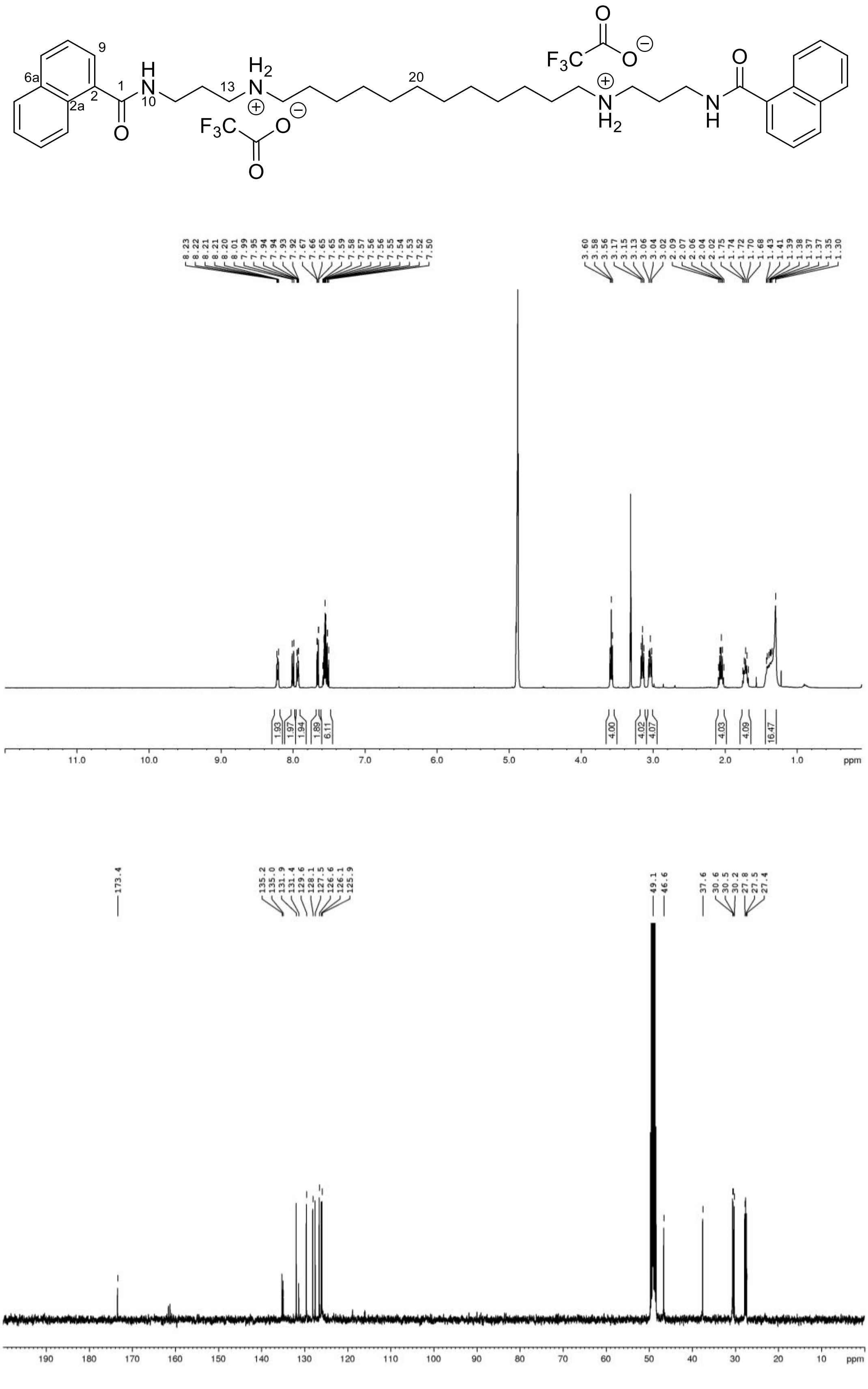

Figure S9 ^{1}H NMR (CD_3OD, 400 MHz) and ^{13}C NMR (CD_3OD, 100 MHz) spectra for **17f**

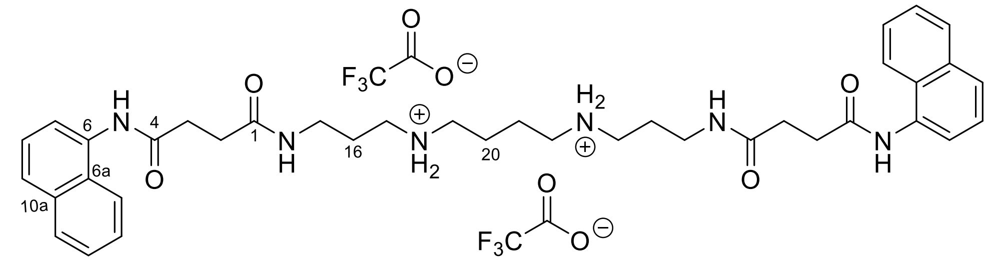

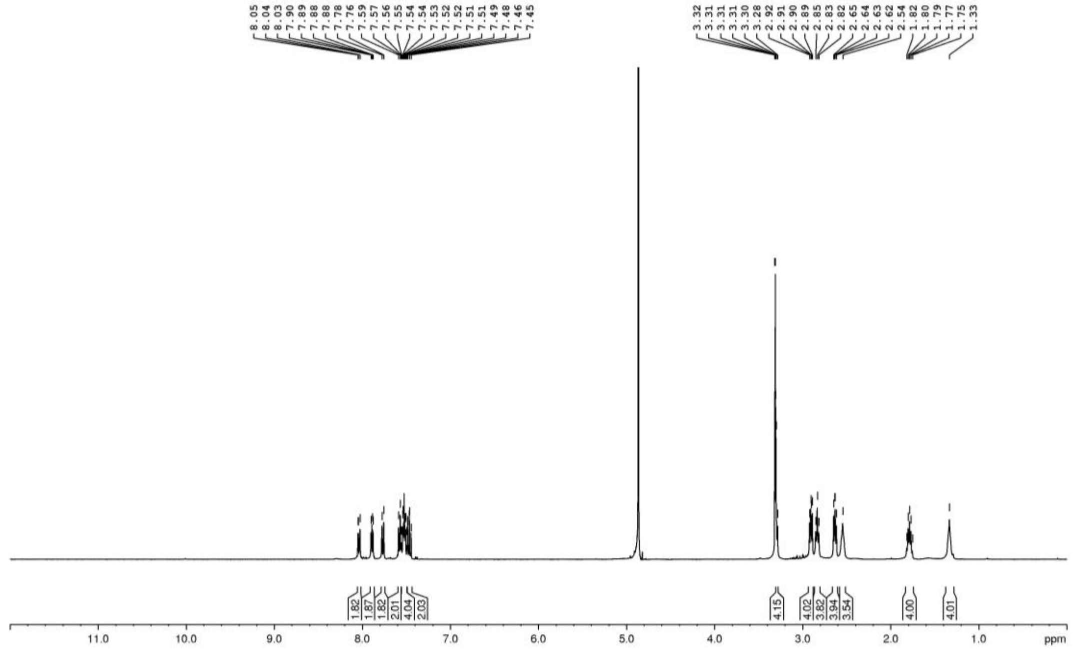

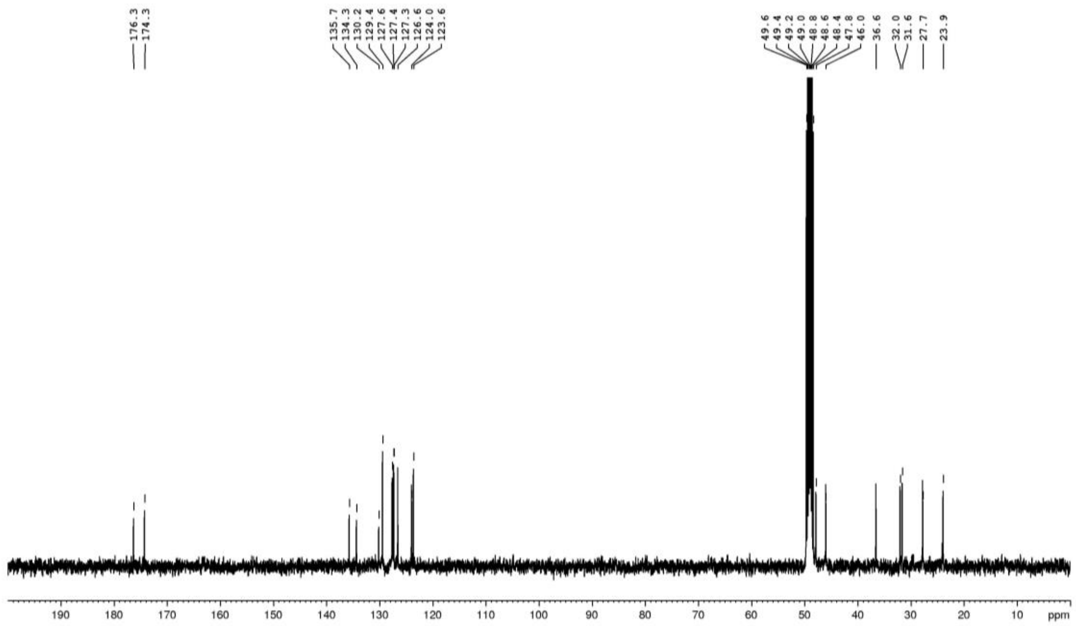

Figure S10 ^{1}H NMR (CD_3OD, 400 MHz) and ^{13}C NMR (CD_3OD, 100 MHz) spectra for **18a**

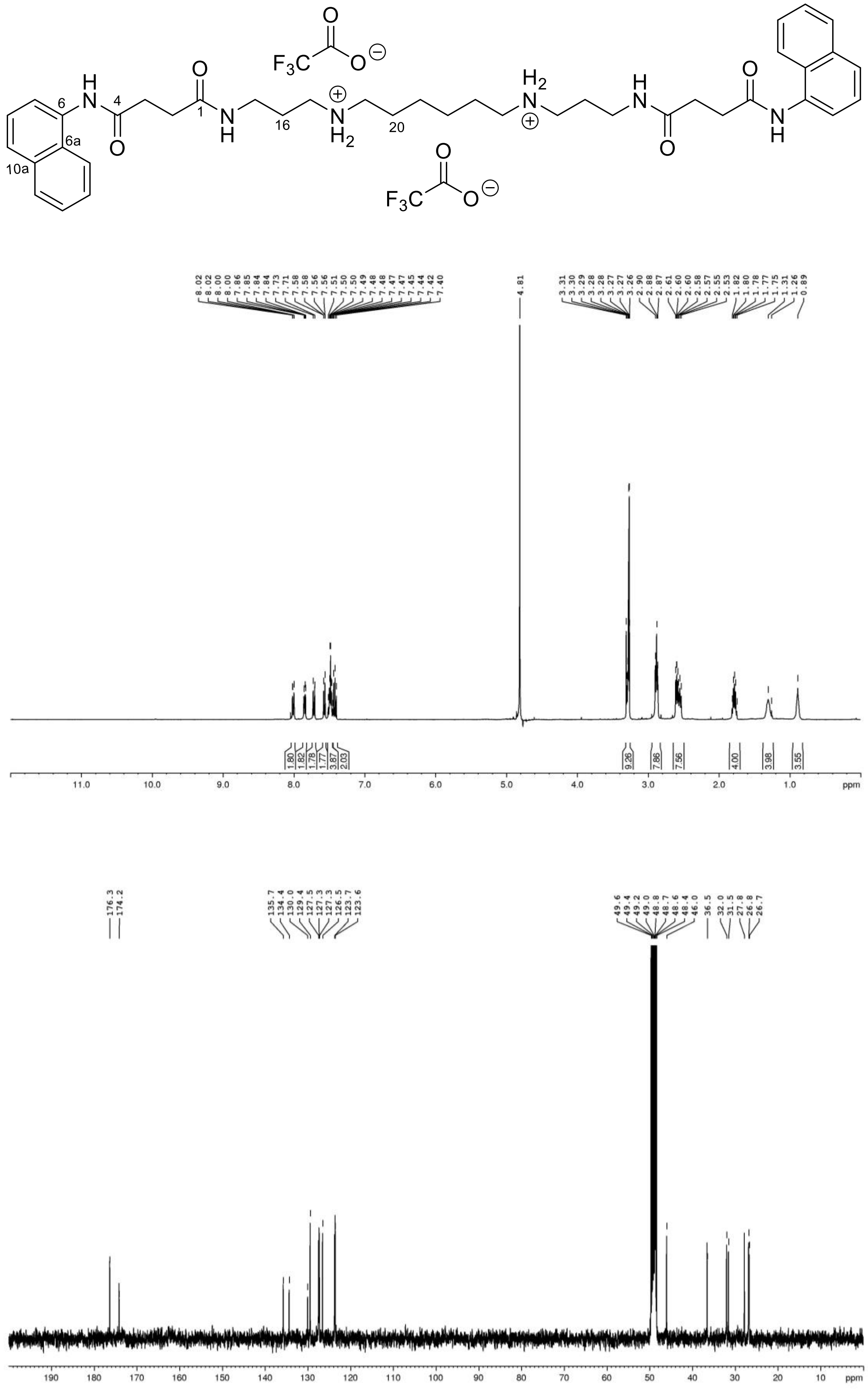

Figure S11 ^{1}H NMR (CD_3OD, 400 MHz) and ^{13}C NMR (CD_3OD, 100 MHz) spectra for **18b**

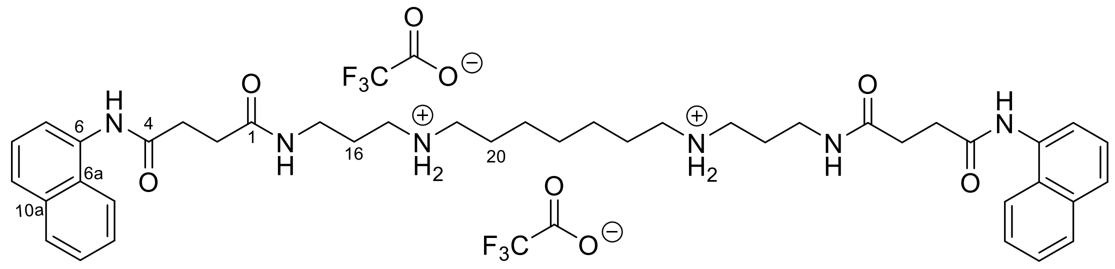

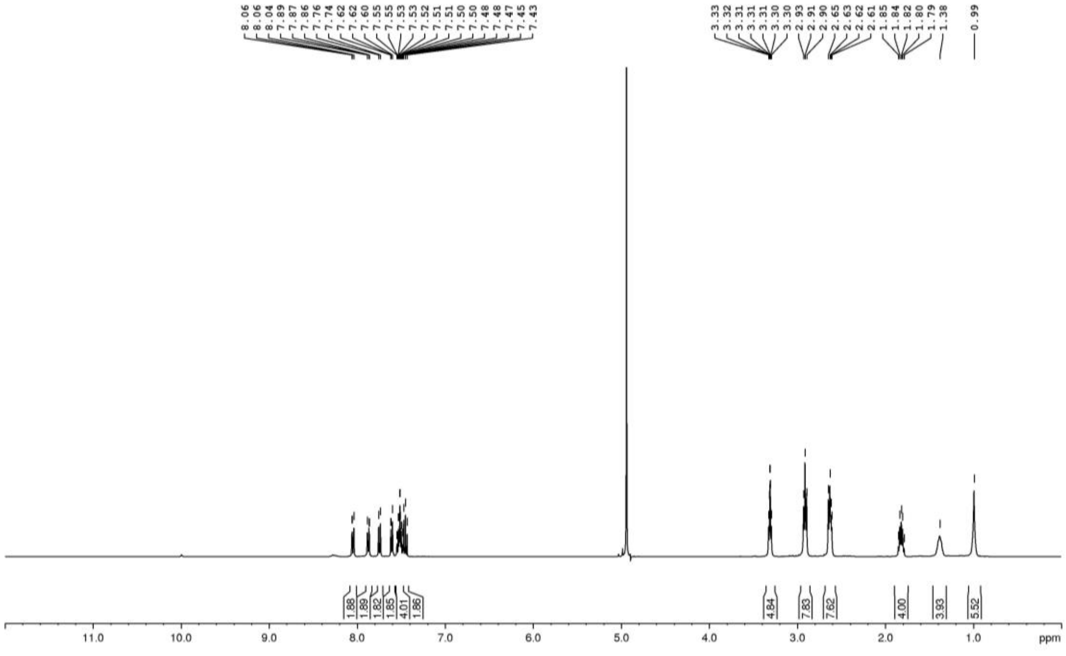

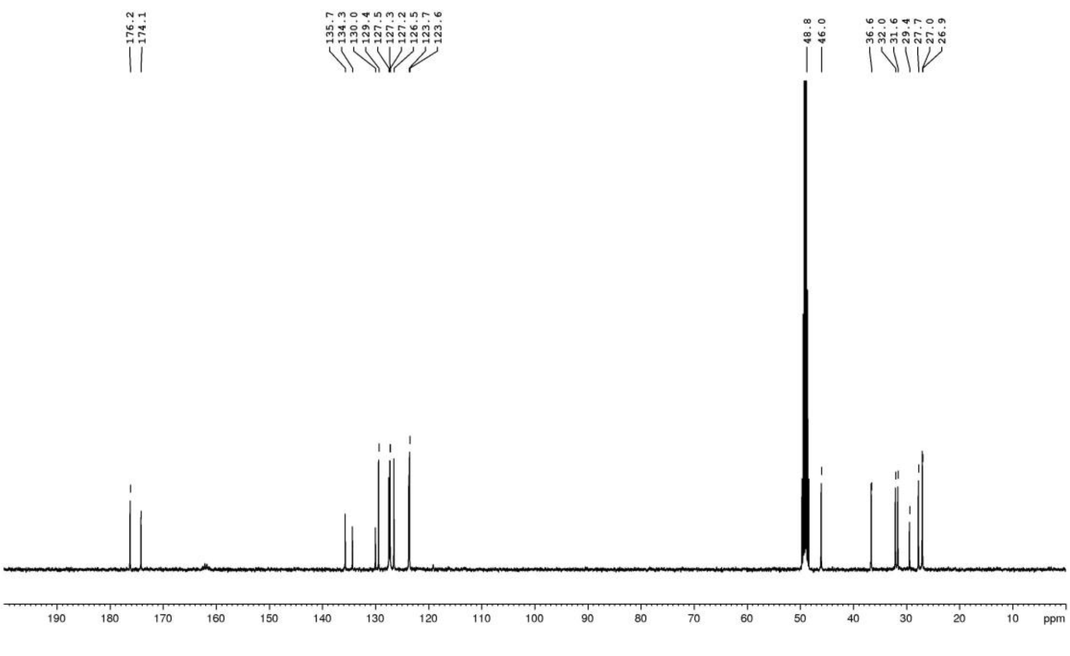

Figure S12 ^{1}H NMR (CD_3OD, 400 MHz) and ^{13}C NMR (CD_3OD, 100 MHz) spectra for **18c**

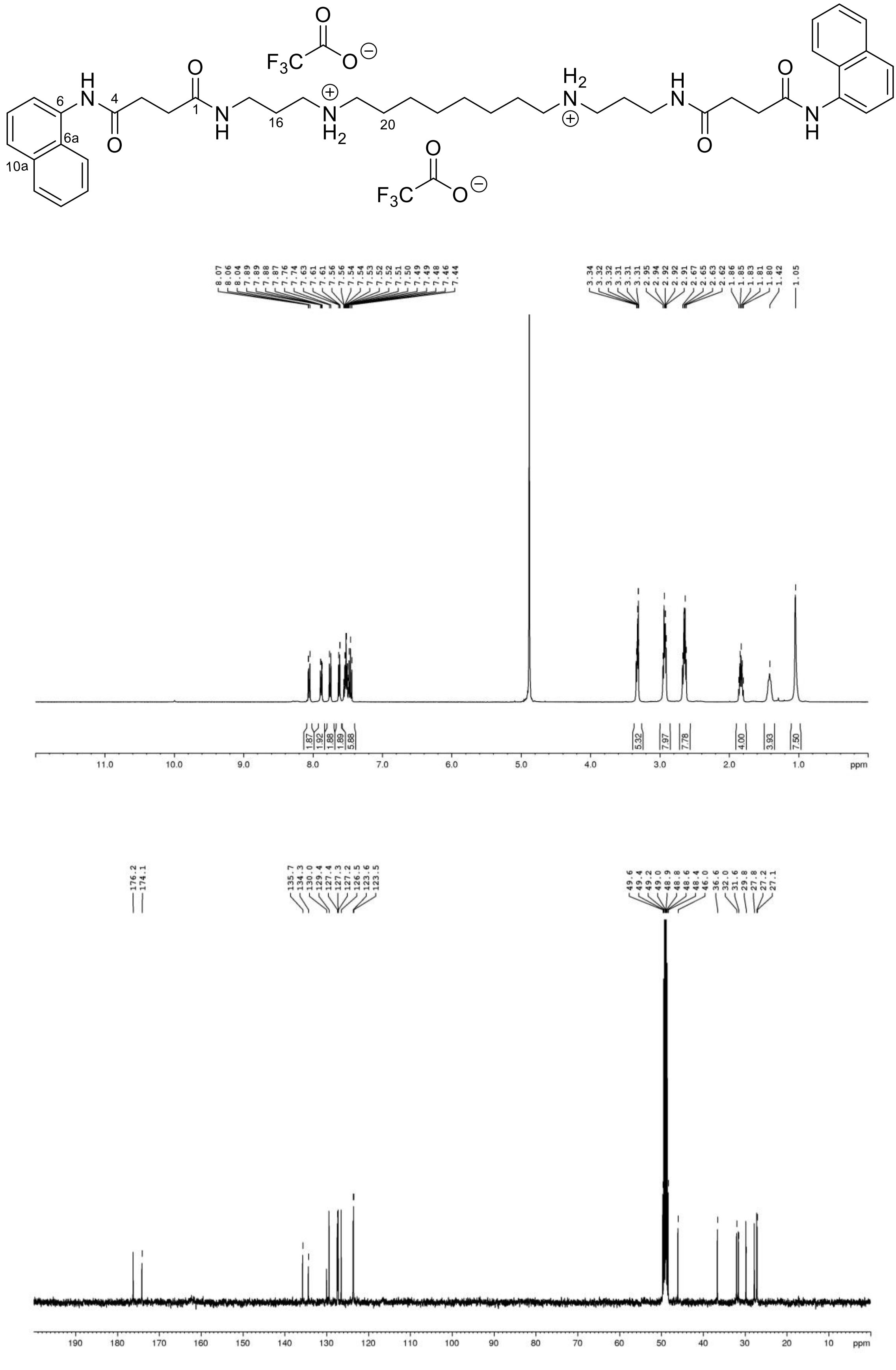

Figure S13 ^{1}H NMR (CD_3OD, 400 MHz) and ^{13}C NMR (CD_3OD, 100 MHz) spectra for **18d**

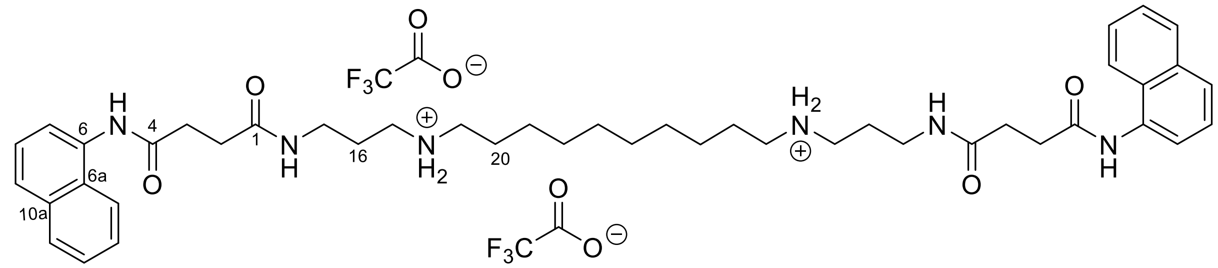

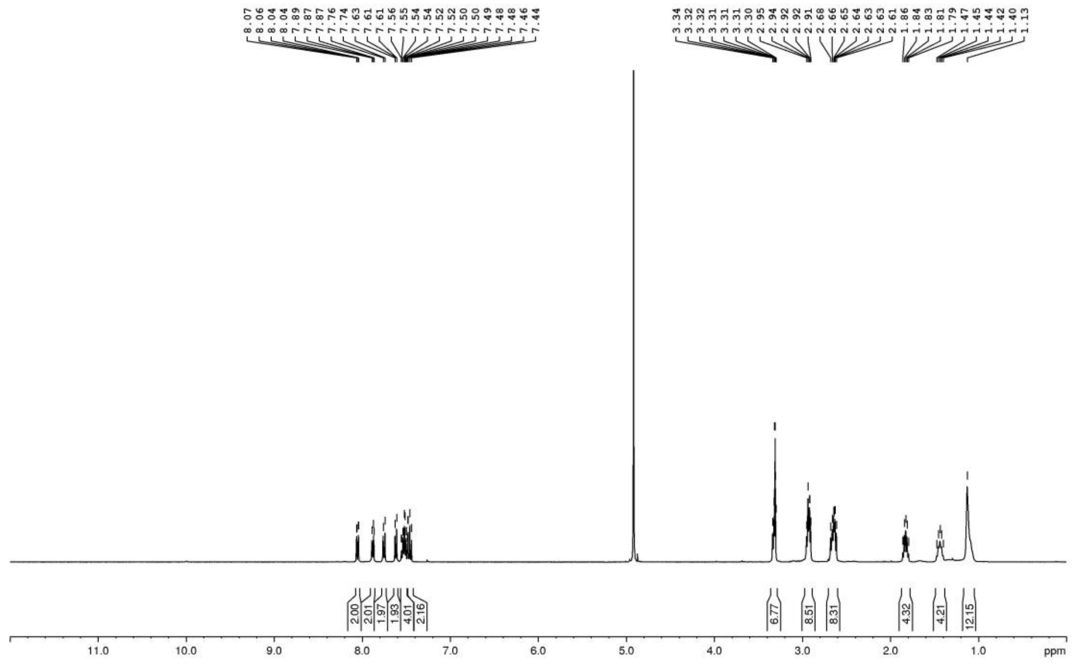

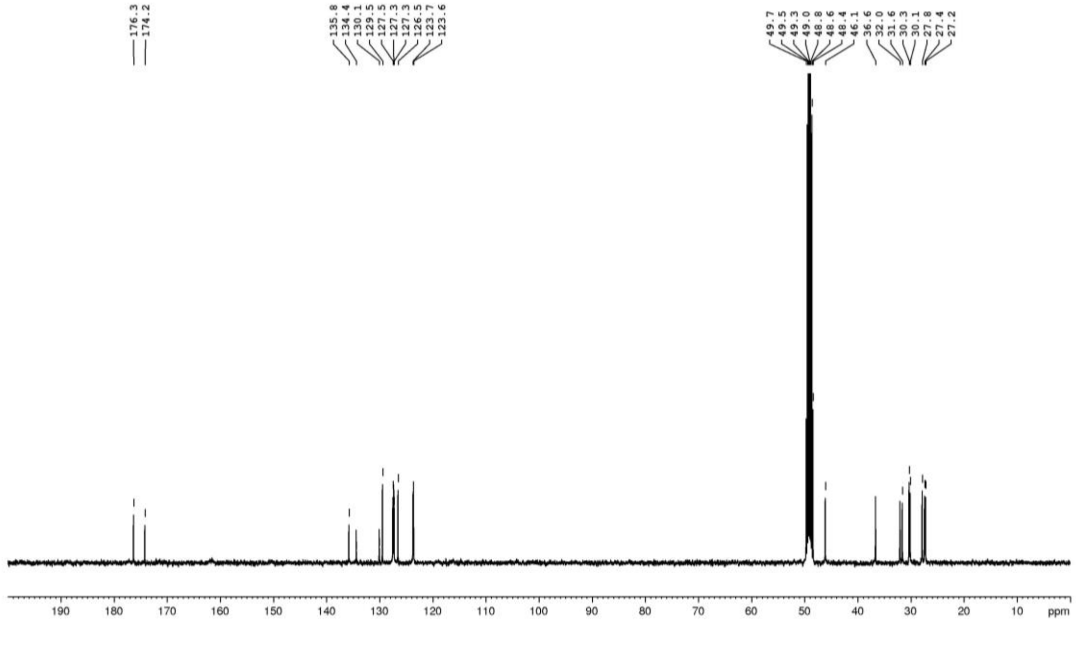

Figure S14 ^{1}H NMR (CD_3OD, 400 MHz) and ^{13}C NMR (CD_3OD, 100 MHz) spectra for **18e**

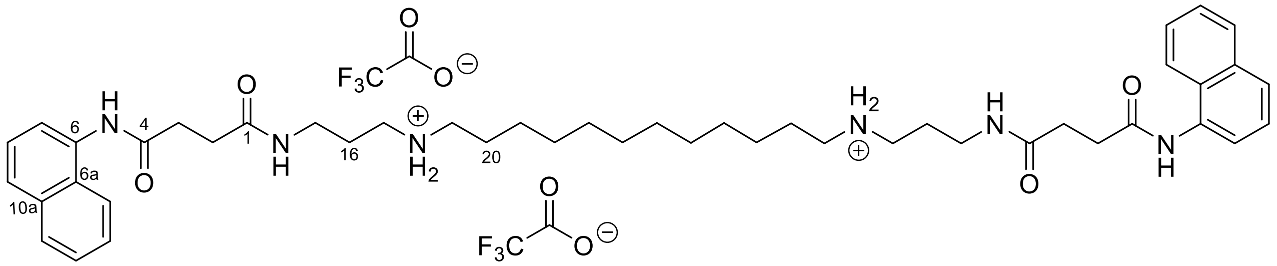

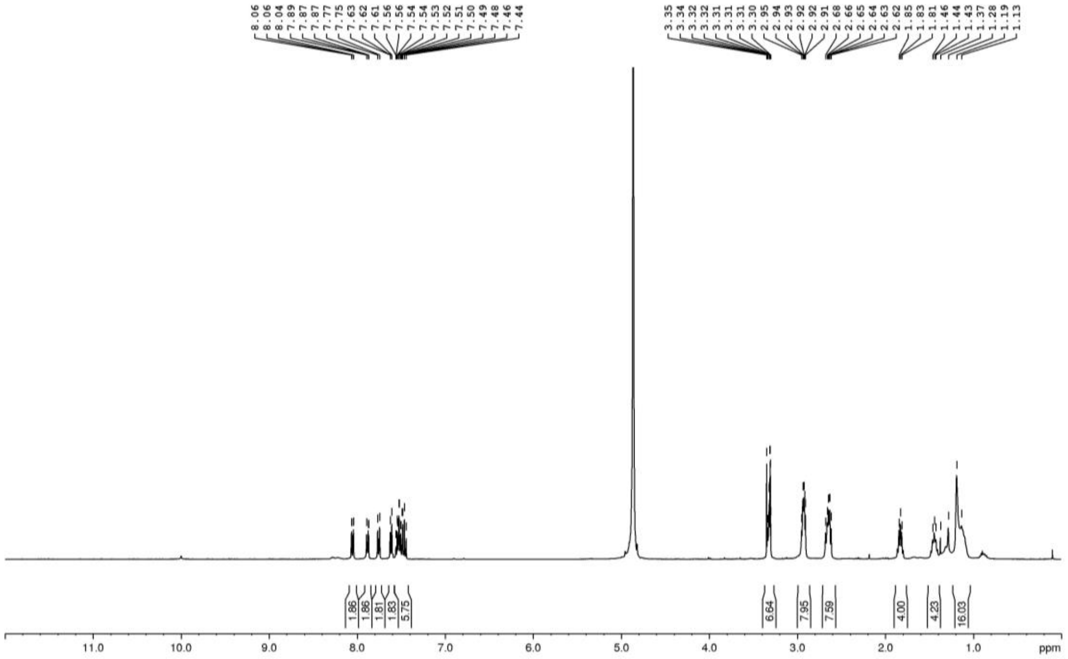

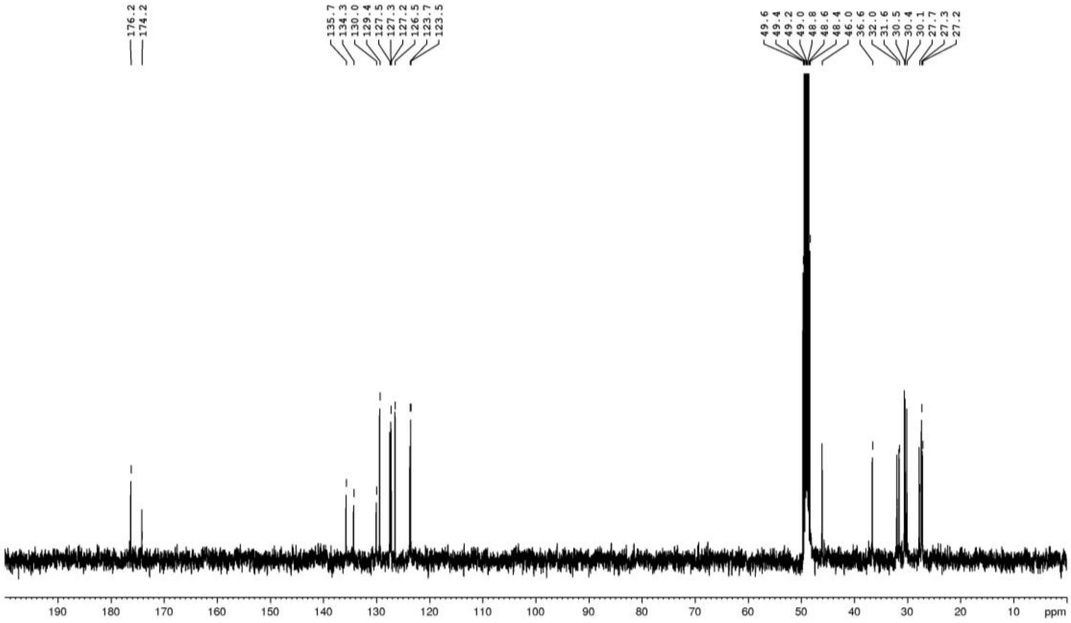

Figure S15 ^{1}H NMR (CD_3OD, 400 MHz) and ^{13}C NMR (CD_3OD, 100 MHz) spectra for **18f**

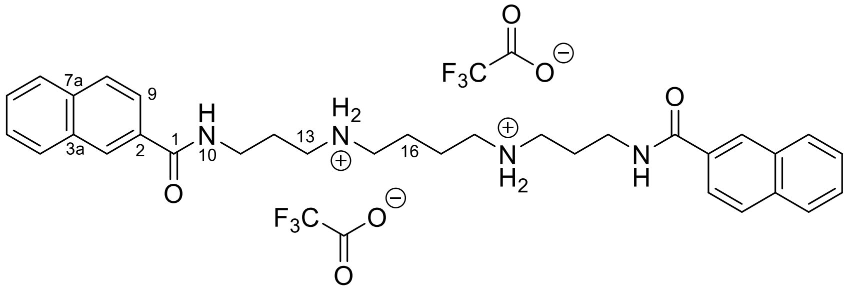

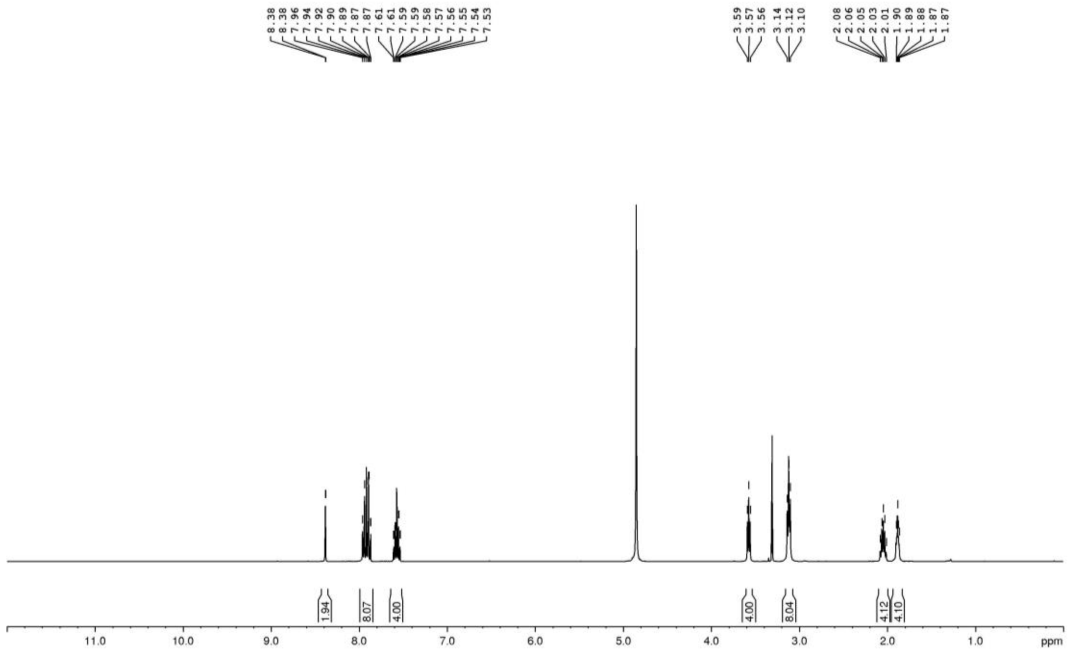

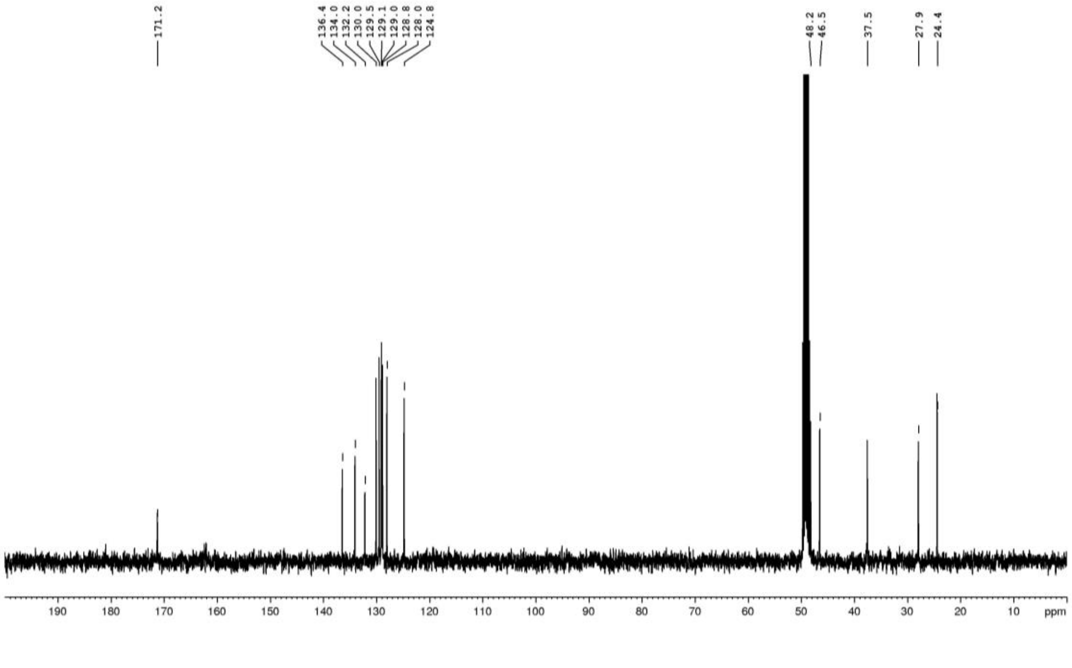

Figure S16 ^{1}H NMR (CD_3OD, 400 MHz) and ^{13}C NMR (CD_3OD, 100 MHz) spectra for **19a**

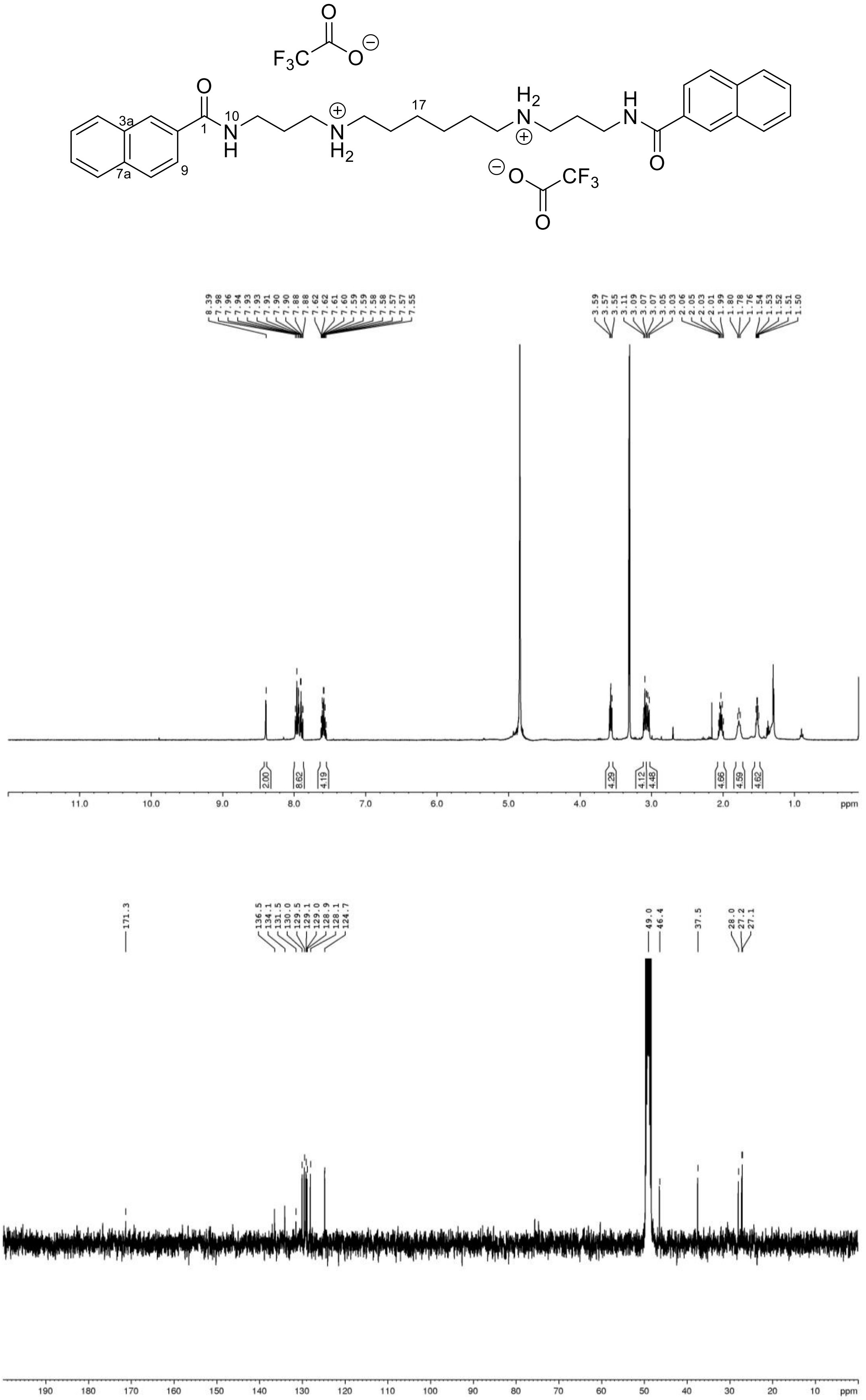

Figure S17 ^{1}H NMR (CD_3OD, 400 MHz) and ^{13}C NMR (CD_3OD, 100 MHz) spectra for **19b**

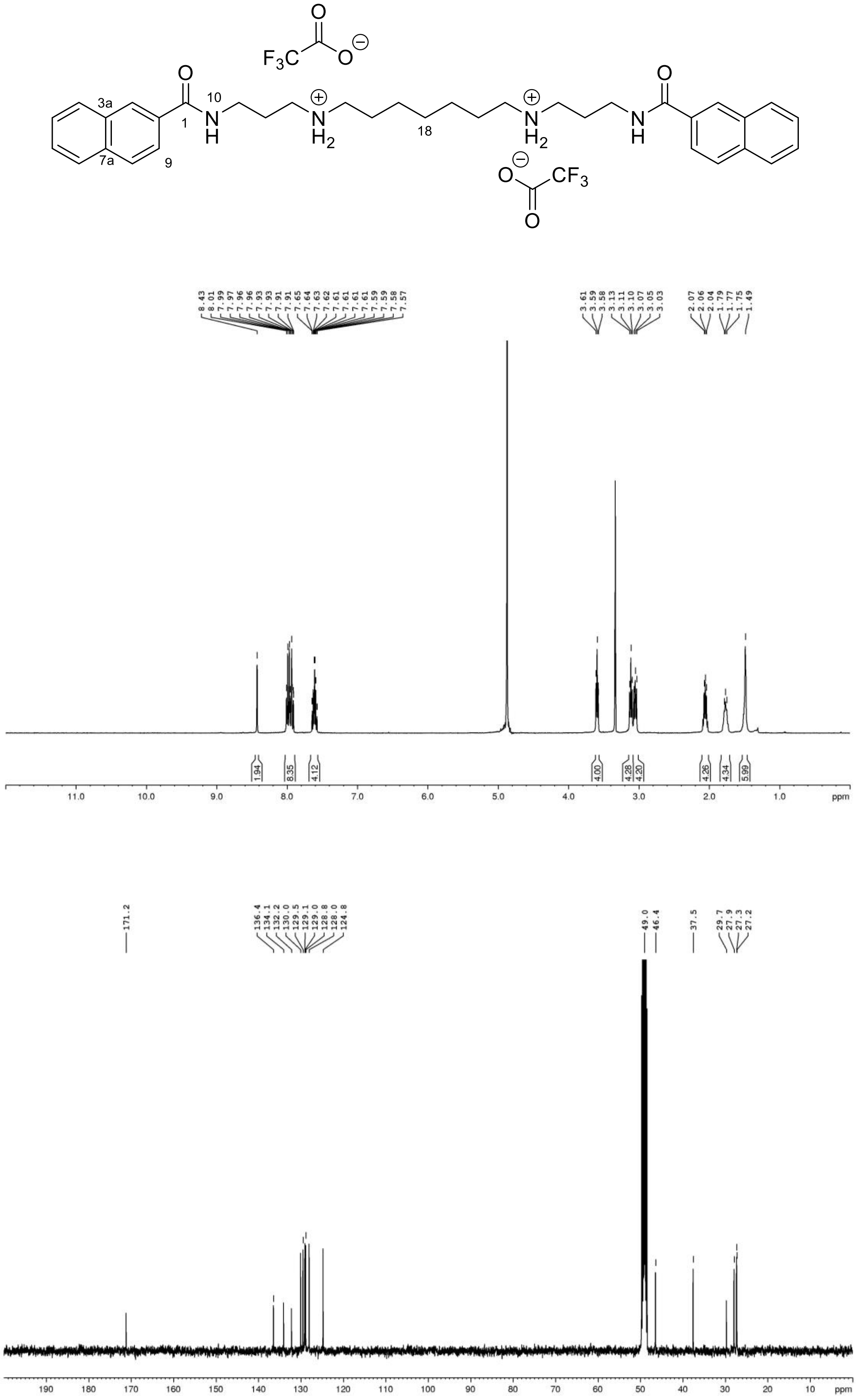

Figure S18 ^{1}H NMR (CD_3OD, 400 MHz) and ^{13}C NMR (CD_3OD, 100 MHz) spectra for **19c**

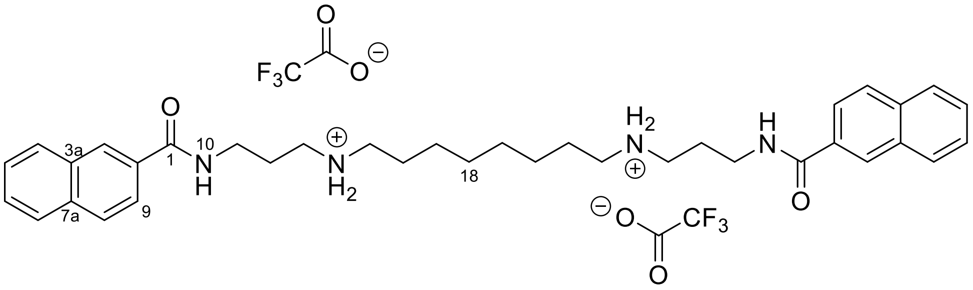

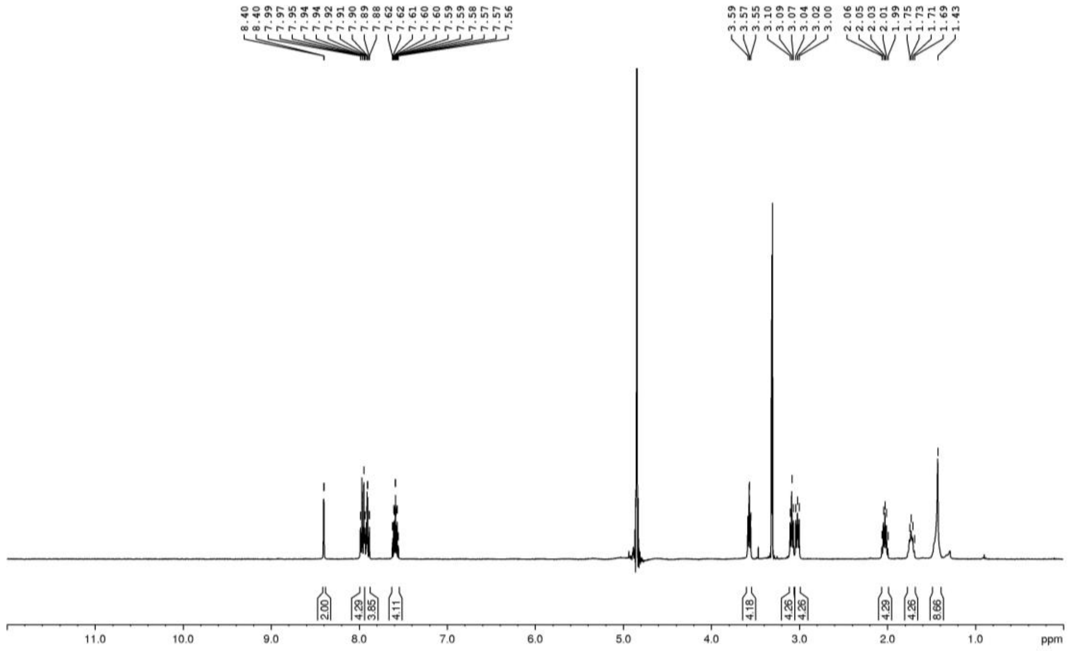

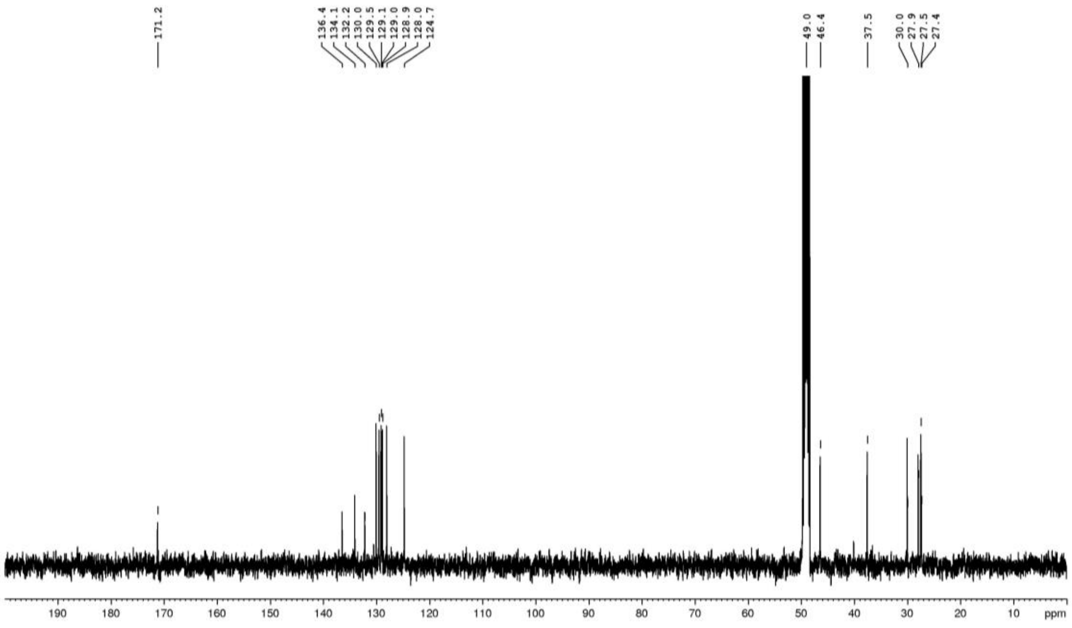

Figure S19 ^{1}H NMR (CD_3OD, 400 MHz) and ^{13}C NMR (CD_3OD, 100 MHz) spectra for **19d**

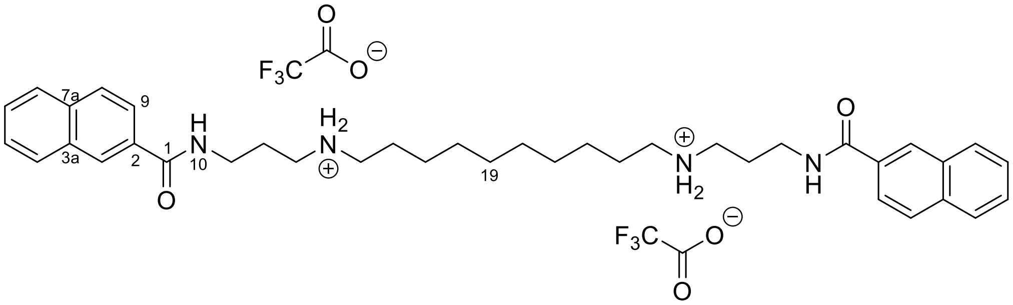

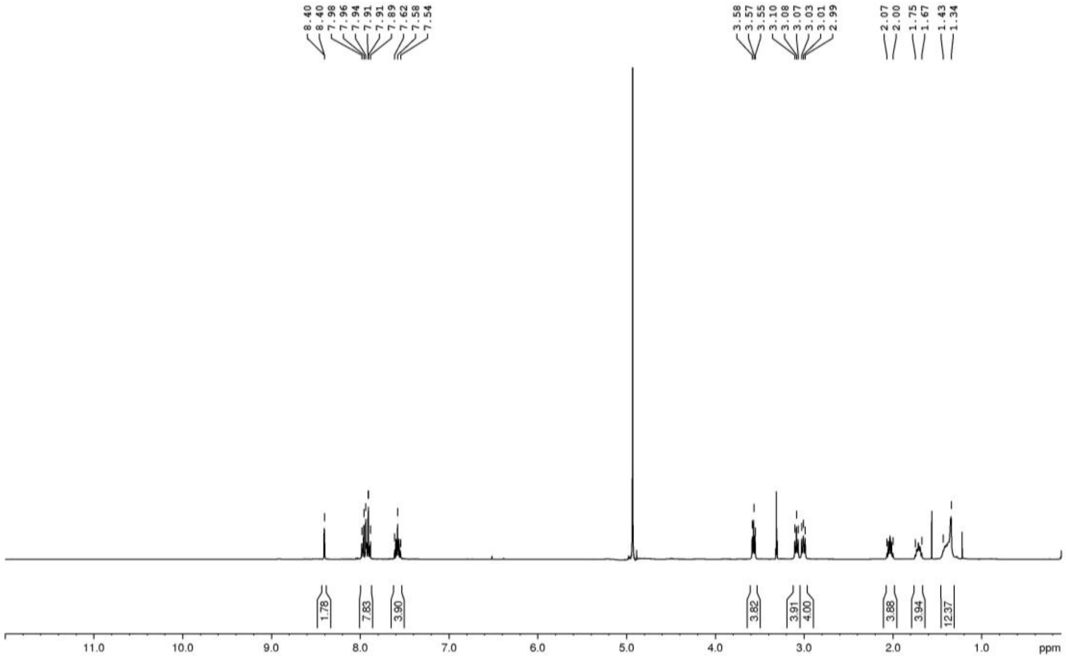

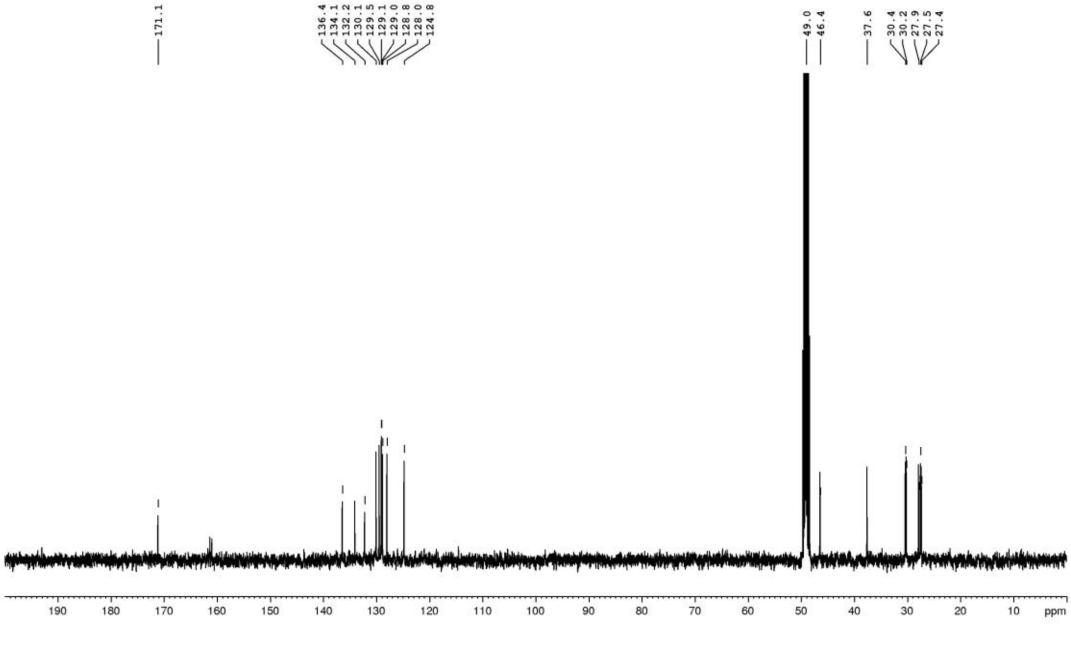

Figure S20 ^{1}H NMR (CD_3OD, 400 MHz) and ^{13}C NMR (CD_3OD, 100 MHz) spectra for **19e**

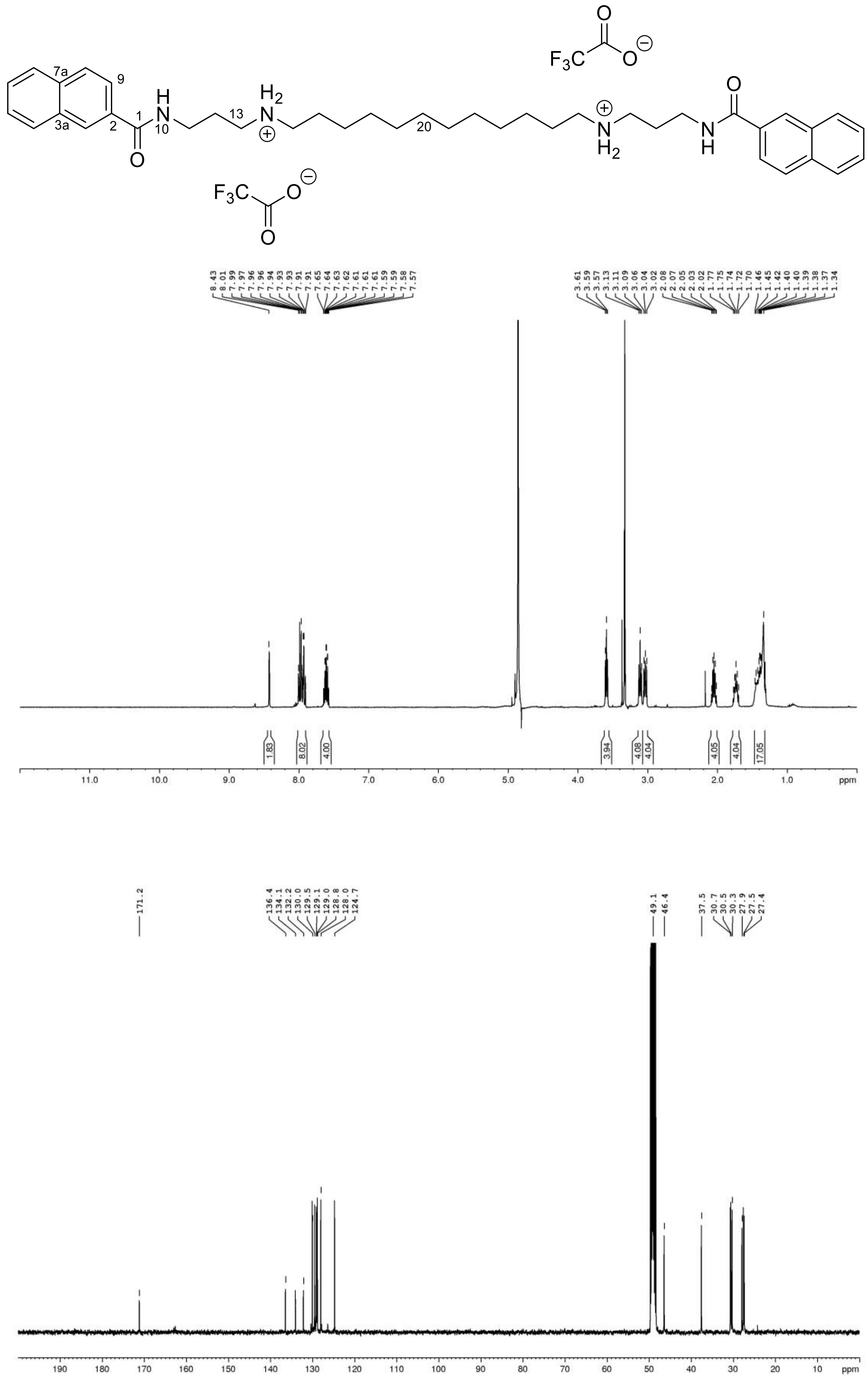

Figure S21 ^{1}H NMR (CD_3OD, 400 MHz) and ^{13}C NMR (CD_3OD, 100 MHz) spectra for **19f**

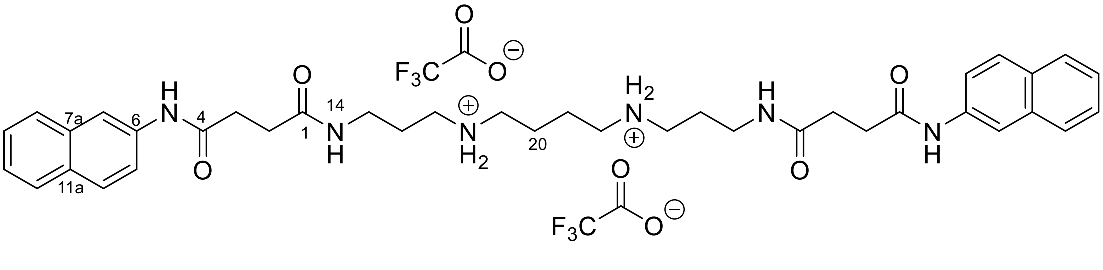

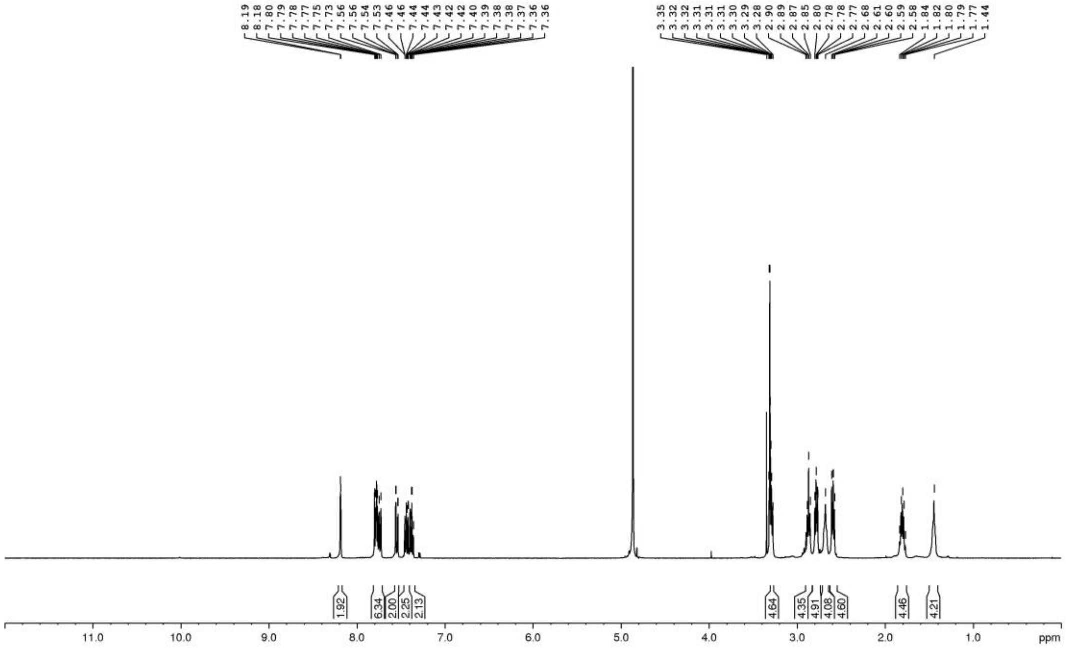

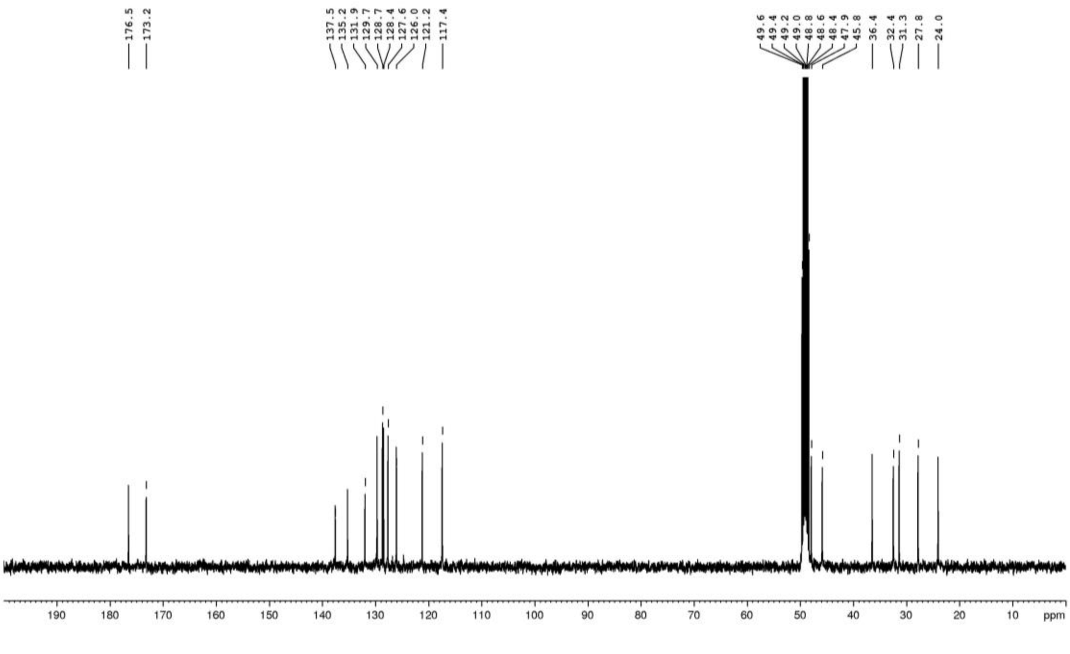

Figure S22 ^{1}H NMR (CD_3OD, 400 MHz) and ^{13}C NMR (CD_3OD, 100 MHz) spectra for **20a**

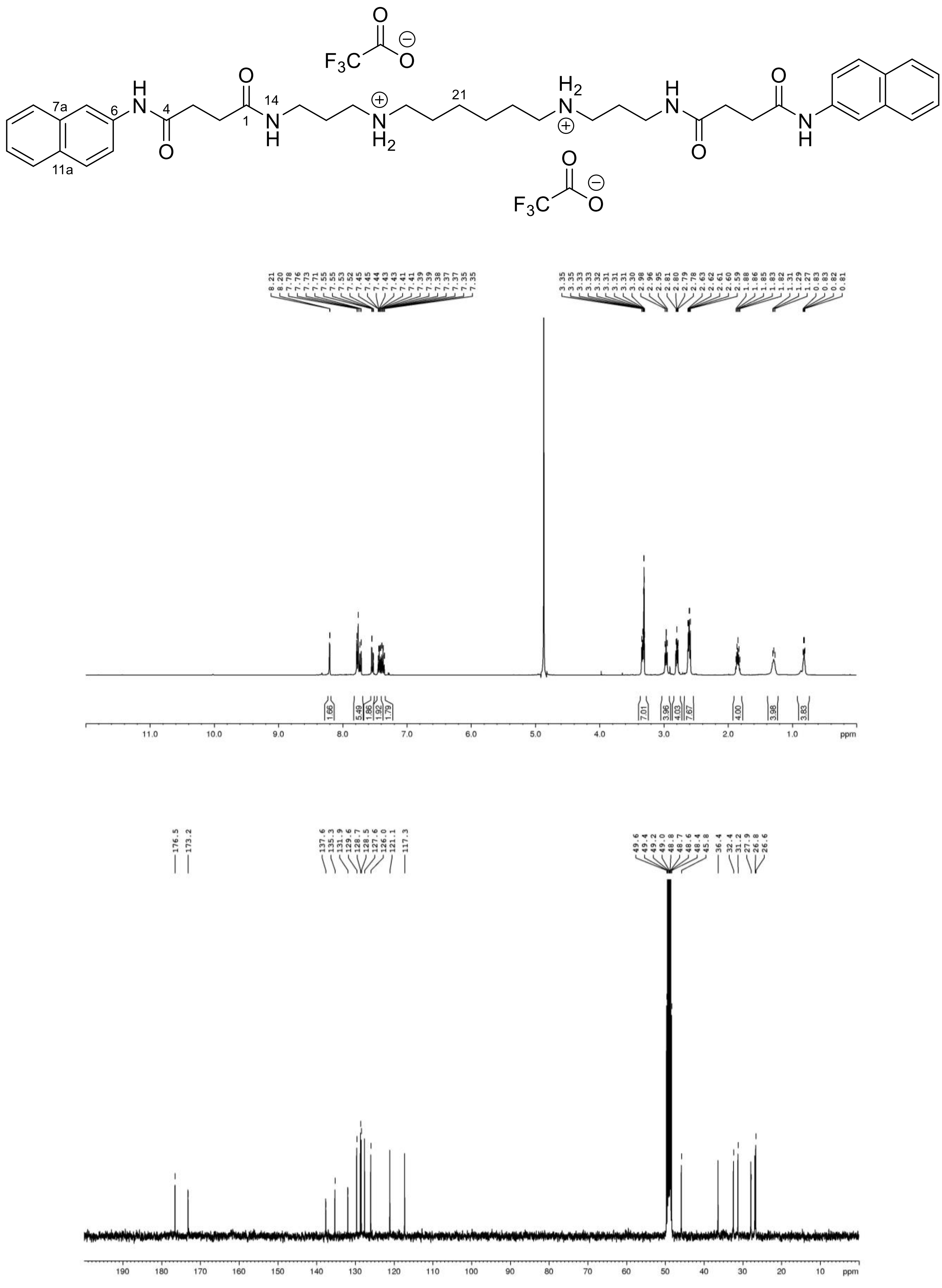

Figure S23 ^{1}H NMR (CD_3OD, 400 MHz) and ^{13}C NMR (CD_3OD, 100 MHz) spectra for **20b**

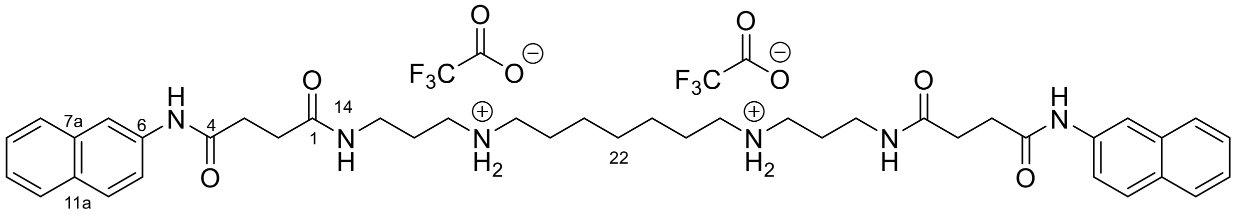

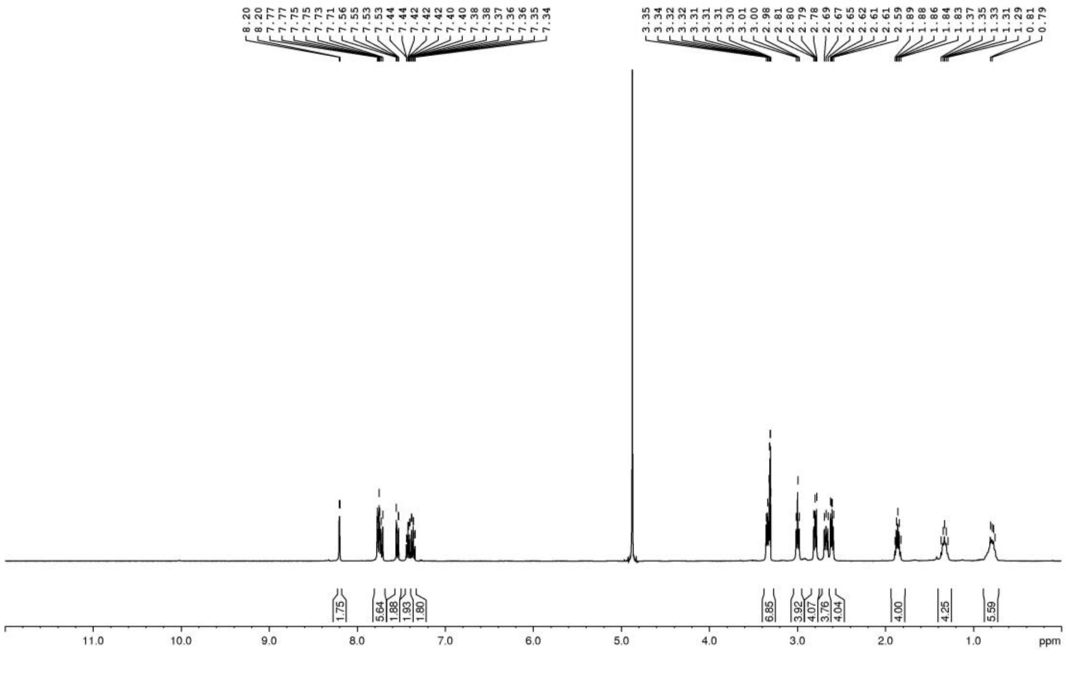

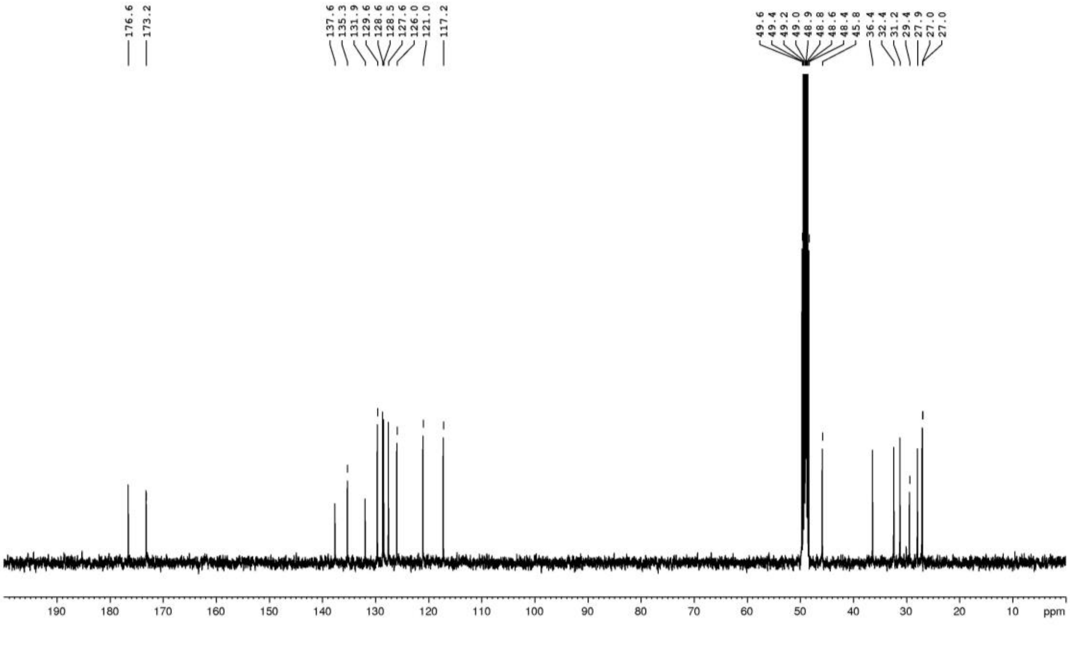

Figure S24 ^{1}H NMR (CD_3OD, 400 MHz) and ^{13}C NMR (CD_3OD, 100 MHz) spectra for **20c**

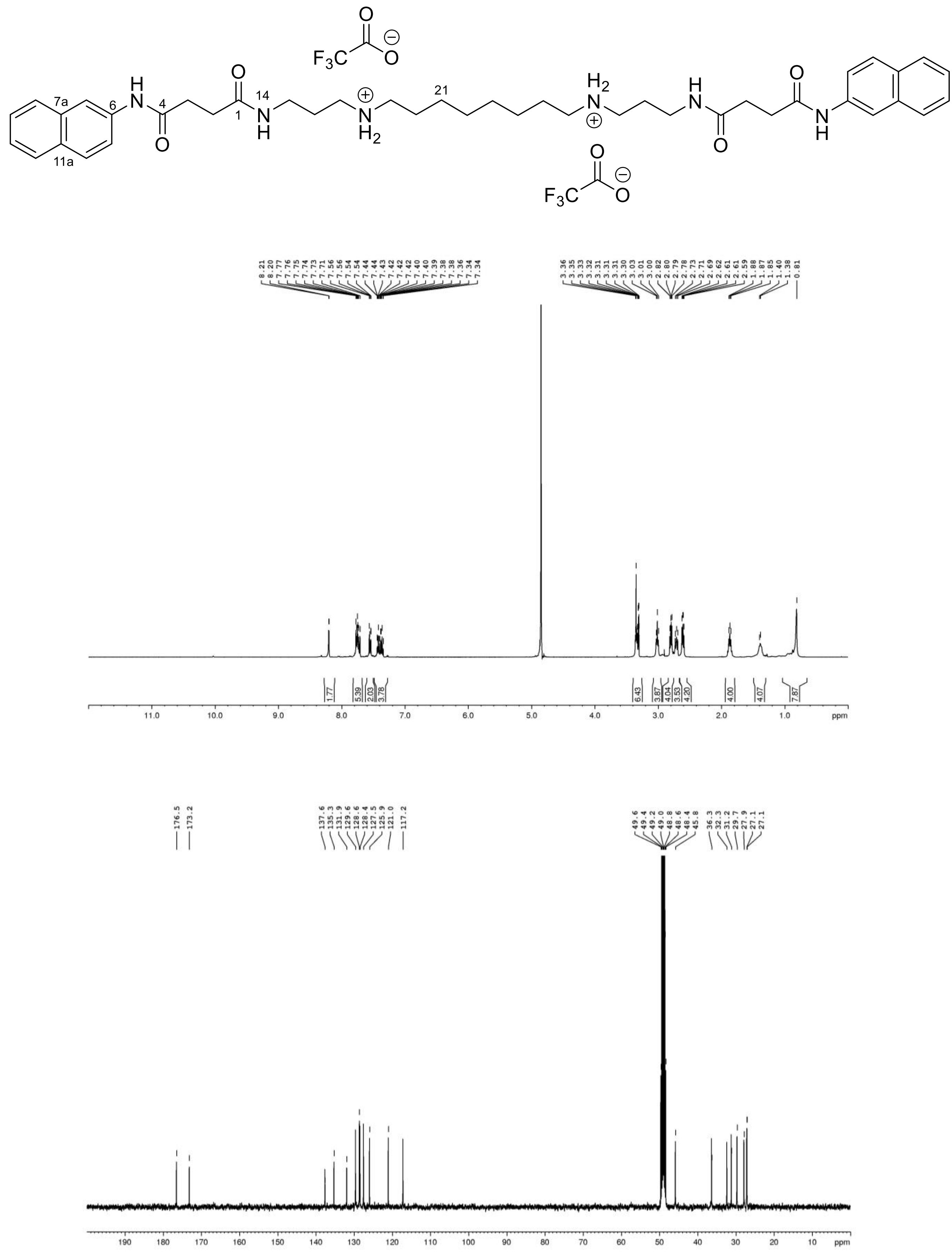

Figure S25 ^{1}H NMR (CD_3OD, 400 MHz) and ^{13}C NMR (CD_3OD, 100 MHz) spectra for **20d**

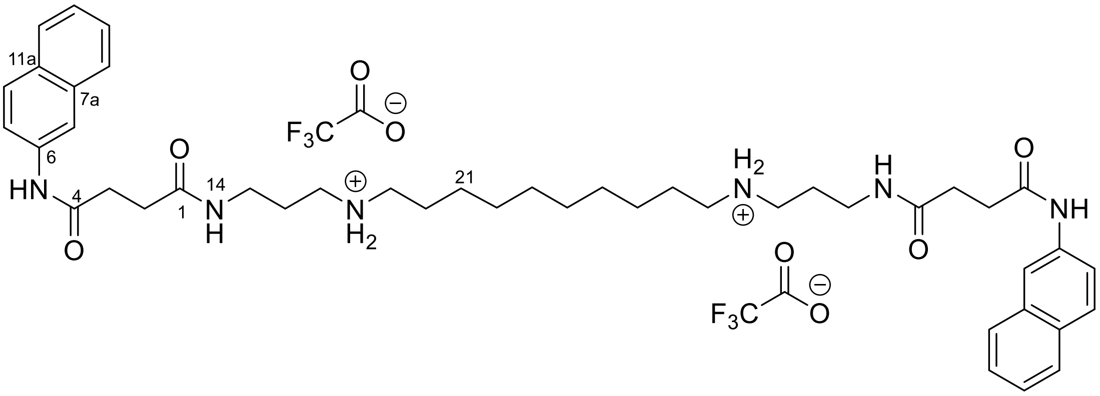

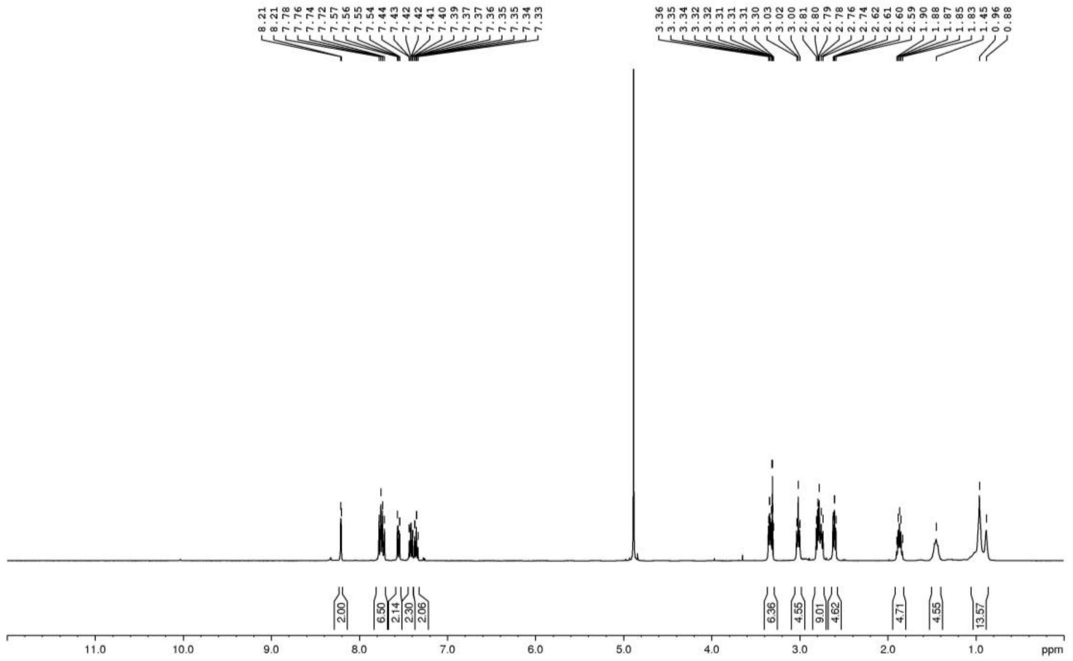

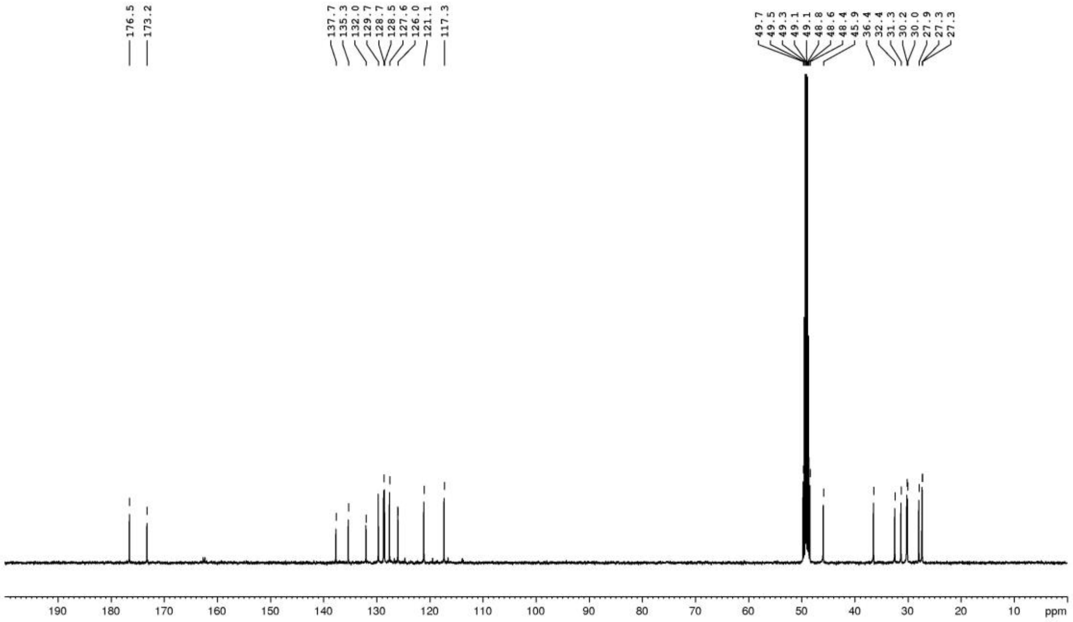

Figure S26 ^{1}H NMR (CD_3OD, 400 MHz) and ^{13}C NMR (CD_3OD, 100 MHz) spectra for **20e**

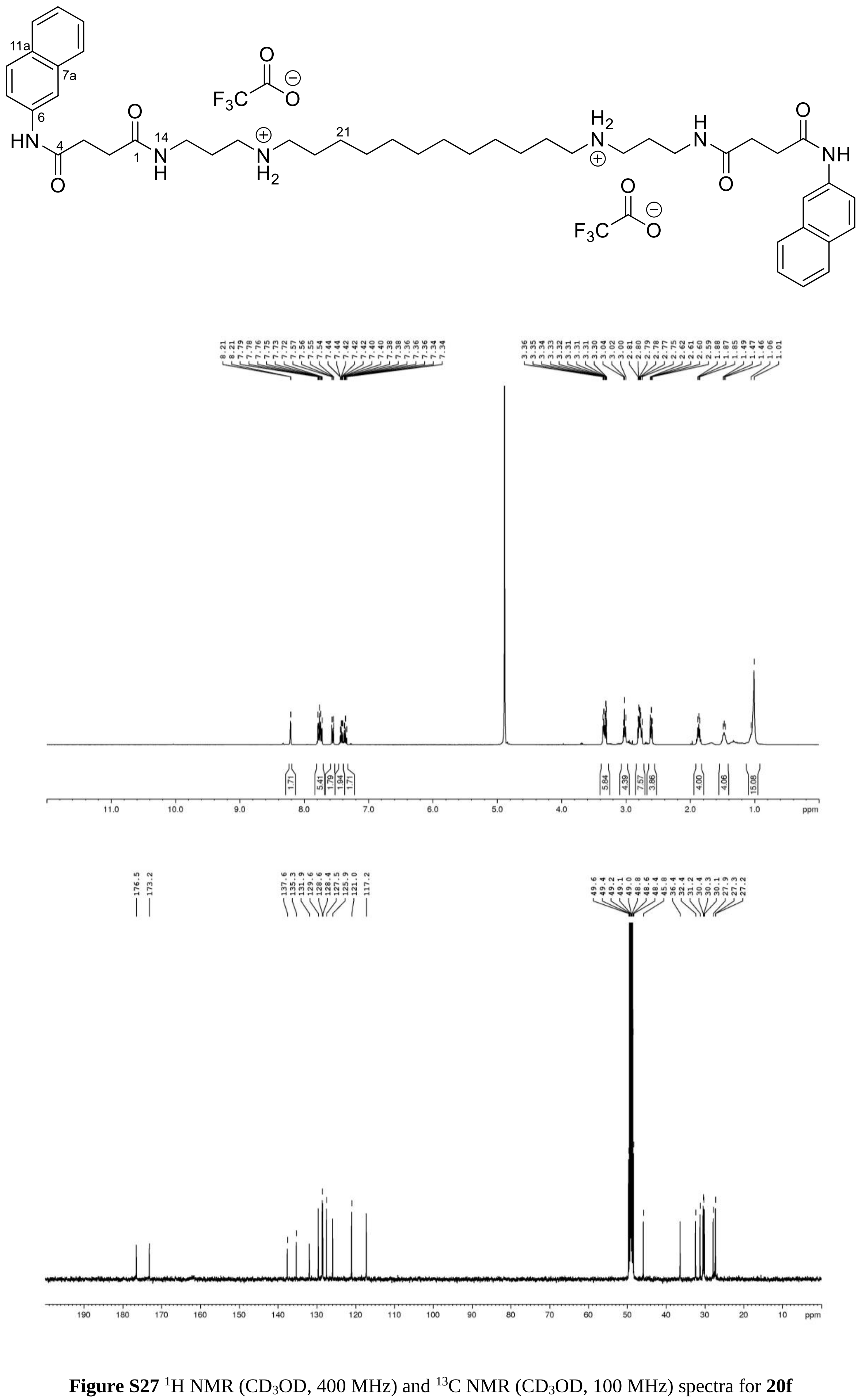

Figure S27 ^{1}H NMR (CD_3OD, 400 MHz) and ^{13}C NMR (CD_3OD, 100 MHz) spectra for **20f**

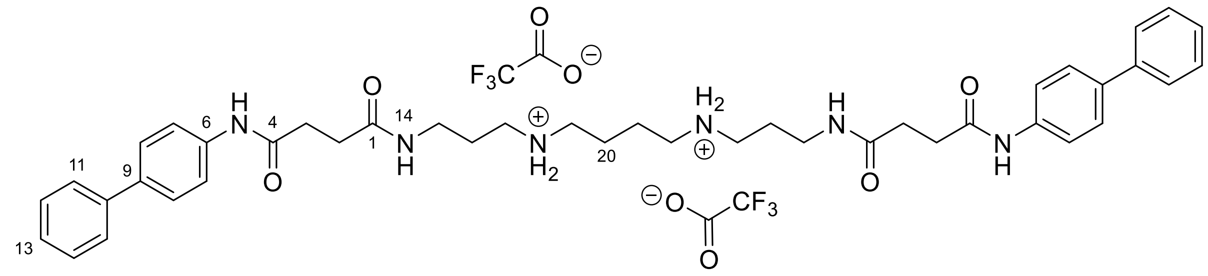

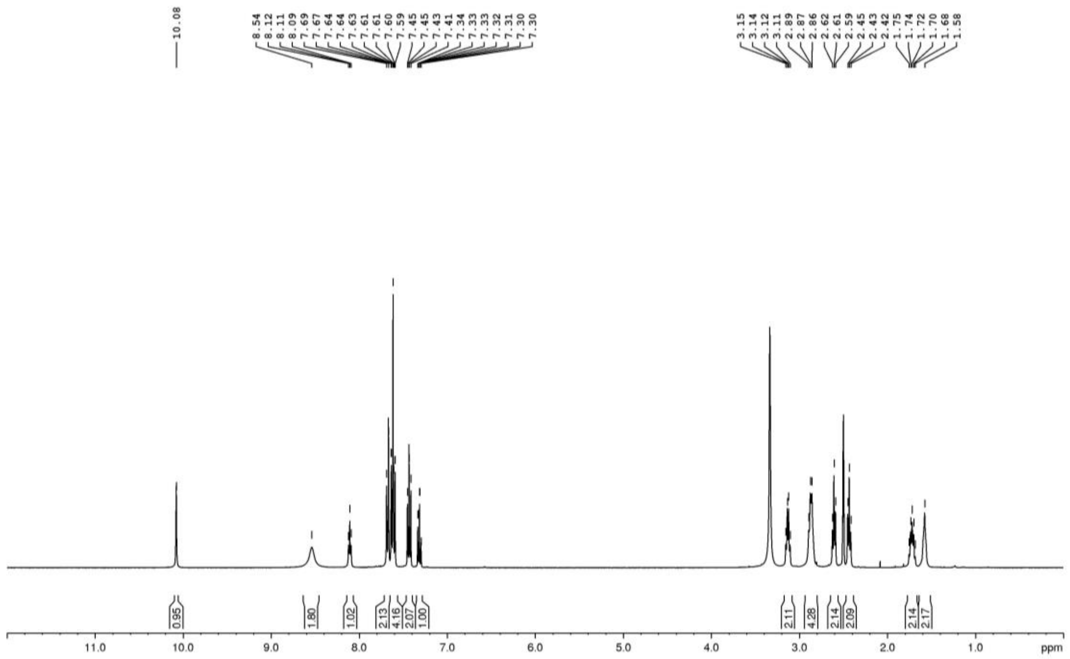

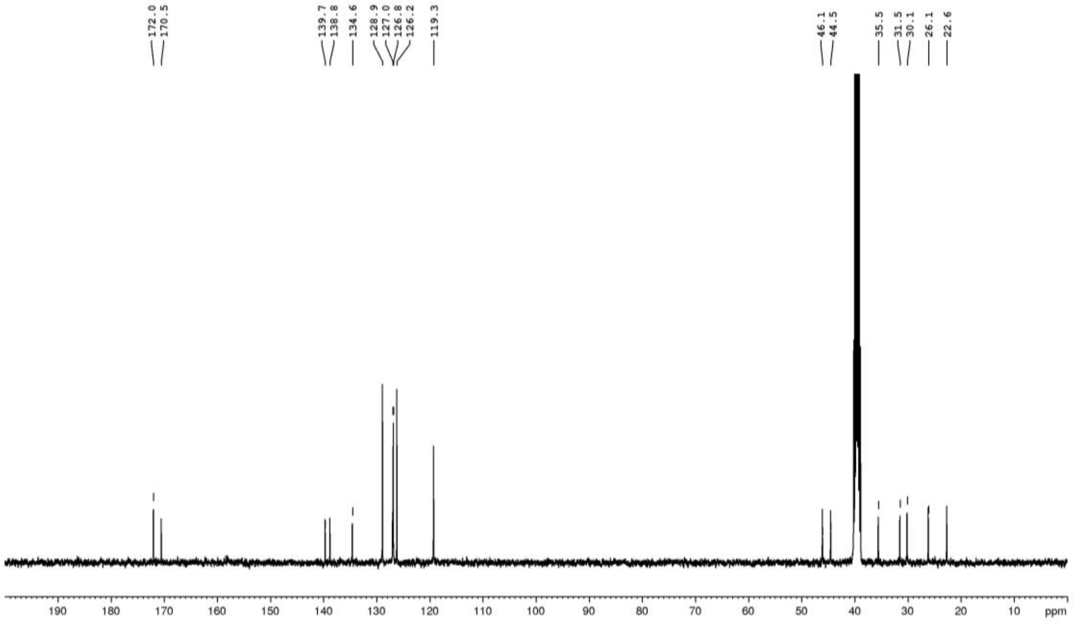

Figure S28 ^{1}H NMR (DMSO-d_6, 400 MHz) and ^{13}C NMR (DMSO-d_6, 100 MHz) spectra for **21a**

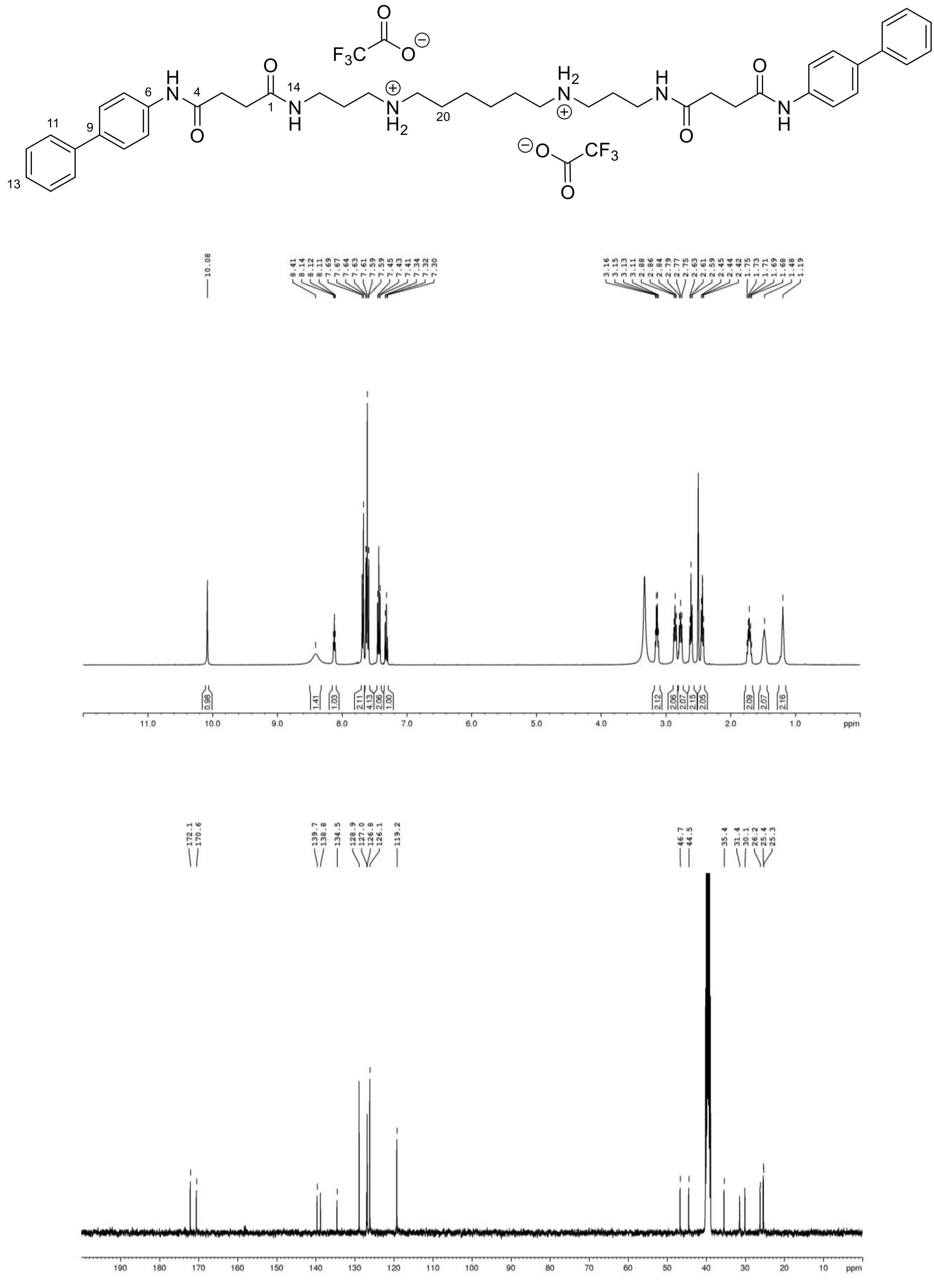

Figure S29 ^{1}H NMR (DMSO-d_6, 400 MHz) and ^{13}C NMR (DMSO-d_6, 100 MHz) spectra for **21b**

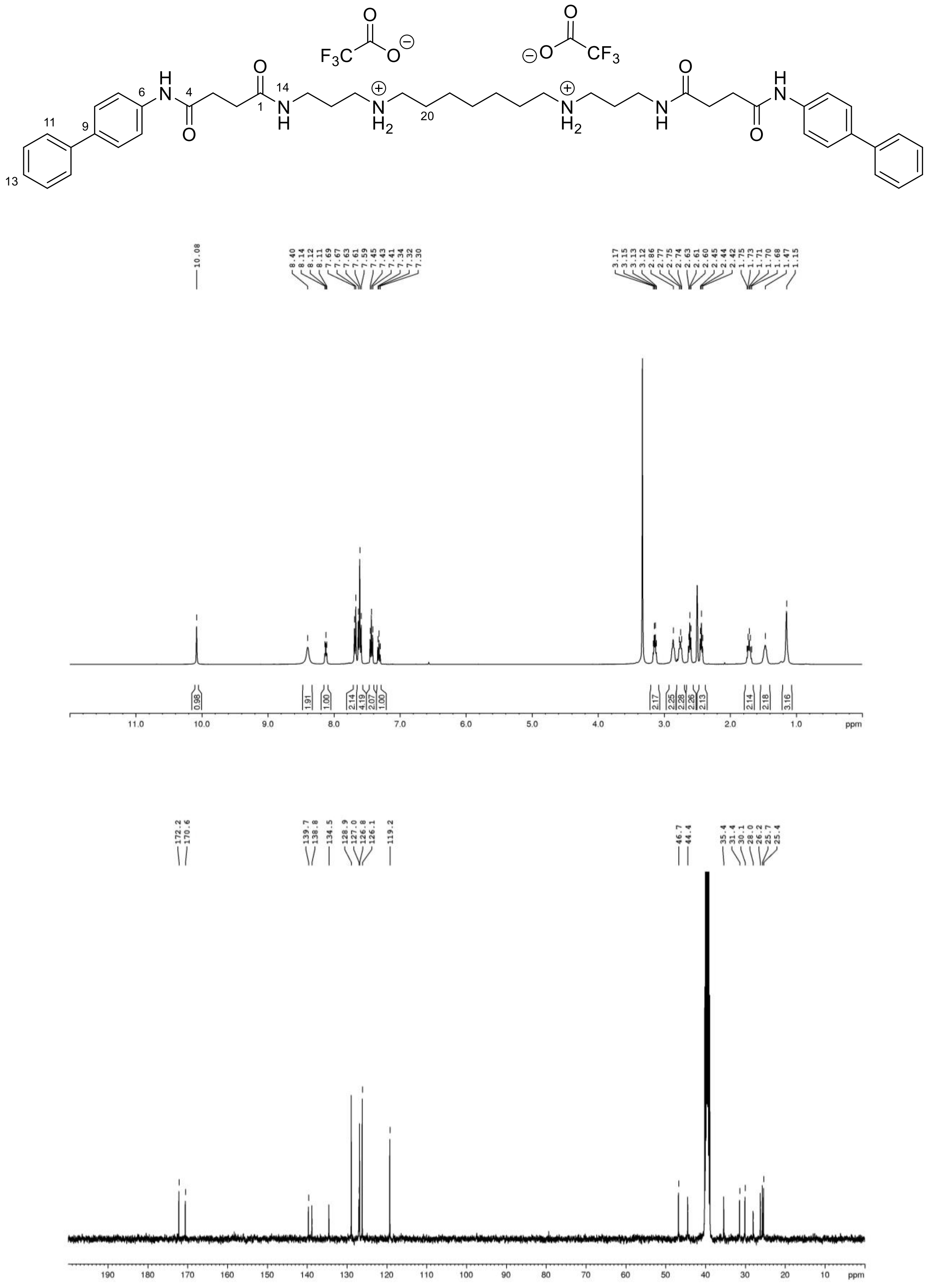

Figure S30 ^{1}H NMR (DMSO-d_6, 400 MHz) and ^{13}C NMR (DMSO-d_6, 100 MHz) spectra for **21c**

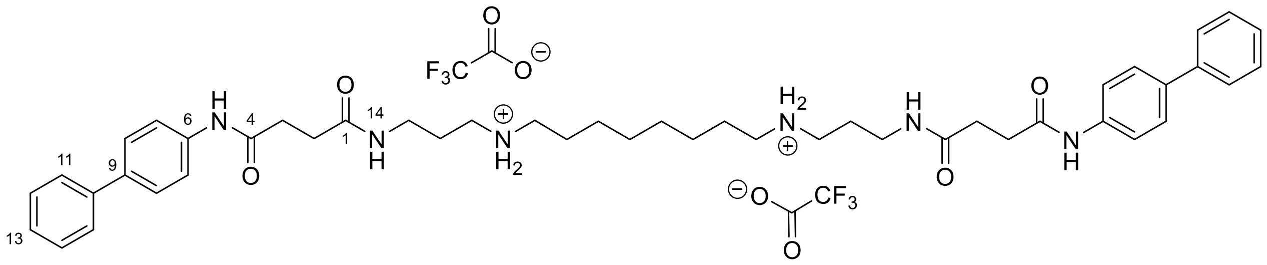

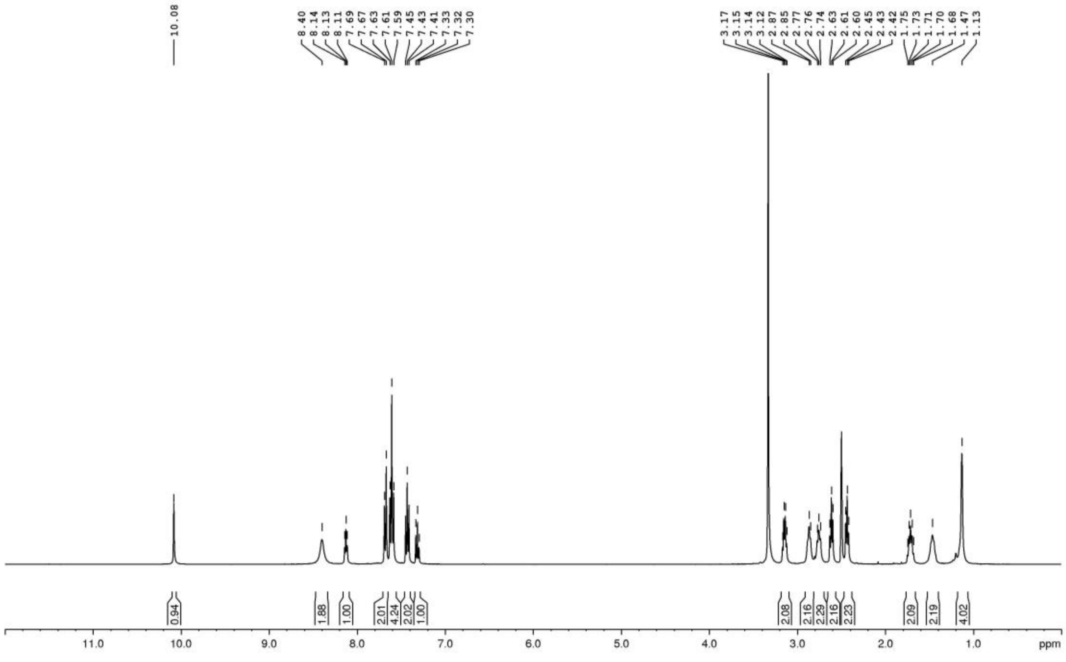

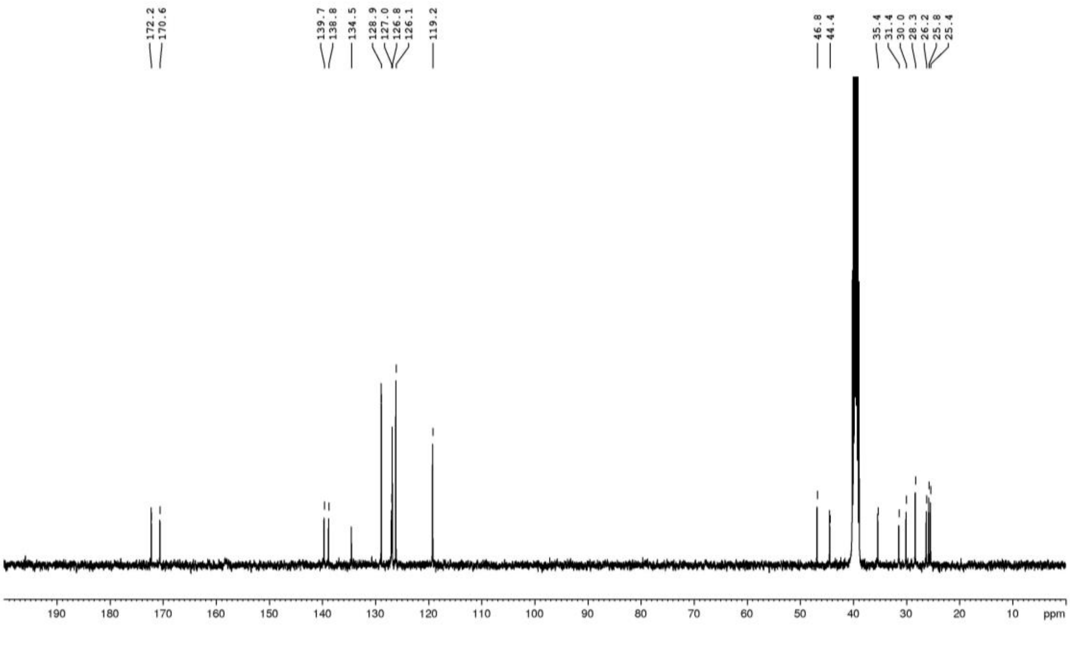

Figure S31 ^{1}H NMR (DMSO-d_6, 400 MHz) and ^{13}C NMR (DMSO-d_6, 100 MHz) spectra for **21d**

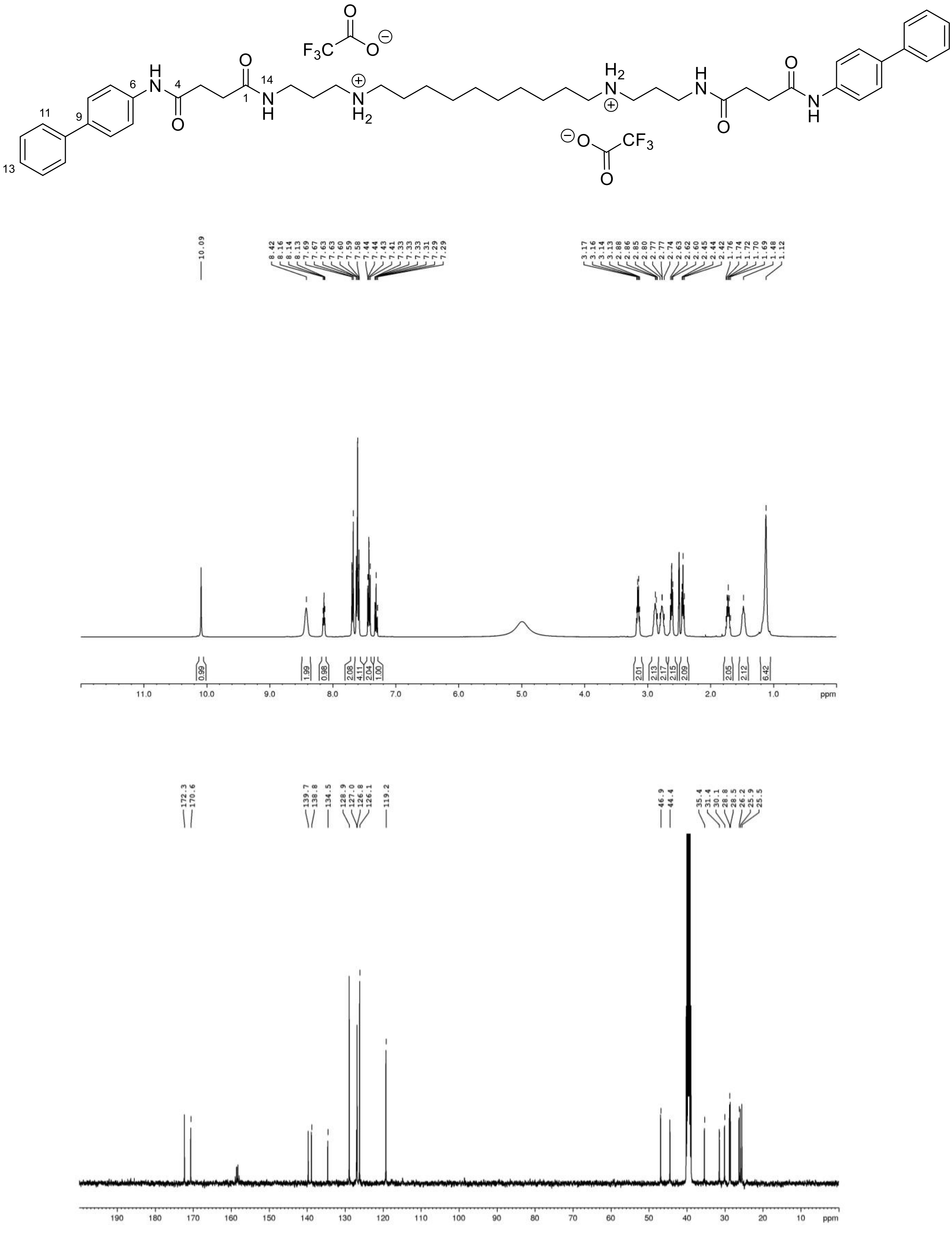

Figure S32 ^{1}H NMR (DMSO-d_6, 400 MHz) and ^{13}C NMR (DMSO-d_6, 100 MHz) spectra for **21e**
